# Renewable Reagent for Nucleophilic Fluorination

**DOI:** 10.1021/acs.joc.2c00247

**Published:** 2022-04-19

**Authors:** Blaž Alič, Jan Petrovčič, Jan Jelen, Gašper Tavčar, Jernej Iskra

**Affiliations:** † Department of Inorganic Chemistry and Technology, Jožef Stefan Institute, Jamova 39, 1000 Ljubljana, Slovenia; ‡ Department of Chemistry and Biochemistry, University of Ljubljana, Faculty of Chemistry and Chemical Technology, Večna pot 113, 1000 Ljubljana, Slovenia

## Abstract

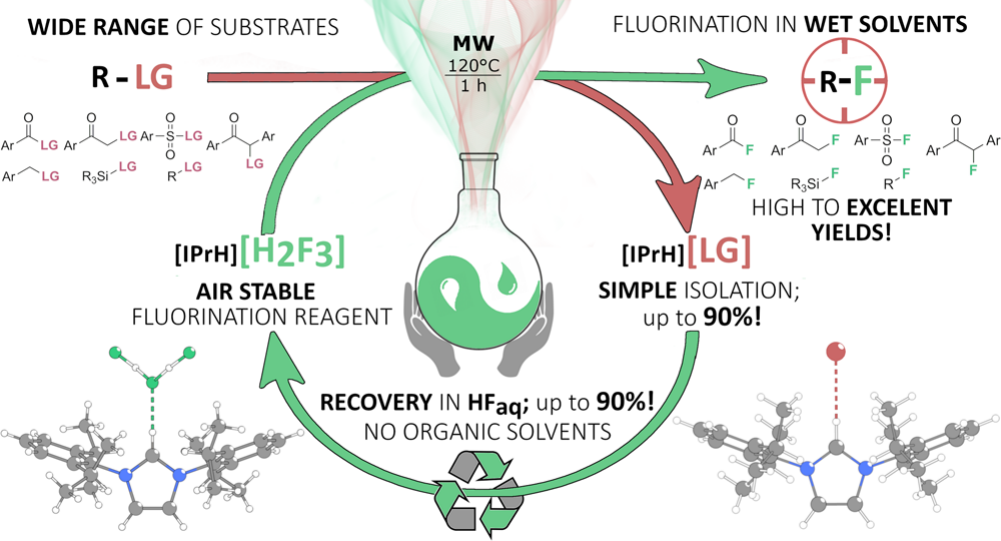

Herein, we report a study on the reactivity of three 1,3-diarylimidazolium-based fluoride reagents, with a general formula of [IPrH][F(HF)_*n*_] (*n* = 0, 1, or 2), that tackle the challenges of limited solubility, hygroscopicity, instability, and laborious preparation procedures of nucleophilic fluoride reagents. Fluorination of 4-*tert*-butylbenzyl bromide reveals that trifluoride [IPrH][F(HF)_2_] is the most selective reagent. Microwave-assisted activation coupled with the addition of sterically hindered amine DIPEA or alkali metal fluorides increases the rate of fluorination with [IPrH][F(HF)_2_], making it an excellent reagent for the fluorination of various organic substrates. The scope of substrates includes benzyl bromides, iodides, chlorides, aliphatic halides, tosylates, mesylates, α-haloketones, a silyl chloride, acyl and sulfuryl chlorides, and a nitroarene. The exceptional stability of the air-stable and nonhygroscopic [IPrH][F(HF)_2_] reagent is illustrated by its convenient synthesis and detailed experimental regeneration protocol using hydrofluoric acid without organic solvents.

## Introduction

**Chart 1:**
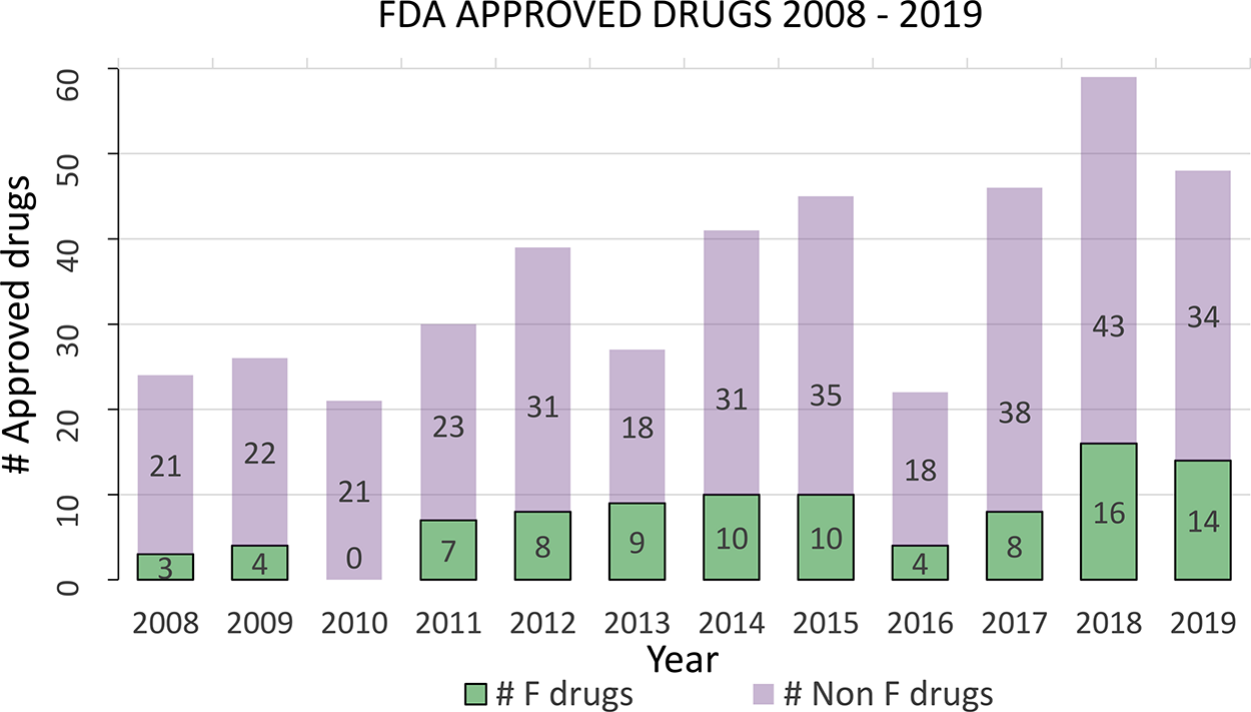
: FDA-Approved Drugs per Year in 2008–2019 (see Supporting Information S1 for Additional Data)

Fluorine’s unique properties give rise to special characteristics of fluorinated organic compounds.^1^ Inevitably, fluorinated organic compounds continue to establish themselves as an invaluable group of chemicals with major utility value in the industry, agrochemistry, pharmaceuticals, and diagnostics (PET).^2^ This is evident from a general increase in the FDA approval rate of fluorine-containing drugs in the last decade (Chart 1). The last two years were especially remarkable as 16 out of 59 FDA-approved drugs in 2018 and 14 out of 48 FDA-approved drugs in 2019 contained at least one incorporated fluorine atom. A quick calculation reveals a staggering 27% approval rate of fluorinated drugs in 2018 and 29% in 2019, which further exemplifies the need for the development of new and viable methods for fluorination.^3−5^

Nucleophilic fluorination presents a very straightforward approach to the incorporation of fluorine atoms into organic molecules.^6−9^ The general application of early nucleophilic fluoride reagents was limited due to various reasons. For example, alkali metal fluorides have been exceeded by organosoluble fluorides due to their insufficient solubility in organic solvents.^10^ However, the introduction of organosoluble fluoride reagents was accompanied by problems with their stability (*e.g.*, decomposition of TBAF *via* Hoffmann elimination).^11^ Hypervalent silicate or stannate reagents were then developed to address the stability problem, albeit at the expense of their reactivity. Consequently, these reagents have to be used in excess to compensate for their increased stability (*e.g.*, TBAT, which leads to the overall inefficiency of the fluorination process).^12,13^ Hence, the search for an easy-to-prepare reagent with the right combination of solubility, stability, and reactivity is ongoing.

Research in this direction brought some of the most ground-breaking advances in the fluorination chemistry field in the last 15 years, which include (a) the preparation of a “naked” fluoride reagent,^14^ (b) fluorination in protic solvents under hydrogen-bonding conditions,^7^ and (c) examples of asymmetric nucleophilic fluorination with alkali metal fluorides.^15^

**Scheme 1:**
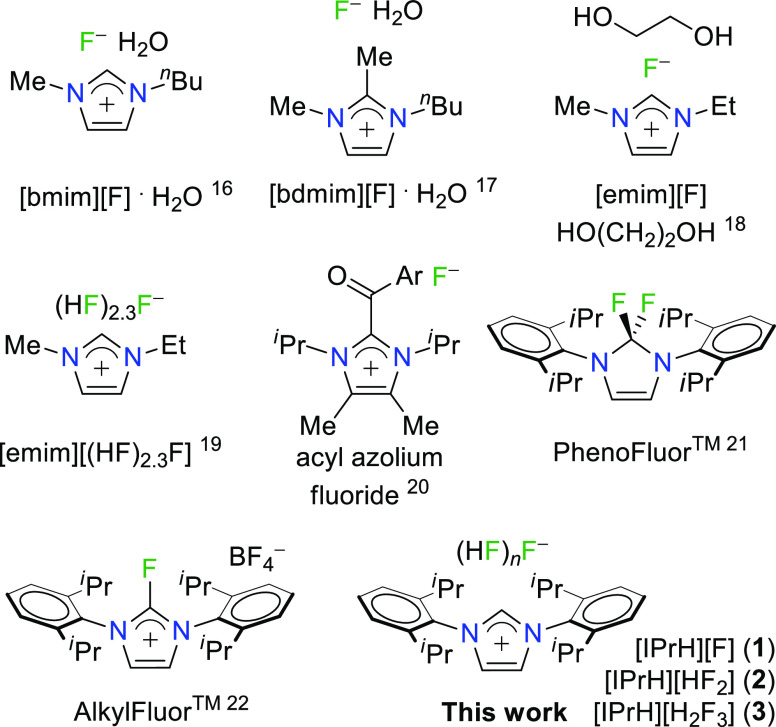
Imidazolium-Based Nucleophilic Fluoride Reagents

Fluorination with imidazolium-based fluoride reagents has been developing since the 2000s (Scheme 1). This family of reagents with desirable properties consists of ionic liquids with a fluoride anion (*e.g.*, [bmim][F]·H_2_O,^16^ [bdmim][F]·H_2_O,^17^ and [emim][F]·HOCH_2_CH_2_OH^18^), ionic liquids containing a mixture of poly(hydrogen fluoride) species (*e.g.*, [emim][F(HF)_2.3_]^19^), and *in situ* generated acyl azolium fluorides.^20^

More prominent members of this family of reagents include PhenoFluor^21^ and its air-stable successor AlkylFluor,^22^ which seem to be the reagents of choice for deoxyfluorination of phenols and aliphatic alcohols, respectively, are also based on the same malleable imidazole moiety (Scheme 1).

In late 2016, Alič and Tavčar reported the preparation of three imidazolium-based fluoride reagents, namely, [IPrH][F] (**1**), [IPrH][HF_2_] (**2**), and [IPrH][H_2_F_3_] (**3**), derived from reactions of N-heterocyclic carbene (NHC), namely, 1,3-bis(2,6-diisopropylphenyl)imidazol-2-ylidene (IPr), with different HF sources (KHF_2_, Et_3_N·3HF, and anhydrous hydrogen fluoride (aHF)) in the corresponding stoichiometries.^23^ To date, reagent **1** has been successfully used for the preparation of the first, discrete, trigonal-bipyramidal [GeF_5_]^−^ and square-pyramidal [VOF_4_]^−^ anions, where bulky imidazolium moiety, with its specific steric effects, is crucial for their stabilization.^24,25^ Although **1** is a novel tool in inorganic chemistry, its reactivity with organic substrates remained largely unexplored.

In this work, we compare the reactivity of three fluorination reagents derived from 1,3-diarylimidazolium chloride, [IPrH][Cl] (**9**), a common precursor for the preparation of N-heterocyclic carbenes (NHCs). The prepared reagents with a general formula of [IPrH][F(HF)_*n*_] (**1**–**3**, Scheme 1) were tested for the nucleophilic fluorination of various substrates. After successful fluorination reactions, we managed to isolate and recycle reagent **3**. Recycling was performed with hydrofluoric acid alone, without the use of organic solvents. In this way, we try to demonstrate a more sustainable and greener fluorination process with reduced waste.

**Table 1:**
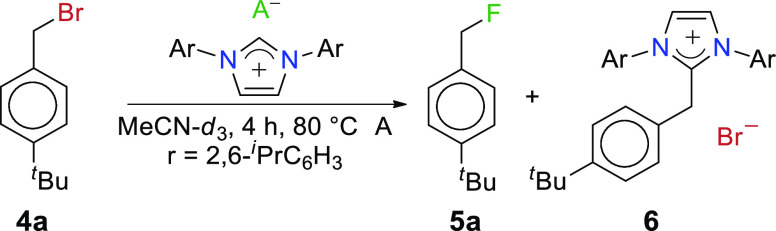
Competition between F^–^ and IPr (NHC) Nucleophiles in Reactions of [IPrH][F(HF)*_n_*] with **4a**

reagent	[A]^−^	conv. [%]^a^	5a [%]^a^	6 [%]^a^
1	[F]^−^	100	8	92
2	[HF_2_]^−^	45	39	6
3	[H_2_F_3_]^−^	18	18	/

a Product distribution was determined by ^1^H NMR spectroscopy.

## Results and Discussion

Due to encouraging results obtained from the reactions of [IPrH][F] (**1**) with different inorganic substrates, we wanted to further explore the prospects of the aforementioned reagent. We assumed that **1** would have the highest reactivity for the fluorination of organic substrates because the reactivity of poly(hydrogen fluoride) species decreases with the increasing number of associated hydrogen-bonded HF molecules.^26^ 4-*tert*-Butylbenzyl bromide (**4a**) was chosen as a model substrate to test the reactivity of **1**–**3** (Table 1).

**Scheme 2:**
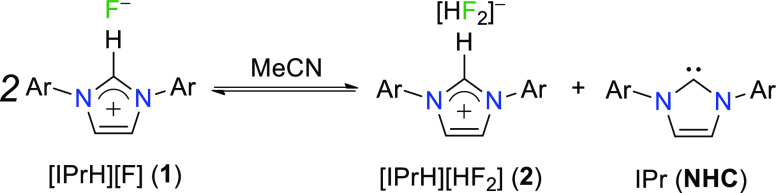
[IPrH][F] (**1**) in Equilibrium with IPr and [IPrH][HF_2_] (**2**)

The yield of 4-*tert*-butylbenzyl fluoride (**5a**) obtained with reagent **1** is low due to an unexpected competitive reaction of the *in situ* formed IPr (NHC), with the model substrate **4a** forming side product **6** (Table 1). The *in situ* formed IPr acts as a competitive nucleophile to fluoride and forms side product **6** (see Supporting Information S10). The presence of side product **6** additionally substantiates the findings of the original research from Alič and Tavčar in which they noted an equilibrium between [IPrH][F] (**1**), IPr, and [IPrH][HF_2_] (**2**) in acetonitrile (Scheme 2).^23^

**Table 2:**
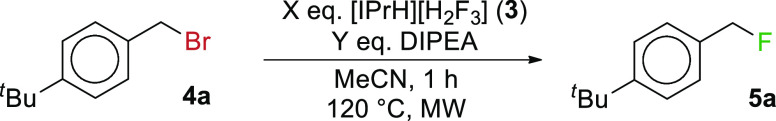
Optimizing the Ratio of the Reagent [IPrH][H_2_F_3_] (**3**) and DIPEA

#	*X* eq **3**	*Y* eq DIPEA	conv. [%]^a^	5a [%]^a^
1	1	0	48	23
2	0.5	0	33	28
3	0.5	0.5	78	74
4	0.5	1.0	77	75
5	0.5	1.7	92	87
6	0.5	3.2	97	86
7	0.6	1.8	96	95

a Conversion and yields were determined by 1H NMR spectroscopy with naphthalene as an internal standard.

Replacing reagents **1** with **2** resulted in an increased yield of fluorinated product **5a**, accompanied by a lower overall conversion (45%) (Table 1). Additional HF molecules in **2** shifted the equilibrium away from the formation of the free IPr in the solution, although a few percent of side product **6** could still be observed. To further reduce the yield of **6**, reagent [IPrH][H_2_F_3_] (**3**) was used to fluorinate substrate **4a**. Initially, a small conversion (18%) was observed; however, the only reaction channel was fluorination to **5a**, and no side product **6** was observed. Despite the highest reactivity of **1**, reagent **3** was chosen as the most promising candidate for additional research due to its highest chemoselectivity and ease of preparation (see Supporting Information S3 and S8).

We wanted to retain the selectivity of **3** and increase its reactivity at the same time. To reduce reaction times, we switched from conventional to microwave heating. Individual F(HF)_*n*_^–^ anions have been successfully activated using different amines (*e.g.*, pyridine, Et_3_N, etc.) or alkali metal fluorides by means of shifting equilibria between poly(hydrogen fluoride) species toward free fluoride anion.^27−30^ However, pyridine or triethylamine could not be used to activate **3** since they both formed quaternary ammonium salts with benzylic substrate **4a**. Hence, a sterically hindered organic amine was required and *N*,*N*-diisopropylethylamine (DIPEA) was chosen for testing. The addition of substoichiometric amounts of DIPEA resulted in a dramatic increase in the reaction yields (from 28 to 74%, Table 2, entries 2 and 3). To further improve the reaction yield, we sought the optimal ratio of DIPEA and **3** (Table 2). To achieve excellent yields (>90%), the ratio of the **3** had to be raised to 0.6 equiv (1.8 equiv of fluoride) and that of DIPEA to 1.8 equiv (entry 7).

DIPEA shows exceptional characteristics in this reaction system; its low basicity prevents *in situ* formation of N-heterocyclic carbene nucleophile, while its steric hindrance ensures good substrate compatibility. Therefore, it promotes the fluorination process while not participating in two competing side reactions. For details, see Supporting information S4; the activation of reagent **3** and S11.

Optimized reaction conditions were tested on a series of benzylic substrates with different leaving groups. A wide range of benzylic compounds was fluorinated in good to excellent yields (Table 3).

**Table 3:**
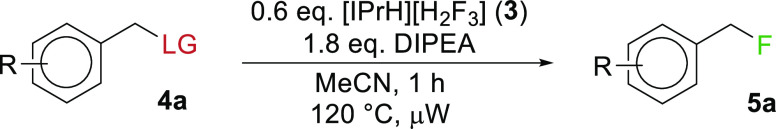
Fluorination of Benzylic Substrates with [IPrH][H_2_F_3_] (**3**) under Optimized Reaction Conditions

subs.	*R*	LG	prod.	yield [%]^a^
4a	(4-tBu)	Br	5a	95 (93)
4b	(4-Me)	Br	5b	89 (72)
4c	(3-Ph)	Br	5c	88 (90)
4d	(4-Br)	Br	5d	82 (84)
4e	(3-Br)	Br	5e	84
4f	(4-F)	Br	5f	85
4g	(4-EtOOC)	Br	5g	80 (83)
4h	(3,5-CF_3_)	Br	5h	93 (82)
4i	(4-NO_2_)	Br	5i	74 (76)
4j	(3-MeO)	Br	5j	79
4k	(3,5-MeO)	Br	5k	b
4l	(4-Me)	I	5l	81
4m	(4-Cl)	I	5m	75
4n	(2,6-Cl)	I	5n	56
4o	(4-HOOC)	Cl	5o	c
4p	(2,4-NO_2_)	Cl	5p	d
4q	(4-MeO)	Cl	5q	48
4r	(4-Me)	Cl	5r	20
4s	(2,6-Cl)	Cl	5s	27/88^e^
4t	(4-H)	OMs	5t	76
4u	(4-NO_2_)	OTs	5u	78

a Yields were determined by ^1^H NMR spectroscopy with naphthalene as an internal standard. Yields in parenthesis are isolated yields on a 0.5 mmol scale.

b Substrate reacts with DIPEA.

c Polymerizes upon contact with the reagent.

d Polymerizes during the reaction.

e Reaction with 2 equiv of **3** and 6 equiv of DIPEA. Conversion and yields were determined by ^1^H NMR spectroscopy with naphthalene as an internal standard.

Fluorination is effective with many different leaving groups including bromide, iodide, mesylate, and tosylate (Table 3). Benzyl chlorides are less reactive (**4o**–**4s**), and the corresponding fluorinated products were obtained only in 20–48% yields under the optimized reaction conditions. However, fluorination yields of benzyl chlorides can be increased by increasing the amount of reagent **3** to 2 equiv and DIPEA to 6 equiv. For example, the fluorination yield of sterically more hindered substrate **4s**, bearing two ortho chlorine atoms, increased from 27 to 88% under modified reaction conditions. When two electron-donating groups were attached to the phenyl ring as in **4k**, unidentified polymeric material was obtained. Similarly, the presence of the carboxylic acid functional group (**4o**) again resulted in the formation of a polymeric material. See Supporting Information S5 for details on the general fluorination procedures, S6 for fluorination of benzylic substrates, and S12 for spectroscopic data of isolated compounds.

### Substrate Scope Expansion

We wanted to test the fluorination capabilities of **3** on other types of substrates as well (Table 4). Efficient fluorination was achieved on a variety of substrates, albeit most of them required individual optimization of reaction conditions. Successfully fluorinated substrates include a primary iodide, a secondary mesylate, α-bromocarbonyl compounds, a nitroaromatic compound, and sulfonyl and acyl chlorides. Primary iodide **7a** gave 11% of the corresponding elimination side product. The remainder after fluorination of secondary mesylate **7b** is a mixture of alkenes. Fluoride in this reaction system has therefore a non-negligible basic character. In addition to fluorination, reagent **3** can also be used as a mild reagent for the deprotection of silyl ethers. For details, see Supporting Information S7.

**Table 4:**
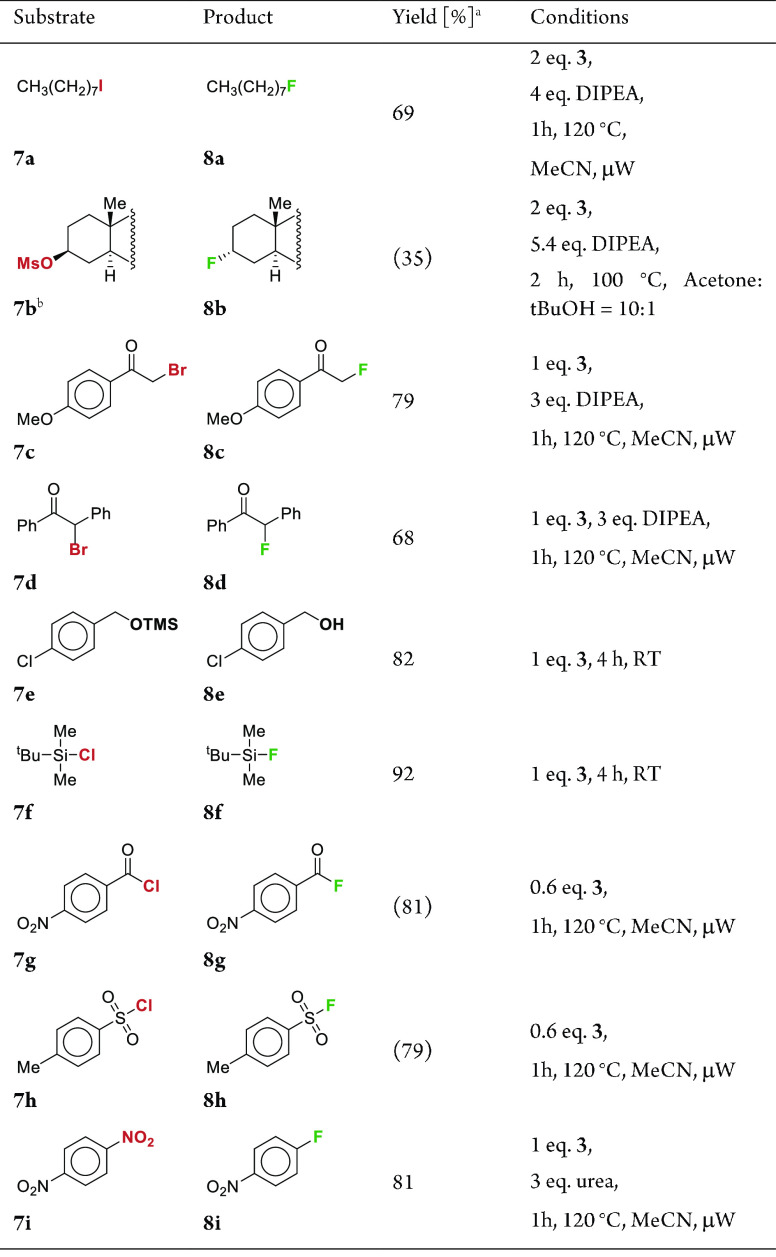
Fluorination of Various Substrates with [IPrH][H_2_F_3_] (**3**)^a^^,^^b^

a Yields were determined by ^1^H NMR integration with naphthalene as an internal standard. Yields in parenthesis are isolated yields.

b **7b** = 5α-cholestan-3β-yl mesylate.

### “Curious Case” of Alkali Fluorides

Some literature cases report the use of an external fluoride source alongside main reagents to achieve higher yields of fluorinated products.^21,22,31,32^ A 2016 research on the deoxyfluorination reaction mechanism demonstrated a fundamental understanding of the role of an external fluoride source in the presence of poly(hydrogen fluoride) anion. Researchers showed that CsF cannot serve as a source of nucleophilic fluoride for that particular fluorination process. Instead, the research makes a strong case that the function of CsF is the abstraction of HF molecule from the bifluoride anion of the final intermediate before the fluorination reaction takes place.^30^

Heavier alkali metal fluorides are known to form more stable poly(hydrogen fluoride) compounds MF(HF)*_n_*^33^ and in this way possibly contribute to the formation of free fluoride anions in a solution.^30^ To expand the observations of the aforementioned past research on the topic, we substituted DIPEA with alkali metal fluorides, MF, and found that the fluorination process with reagent **3** was significantly accelerated using potassium and cesium fluorides. This suggests that LiF and NaF do not have a sufficient propensity to form bifluoride anions to have the same effect as their heavier analogues KF and CsF (Table 5).

**Table 5:** Activation of [IPrH][H_2_F_3_] (**3**) with Alkali Fluorides

#	MF	**4a** [%]^a^	**5a** [%]^a^
1	LiF	31	traces
2	NaF	30	6
3	KF	traces	91
4	CsF	traces	90

a Yields were determined by ^1^H NMR integration with naphthalene as an internal standard. Reactions were performed on a 0.1 mmol scale.

These results with alkali fluorides further substantiate the conclusions made in 2016 and reflect a wider important context of fluorination reactions; they show that poly(hydrogen fluoride) species are essential sources of nucleophilic fluoride in many different reagents. The challenge then remains that how to activate poly(hydrogen fluoride) species to achieve the desired reactivity and how to tune the imidazole part of the reagents for different fluorination processes.^20,21^

### Fluorination Scale-Up

Reaction scale-up was investigated with **4c** due to the low volatility of the corresponding product **5c.** A lower yield (74%, isolated) was initially observed when the reaction scale was increased from 0.5 to 1.5 mmol under microwave conditions. We surmised that higher reagent concentration might be problematic as we were unable to proportionally scale the amount of added solvent to the reaction mixture due to the limited volume of the microwave reactor vial. Fortunately, reducing the reaction time from 1 h to 10 min restored the expected reactivity at higher concentrations (91% isolated yield). This further increases the practicality of the fluorination process in terms of shorter reaction times and lower solvent consumption. For even larger scales (up to 3.7 mmol), conventional heating was used (80 °C, 24 h), which gave an 80% yield (unoptimized). For the reaction scale-up, see Supporting Information, S4.

### Optimized Synthesis of Reagent 3

**Scheme 3:**
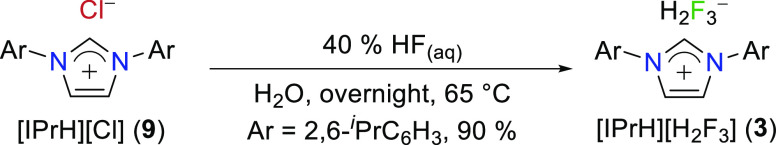
Improved Synthesis of 3 with Hydrofluoric Acid Directly from Air-Stable Imidazolium Chloride 9

From the original study, the synthesis of reagent **3** proceeded *via* the isolation of IPr under anhydrous conditions with the subsequent addition of an anhydrous HF source (KHF_2_, Et_3_N·3HF or aHF).^23^ To avoid the IPr isolation step, a process was introduced that allowed the synthesis of **3** in larger quantities under atmospheric conditions. This process exploited the high solubility of the IPr precursor [IPrH][Cl] (**9**) in water and its atmospheric stability.^34^ Reaction of **9** with 40% hydrofluoric acid affords **3** as the only product on a 10 g scale (Scheme 3).

Through this process, we established a simplified approach to the synthesis of reagent **3**, eliminating the need for anhydrous conditions (for synthesis details, see Supporting Information S8). A good characteristic of this procedure was an exclusive formation of [H_2_F_3_]^−^ anion in spite of excess HF. Larger poly(hydrogen fluoride) species, *e.g.*, [H_3_F_4_]^−^, or inclusion of water was not observed in this system, as was the case in the past.^16,19,35^

### Reaction Workup, Isolation, and Recycling Protocol of 3

**Scheme 4:**
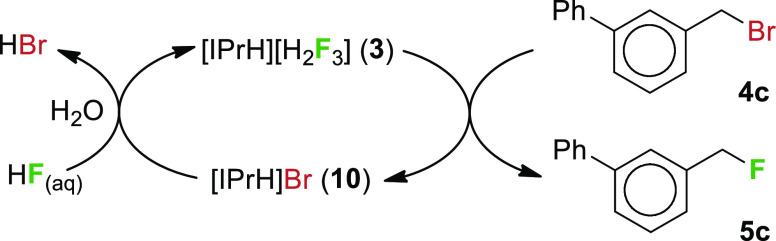
Recycling Protocol for 3 including Recovery of [IPrH][A*_x_*] and Subsequent Regeneration with Aqueous HF

Knowing the specific properties of the imidazolium cation, we took steps toward a sustainable and greener fluorination procedure with a minimum amount of waste. We implemented a workup protocol where we could simultaneously isolate the fluorinated product **5c** and recycle reagent **3** (Scheme 4).

**Scheme 5:**
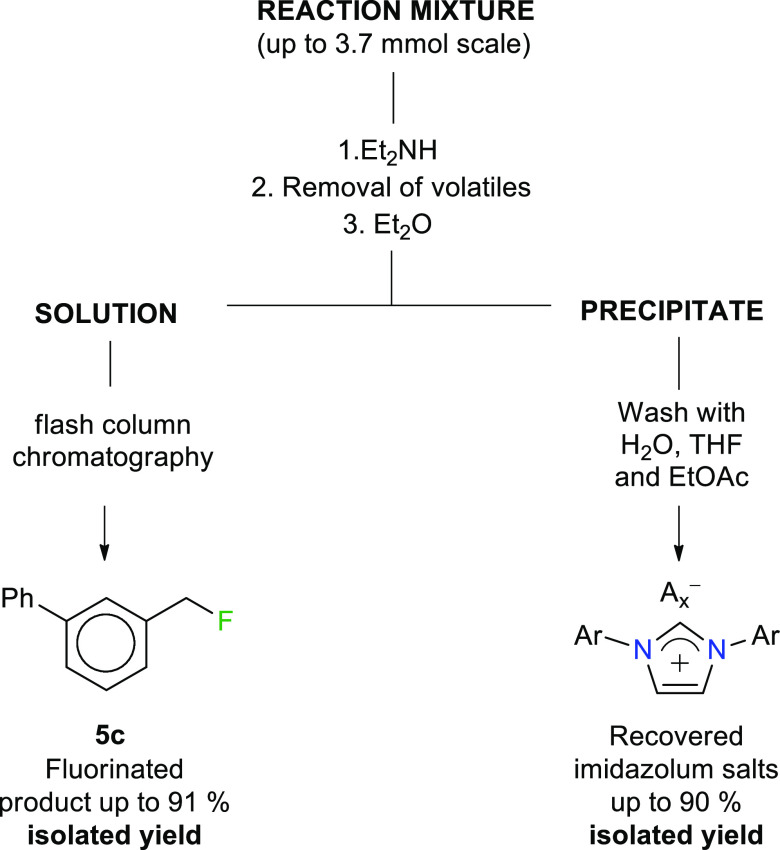
Simplified Separation Scheme

After the reaction, a few drops of Et_2_NH is added to the reaction mixture to facilitate subsequent purification of the fluorinated product **5c**. Et_2_NH reacts preferentially with unreacted starting material **4c**, converting it to easily separable amine. All volatiles are then removed under reduced pressure. Imidazolium salts [IPrH][A*_x_*] precipitate from the residue upon the addition of diethyl ether and can be subsequently filtered off (Scheme 5). [A*_x_*]^−^ of [IPrH][A*_x_*] represents a mixture of predominant Br^–^ anion resulting from the substitution reaction, as well as a small amount of unreacted [H_2_F_3_]^−^ anion, which is also recovered in the imidazolium salt fraction (for details on the isolation and recovery of the imidazolium salt, see Supporting Information S9). Simple washings of the precipitate with water, THF, and ethyl acetate result in spectroscopically pure imidazolium salts with up to 90% isolated yields (Scheme 5). Evaporation of the ethereal filtrate and purification with flash chromatography then give the fluorinated product **5c** (see Supporting Information S9 for isolation procedure).

Recovered imidazolium salts [IPrH][A*_x_*] are then subjected to the regeneration procedure. Scarce literature reports on spent fluorination reagent recycling lack descriptive experimental procedures or they are chemically very strenuous.^12,16,36,37^ Here, we note that the regeneration of [IPrH][A*_x_*] mixture depends on different leaving groups arising from substitution reaction. The initial synthesis of the pure reagent **3** derived from **9** (where [A*_x_*^–^] = Cl^–^) can be achieved simply by consecutive treatments of **9** with hydrofluoric acid (Scheme 3). As can be seen from Table 6, the regeneration of [IPrH][A*_x_*] (*e.g.*, where [A_x_]^−^ = Br^–^, [H_2_F_3_]^−^; Schemes 4 and 5) can also be readily achieved by employing hydrofluoric acid. With this methodology, the fluoride content *w*_F_ in recovered imidazolium salts is increased from 1.6 to 10.3%. However, complete substitution in the Br/H_2_F_3_ system can be accomplished with the use of anhydrous HF (for details of the regeneration procedures, see Supporting Information S9).

**Table 6:** Mass Fractions of Fluoride and Bromide in Samples before and after Regeneration

sample	*w*_F_ [%]	*w*_Br_ [%]
recovered [IPrH][A_*x*_]	1.6	15.4
recycled **3** using hydrofluoric acid	10.3	2.5
recycled **3** using anhydrous HF	12.8	<LOQ
theoretical value for **3**	12.7	0

## Conclusions

The [IPrH][H_2_F_3_] (**3**) reagent shows a good balance between reactivity and stability. The specific combination of bulky imidazolium cation and [H_2_F_3_]^−^ anion of **3** makes it a nonhygroscopic and easy-to-handle fluorination tool. The reagent’s reactivity can be influenced by activators (*e.g.*, DIPEA or alkali metal fluorides), and reaction times can be substantially reduced under microwave irradiation conditions. Reagent **3** and DIPEA were successfully used under microwave conditions for the fluorination of many different types of substrates such as benzylic substrates, α-bromocarbonyls, sulfonyl and acyl chlorides, a nitroaromatic substrate, and primary and secondary aliphatic compounds. Fluorination with **3** is also efficient in substituting a variety of leaving groups such as Br, Cl, I, OMs, OTs, and NO_2_. We developed a convenient procedure for reagent synthesis, eliminating the need for anhydrous reaction conditions. Postfluorination isolation of imidazolium salts, [IPrH][A*_x_*], was achieved with common solvents and standard techniques. This work also demonstrates that the regeneration of [IPrH][A*_x_*] back to reagent **3** is possible using a common and inexpensive 40% hydrofluoric acid. Future work fully explores the potential of imidazole-based fluoride reagents. Research in their fluorination and regeneration procedures may convert **3** from a laboratory curiosity to a reagent of choice for industrial-scale use.

## Supporting Information Available

FDA analysis, detailed experimental procedures for the synthesis and reuse of reagent, procedures for fluorination, crystal structure data, and spectroscopic data (PDF)

## Accession Codes

CCDC 2054235 contains the supplementary crystallographic data for this paper. These data can be obtained free of charge via www.ccdc.cam.ac.uk/data_request/cif, or by emailing data_request@ccdc.cam.ac.uk, or by contacting The Cambridge Crystallographic Data Centre, 12 Union Road, Cambridge CB2 1EZ, UK; fax: +44 1223 336033.
