# Supplementary material for: Renewable Reagent for Nucleophilic Fluorination
Source: J Org Chem. 2022 Apr 19;87(9):5987–93. doi: 10.1021/acs.joc.2c00247 (PMC9087198; doi:10.1021/acs.joc.2c00247)
Supplement: Supplementary file 1 — jo2c00247_si_001.pdf [file jo2c00247_si_001.pdf]

2022

A Renewable Reagent for Nucleophilic Fluorination

Blaž Alič[a‡]*, Jan Petrovčič[b‡], Jan Jelen[a,b], Gašper Tavčar[a]*, Jernej Iskra[b]*

[a]Jožef Stefan Institute, Ljubljana, Slovenia,
[b]University of Ljubljana, Faculty of Chemistry and Chemical Technology, Ljubljana, Slovenia

Contents

S1 FDA analysis ***S4***

S2 Materials and Methods ***S11***

S3 Reactivity of reagents 1, 2, and 3 ***S12***

Comparison of imidazolium poly(hydrogen fluoride) reagents **1**, **2**, and **3** S12

S4 Parameter optimization for fluorination reactions with reagent 3 ***S13***

Activation of reagent 3 S13

Ratio optimization S14

Reaction scale-up S15

S5 General procedures for fluorination (A, B and C) ***S16***

Procedure A S16

Procedure B S16

Procedure C S17

S6 Fluorination of benzylic substrates ***S18***

1-(*tert*-butyl)-4-(fluoromethyl)benzene (**5a**) S18

1-(fluoromethyl)-4-methylbenzene (**5b**) S18

3-(fluoromethyl)-1,1'-biphenyl (**5c**) S19

1-bromo-4-(fluoromethyl)benzene (**5d**) S19

1-bromo-3-(fluoromethyl)benzene (**5e**) S19

1-fluoro-4-(fluoromethyl)benzene (**5f**) S19

Ethyl 4-(fluoromethyl)benzoate (**5g**) S20

1-(fluoromethyl)-3,5-bis(trifluoromethyl)benzene (**5h**) S20

1-(fluoromethyl)-4-nitrobenzene (**5i**) S20

1-(fluoromethyl)-3-methoxybenzene (**5j**) S21

1-chloro-4-(fluoromethyl)- benzene (**5m**) S21

1,3-dichloro-2-(fluoromethy)benzene (**5n**) S21

1-(fluoromethyl)-4-methoxybenzene (**5q**) S21

Fluoromethylbenzene (**5t**) S21

S7 Fluorination of other substrates ***S22***

1-fluorooctane (**8a**) S22

Oct-1-ene (side product in fluorination of 8a) S22

3α-fluoro-5α-cholestane (**8b**) S22

5α-cholest-3-ene and 5α-cholest-2-ene[34,35] (side products in fluorination of **8b**) S23

2-fluoro-1-(4-methoxyphenyl)ethan-1-one (**8c**) S23

2-fluoro-1,2-diphenylethan-1-one (**8d**) S23

(4-chlorophenyl)methanol (**8e**) S23

Tert-butyldimethylsilyl fluoride (**8f**) S23

4-nitrobenzoyl fluoride (**8g**) S24

4-methylbenzenesulfonyl fluoride (**8h**) S24

1-fluoro-4-nitrobenzene (**8i**) S25

***S8 Synthesis of reagent [IPrH][H_2F_3] (3) under ambient conditions* S26**

Influence of molar ratios between **9** and HF and details on synthesis optimization. S26

***S9 Post-fluorination recovery of reagent 3* S28**

Isolation procedure for fluorinated product and imidazolium salts S28

Regeneration procedure of imidazolium salt [IPrH][A_x] to reagent **3** S31

a) Regeneration of **3** with hydrofluoric acid S31

b) Regeneration of **3** with anhydrous HF S33

***S10 Synthesis and characterization of 6* S35**

Single Crystal XRD analysis of compound of **6** S36

***S11 Synthesis and characterization of other products (14 - 17)* S41**

(**14**) 1-(4-(*tert*-butyl)benzyl)pyridinium bromide S41

(**15**) (4-(*tert*-butyl)benzyl)triethylammonium bromide S42

(**16**) (4-(*tert*-butyl)benzyl)ethyldiisopropylammonium bromide S42

(**17**) (3-(pheny)lbenzyl)ethyldiisopropylammonium bromide S43

***S12 Spectroscopic data of isolated compounds* S44**

Spectroscopic data of 1-(tert-butyl)-4-(fluoromethyl)benzene (**5a**) S44

Spectroscopic data of 1-(fluoromethyl)-4-methylbenzene (**5b**) S45

Spectroscopic data of 3-(fluoromethyl)-1,1'-biphenyl (**5c**) S46

Spectroscopic data of 1-bromo-4-(fluoromethyl)benzene (**5d**) S47

Spectroscopic data of ethyl (4-fluoromethyl)benzoate (**5g**) S48

Spectroscopic data of 1-(fluoromethyl)-3,5-bis(trifluoromethyl)-benzene (**5h**) S49

Spectroscopic data of 1-(fluoromethyl)-4-nitrobenzene (**5i**) S51

Spectroscopic data of 3α-fluoro-5α-cholestane (**8b**) S52

Spectroscopic data of 4-nitrobenzoyl fluoride (**8g**) S53

Spectroscopic data of 4-methylbenzenesulfonyl fluoride (**8h**) S54

Spectroscopic data of 2-(4-(tert-butyl)benzyl)-1,3-bis(2,6-diisopropylphenyl)-1H-imidazol-3-ium bromide (**6**) S55

Spectroscopic data of 1-(4-(tert-butyl)benzyl)pyridium-bromide (**14**) S57

Spectroscopic data of (4-(tert-butyl)benzyl)triethyl-ammonium bromide (**15**) S59

Spectroscopic data of (4-(tert-butyl)benzyl)ethyldiisopropyl-ammonium bromide (**16**) S61

Spectroscopic data of (3-(phenyl)benzyl)ethyldiisopropyl-ammonium bromide (**17**) S63

***S13 References* S65**

S1 FDA analysis

In the following tables, please find yearly analyses of Food and Drug Administration (FDA) approved drugs for a given year in a timespan from 2008 to 2019. Each table cell represents one drug with its active pharmaceutical ingredient(s) (APIs). APIs highlighted in green contain at least one fluoride substituent in their structure.

2008

List of active ingredients in FDA approved drugs in 2008 (fluorinated active ingredients are printed in bold and colored green)[1].

rilonacept	regadenoson	tetrabenazine	eltrombopag
certolizumab pegol	methylnaltrexone bromide	iobenguanei-123	tapentadol hydrochloride
romiplostim	alvimopan	silodosin	fospropofoldisodium
etravirine	difluprednate	lacosamide	plerixafo
desvenlafaxine	gadoxetate disodium	fesoterodine	gadofosveset
bendamustine hydrochloride	clevidipine butyrate	rufinamide	degarelix

3 out of 24 (12.5 %) approved drugs in 2008 contain fluorinated active ingredients.

2009

List of active ingredients in FDA approved drugs in 2009 (fluorinated active ingredients are printed in bold and colored green)[2].

golimumab	milnacipran	tolvaptan	asenapine
abobotulinum-toxin A	febuxostat	besifloxacin	vigabatrin
canakinumab	everolimus	dronedarone	bepotastine besilate
ustekinumab	artemether–lumefantrine	prasugrel	telavancin
ofatumumab	benzyl alcohol	saxagliptin	pralatrexate
ecallantide	iloperidone	pitavastatin	pazopanib HCl
romidepsin		extavia	

4 out of 26 (15.4 %) approved drugs in 2009 contain fluorinated active ingredients.

2010

List of active ingredients in FDA approved drugs in 2010 (fluorinated active ingredients are printed in bold and colored green)[3].

dalfampridine	oestradiol valerate, dienogest	dabigatran	tocilizumab
liraglutide	cabazitaxel	lurasidone	collagenase clostridium histolyticum
velaglucerase alfa	alcaftadine	ceftaroline fosamil	alglucosidase alfa
carglumic acid	ulipristal	tesamorelin	denosumab
polidocanol	fingolimod	eribulin	incobotulinumtoxinA
pegloticase			

0 out of 21 (0.0 %) approved drugs in 2010 contain fluorinated active ingredients.

2011

List of active ingredients in FDA approved drugs in 2011 (fluorinated active ingredients are printed in bold and colored green)[4].

aflibercept	brentuximab vedotin	fidaxomicin	gabapentin enacarbil
asparaginase Erwinia chrysanthemi	**vemurafenib**	telaprevir	ipilimumab
ruxolitinib	**ticagrelor**	rilpivirine	gadobutrol
clobazam	rivaroxaban	boceprevir	belimumab
deferiprone	indacaterol	linagliptin	**roflumilast**
**crizotinib**	belatacept	abiraterone acetate	azilsartan medoxomil
icatibant	**ezogabine**	**vandetanib**	vilazodone hydrochloride
spinosad		**ioflupane i-123**	

7 out of 30 (23.3 %) approved drugs in 2011 contain fluorinated active ingredients.

2012

List of active ingredients in FDA approved drugs in 2012 (fluorinated active ingredients are printed in bold and colored green)[5].

crofelemer	omacetaxine mepesuccinate	**elvitegravir,** cobicistat, **emtricitabine,** tenofovir disoproxil fumarate	**florbetapir F-18**
bedaquiline	perampanel	ziv-aflibercept	peginesatide
apixaban	ocriplasmin	aclidinium bromide	lucinactant
lomitapide	**regorafenib**	carfilzomib	**tafluprost**
teduglutide	choline-C11	sodium picosulfate, magnesium oxide and citric acid	ivacaftor
pasereotide	**teriflunomide**	mirabegron	vismodegib
raxibacumab	bosutinib	lorcaserin hydrochloride	axitinib
**ponatinib**	**enzalutamide**	pertuzumab	ingenol mebutate
**cabozantinib**	linaclotide	taliglucerase alfa	glucarpidase
tofacitinib	filgrastim	avanafil	

8 out of 39 (20.5 %) approved drugs in 2012 contain fluorinated active ingredients.

2013

List of active ingredients in FDA approved drugs in 2013 (fluorinated active ingredients are printed in bold and colored green)[6].

umeclidinium, vilanterol	**flutemetamol F-18**	**trametinib**	Tc-99m tilmanocept
**sofosbuvir**	macitentan	**dabrafenib**	ospemifene
simeprevir	**riociguat**	Ra-223 dichloride	trastuzumab, emtansine
luliconozole	conjugated estrogens, bazedoxifene	**fluticasone furoate,** vilanterol	pomalidomide
ibrutinib	vortioxetine	**canagliflozin**	mipomersen sodium
eslicarbazepine acetate	**dolutegravir**	dimethyl fumarate	alogliptin
obinutuzumab	**afatinib**	gadoterate, meglumine	

9 out of 27 (33.3 %) approved drugs in 2013 contain fluorinated active ingredients.

2014

List of active ingredients in FDA approved drugs in 2014 (fluorinated active ingredients are printed in bold and colored green)[7].

nivolumab	netupitant, palonosetron	olodaterol	siltuximab
peramivir	ledipasvir, sofosbuvir	idelalisib	ramucirumab
ceftolozane, tazobactam	dulaglutide	tavaborole	albiglutide
ombitasvir, paritaprevir, ritonavir, dasabuvir	naloxegol	belinostat	apremilast
olaparib	pembrolizumab	tedizolid phosphate	miltefosine
finafloxacin	eliglustat	efinaconazole	florbetaben F-18
blinatumomab	peginterferon beta-1A	dalbavancin	metreleptin for injection
pirfenidone	suvorexant	vedolizumab	droxidopa
nintedanib	oritavancin	vorapaxar	elosulfase alfa
sulfur hexafluoride lipid microsphere	empagliflozin	ceritinib	tasimelteon
dapaglifozin			

10 out of 41 (24.4 %) approved drugs in 2014 contain fluorinated active ingredients.

2015

List of active ingredients in FDA approved drugs in 2015 (fluorinated active ingredients are printed in bold and colored green)[8].

lesinurad	elvitegravir, cobicistat, emtricitabine, tenofovir alafenamide	rolapitant	deoxycholic acid
selexipag	mepolizumab	evolocumab	ivabradine
sugammadex	asfotase alfa	flibanserin	cholic acid
alectinib	trabectedin	daclatasvir	dinutuximab
sebelipase alfa	patiromer	sonidegib	isavuconazonium sulfate
elotuzumab	idarucizumab	alirocumab	ceftazidime-avibactam
necitumumab	aripiprazole lauroxil	brexpiprazole	panobinostat
ixazomib	Insulin, degludec	sacubitril/valsartan	lenvatinib
daratumumab	trifluridine, tipiracil	lumacaftor, ivacaftor	palbociclib
osimertinib	cariprazine	cangrelor	parathyroid horomone
cobimetinib	uridine triacetate	eluxadoline	secukinumab
edoxaban			

10 out of 45 (22.2 %) approved drugs in 2015 contain fluorinated active ingredients.

2016

List of active ingredients in FDA approved drugs in 2016 (fluorinated active ingredients are printed in bold and colored green)[9].

nusinersen	eteplirsen	**fluciclovine F-18**	venetoclax
**rucaparib**	lixisenatide	obeticholic acid	defibrotide sodium
crisaborole	lifitegrast	daclizumab	reslizumab
bezlotoxumab	**sofosbuvir,** velpatasvir	atezolizumab	ixekizumab
olaratumab	Ga-68 dotatate	**pimavanserin**	obiltoxaximab
brivaracetam		elbasvir, grazoprevir	

4 out of 22 (18.2 %) approved drugs in 2016 contain fluorinated active ingredients.

2017

List of active ingredients in FDA approved drugs in 2017 (fluorinated active ingredients are printed in bold and colored green)[10].

angiotensin II	acalabrutinib	guselkumab	deutetrabenazine
macimorelin acetate	**abemaciclib**	betrixaban	ocrelizumab
ertugliflozin	secnidazole	**delafloxacin**	dupilumab
netarsudil	copanlisib	sarilumab	niraparib
ozenoxacin	benznidazole	edaravone	naldemedine
semaglutide	meropenem and vaborbactam	durvalumab	avelumab
emicizumab	inotuzumab ozogamicin	abaloparatide	**safinamide**
vestronidase alfa	**glecaprevir, pibrentasvir**	midostaurin	ribociclib
benralizumab	**enasidenib**	brigatinib	**telotristat ethyl**
**letermovir**	**sofosbuvir,** velpatasvir**, voxilaprevir**	cerliponase alfa	brodalumab
latanoprostene bunod	neratinib maleate	valbenazine	deflazacort
etelcalcetide		plecanatide	

8 out of 46 (17.4 %) approved drugs in 2017 contain fluorinated active ingredients.

2018

List of active ingredients in FDA approved drugs in 2018 (fluorinated active ingredients are printed in bold and colored green)[11].

ravulizumab	elapegademase	segesterone acetate, ethinyl estradiol	pegvaliase
tagraxofusp	omadacycline	patisiran	avatrombopag
calaspargase pegol	sarecycline	mogamulizumab	sodium zirconium cyclosilicate
prucalopride	cemiplimab	lusutrombopag	erenumab
gilteritinib	dacomitinib	fish oil triglycerides	lofexidine hydrochloride
amifampridine	galcanezumab	elagolix sodium	**fosnetupitant,** palonosetron
**larotrectinib**	duvelisib	**tafenoquine**	burosumab
glasdegib	fremanezumab	**ivosidenib**	**fostamatinib**
emapalumab	moxetumomab pasudotox	**tecovirimat**	tildrakizumab
rifamycin	**doravirine**	**encorafenib**	ibalizumab
revefenacin	**eravacycline**	**binimetinib**	**apalutamide**
**lorlatinib**	lanadelumab	plazomicin	**tezacaftor,** ivacaftor
**baloxavir marboxil**	cenegermin	cannabidiol	**bictegravir, embitcitabine,** tenofovir alafenamide
**talazoparib**	stiripentol	moxidectin	Lu-177 dotatate
inotersen	migalastat	baricitinib	

16 out of 59 (27.1 %) approved drugs in 2018 contain fluorinated active ingredients.

2019

List of active ingredients in FDA approved drugs in 2019 (fluorinated active ingredients are printed in bold and colored green)[12].

**ubrogepant**	zanubrutinib	lefamulin	polatuzumab vedotin
trastuzumab **deruxtecan**	luspatercept	**upadacitinib**	**alpelisib**
**lemborexant**	air polymer-type A	fedratinib	tafamidis
**lumateperone tosylate**	**elexacaftor,** ivacaftor, **tezacaftor**	**entrectinib**	risankizumab
Brilliant Blue G	**lasmiditan**	pitolisant	erdafitinib
enfortumab vedotin	**fluorodopa F-18**	**pretomanid**	romosozumab
golodirsen	afamelanotide	**pexidartinib**	**siponimod**
voxelotor	brolucizumab	darolutamide	solriamfetol
cenobamate	trifarotene	ferric maltol	brexanolone
**givosiran**	tenapanor	imipenem, cilastatin, relebactam	triclabendazole
crizanlizumab	istradefylline	selinexor	caplacizumab
cefiderocol	Ga-68-Dotatoc	bremelanotide	prabotulinum-toxinA

14 out of 48 (29.2 %) approved drugs in 2019 contain fluorinated active ingredients.

S2 Materials and Methods

All compounds and solvents used are commercial products from Merck, Sigma-Aldrich, Honeywell, Eurisotop, Fluka etc. and were used without further purification unless otherwise noted. 1,3-bis(2,6-diisopropylphenyl)imidazol-2-ylidene (IPr) and its precursor 1,3-bis(2,6-diisopropylphenyl)imidazolium chloride were prepared according to the standard literature procedures[13,14]. Cholestanol was prepared according to the literature[15]. 5α-choletan-3β-yl mesylate (**7b**) was prepared according to the literature[16]. 2-bromo-1-(4-methoxyphenyl)ethan-1-one (**7c**) was prepared according to literature[17]. ((4-chlorobenzyl)oxy)trimethylsilane (**7e**) was prepared according to a modified procedure from the literature[18] (TMSCl was used instead of TBDMSCl). *tert*-butanol was stored over 3 Å molecular sieves, which were activated in an oven at 250 °C for 10 hours. Anhydrous acetonitrile was either prepared according to literature[19] or stored over activated 3 Å molecular sieves. All NMR scale and preparative fluorination reactions were carried out in glassware. Reagent synthesis, isolation and regeneration were carried out in plastic beakers or FEP reactors.

Reactions were monitored with NMR, GC and TLC. TLC spots were visualized under UV light (254 nm UV indicator) or with $KMnO_4$ TLC stain.

^{1}H, ^{13}C and ^{19}F NMR spectra were recorded with Agilent Technologies Unity Inova 300 MHz spectrometer at 25 °C, Bruker Avance DPX 300 MHz spectrometer at 29 °C and Bruker Avance III 500 MHz spectrometer at 23 °C. ^{1}H NMR chemical shifts are reported in ppm relative to TMS (internal standard). ^{13}C NMR chemical shifts are reported in ppm relative to residual solvent peak ($CDCl_3$, CD_3CN). ^{19}F NMR chemical shifts are reported in ppm relative to $CFCl_3$ (internal standard).

Ion chromatography was performed using Dionex ICS-5000 ion chromatograph with AS-5000 autosampler, gradient pump and conductivity detector. Two different columns were used: Dionex IonPac® AS14 and Dionex IonPac® AS11-HC (4 x 250 mm). Isocratic elution was employed with NaOH used as an eluent. Fluoride, chloride and bromide calibration curves were prepared by dilution of multistandard stock solution of NaF, NaCl and NaBr in MiliQ water.

HRMS spectrum was recorded with Micromass Waters Q-TOF Premier high resolution mass spectrometer (electrospray ionization ESI) coupled with LC.

Single-crystal data of **6** was collected on a Gemini A diffractometer equipped with an Atlas CCD detector, using graphite monochromated Cu Kα radiation.

GC analyses were performed with Hewlett Packard 6890 gas chromatograph coupled with mass spectrometer.

Elemental analyses were obtained with Elementar Vario EL CUBE elemental analyzer.

Caution. Working with anhydrous hydrogen fluoride/hydrofluoric acid is hazardous, so it must be handled in a well-ventilated fume hood and personal protective equipment must be worn at all times. The experimenter must become familiar with these reagents and the hazards associated with them. Fresh tubes of calcium gluconate gel should always be on hand for immediate treatment of skin that has come in contact with these reagents. A complete protocol for the treatment of HF exposure, see (Peters, D.; Miethchen, R. J. Fluorine Chem. 1996, 79, 161-165.). Excess calcium hydroxide is used to neutralize waste hydrofluoric acid.

S3 Reactivity of reagents **1**, **2**, and **3**

Comparison of imidazolium poly(hydrogen fluoride) reagents **1**, **2**, and **3**

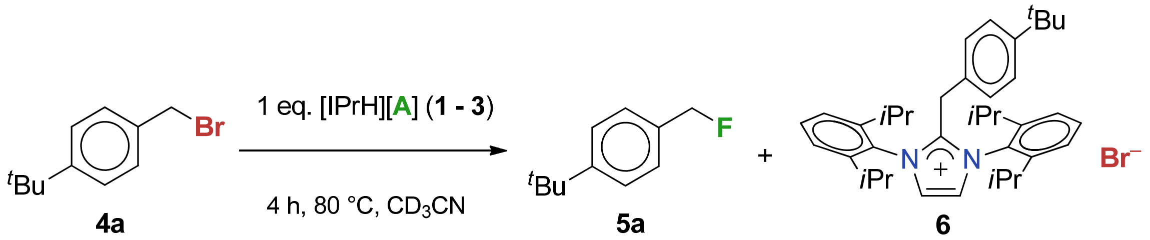

Scheme S3 1: Fluorination with reagents 1, 2 and 3

Under anhydrous conditions, 20 µL (0.1 mmol) of **4a** and 1 equivalent of [IPrH][A] were dissolved in 0.7 ml of CD_3CN. Reaction mixture was heated at 80 °C for 4 hours. ^1H and ^{19}F NMR spectra of reaction mixtures were recorded. Product distribution was determined by ^1H NMR spectra integration (Table S3 1).

Table S3 1: Fluorination with reagents 1, 2 and 3

Reagent	[A]⁻	Conversion [%]	5a [%]	6 [%]
1	$[F]^-$	100	8	92
2	$[HF_2]^-$	45	39	6
3	$[H_2F_3]^-$	18	18	/

The yield of the side product **6** decreases with the increasing number *n* of HF molecules in the $[F(HF)_n]^-$ anion together with the overall conversion. **6** was formed only with reagents **1** and **2** while fluorination product **5a** was observed with all three reagents. The fluorination with reagent **1** affords only minute amounts of fluorinated product **5a** (8 %, entry 1) because of the competition reaction with a better nucleophile, namely, between *in-situ* formed free N-heterocyclic carbene, and a benzylic substrate **4a**. This observation is consistent with the equilibrium between **1**, NHC and **2** in acetonitrile (See Scheme 2 of the manuscript and ref. 23 therein = reference 41 of this document).

Yield of fluorinated product **5a** is the greatest when pure reagent **2** was used. This result is in agreement with the aforementioned equilibrium of **1**, NHC and **2** in acetonitrile and indicates that NHC reaction channel is constrained by an additional HF molecule in the anion. Furthermore, $[HF_2]^-$ anion of **2** can disproportionate to F^- and $[H_2F_3]^-$, forming an active F^- nucleophile and more deactivated/stable $[H_2F_3]^-$ species. This observation explains lowered overall conversion (entry 2).

Following this trend, the reactivity of **3** is the lowest, with overall 19% conversion, indicating that both NHC and F^- nucleophiles are constrained by two additional HF molecules in the anion (entry 3).

For synthesis and characterization of reagent **3** under ambient conditions please refer to Section S8 of this document.

For synthesis and characterization of product **6** please refer to Section S10 of this document.

S4 Parameter optimization for fluorination reactions with reagent **3**

Activation of reagent 3

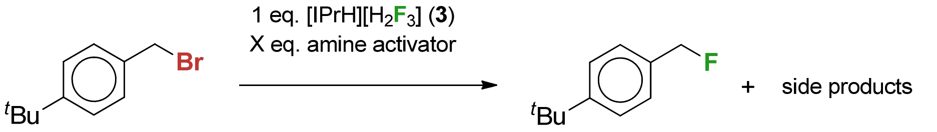

4a **5a** **11 or 12 or 13**

Scheme S4 1: Standard test for amine activators

0.1 mmol of **4a** (M = 227.14 g mol^{-1}) was weighed in a vial and dissolved in 0.5 ml of acetonitrile. Reagent **3** and corresponding amine activator were added. Naphthalene was weighed in a separate vial (internal standard; 0.5 equiv.) and dissolved in 0.5 ml of acetonitrile. Obtained solutions in both vials were thoroughly mixed. Reaction mixture was transferred into a microwave reactor vial and then heated to 120 °C under microwave irradiation for 1 hour. After the reaction, an aliquot of reaction mixture was diluted with $CDCl_3$ and ^{1}H NMR spectrum was recorded. Product distribution was determined by comparing integrals of internal standard and product peaks (Table S4 1).

Table S4 1: Formation of side products for different amine activators

Amine activator	X	Conversion [%]	**5a** [%]	Side product structure	Side product [%]
Pyridine	1	94	31	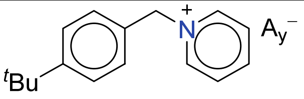 **11**	> 43[a]
Et_3N	3	100	32	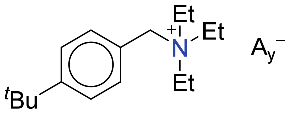 **12**	68
DIPEA	3	98	98	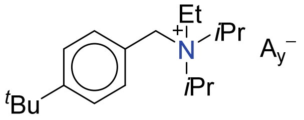 **13**	traces[b]

[a]Side product partially precipitated out of the reaction mixture. [b]Traces were observed only in a few reactions.

The reported additives/activators from the literature (*e.g.* pyridine and Et_3N) are incompatible with this reaction system. They act as competitive nucleophiles for the benzylic position to form side products **11** and **12** (Table S4 1). The structure of the amine salt formation could easily be recognized from the NMR analysis of the reaction mixture. We note, that the counter ion with a general label $[A_y]^-$ could be a number of different species, which we did not pursue to identify. The amount of side product **11** was over 40% with one equivalent of pyridine added to the reaction mixture (entry 1). Et_3N also favors reaction with benzylic substrate, forming side product **12** in 68% yield (entry 2). Therefore, the reaction system with benzylic substrates requires an additive that has reduced affinity for the substrate. DIPEA enables formation of the fluorinated product in excellent yields (> 90%) with no or negligible amounts of the side product **13** (via NMR or GCMS, entry 3). Because the amount of this side product was non-existent or found only in traces, and it could be easily removed from the reaction mixture with H_2O, we used DIPEA as the activator of choice.

For direct reactions between benzylic substrates and activators see S11.

Ratio optimization

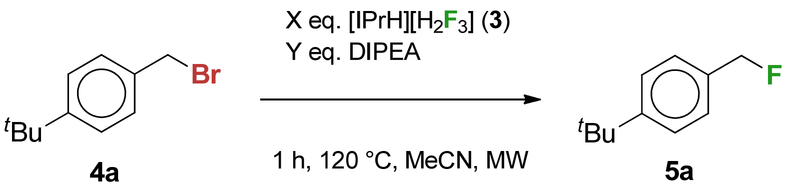

*Scheme S4 2: Optimization of stoichiometry of added reagetnt **3** and DIPEA*

0.1 mmol of **4a** (M = 227.14 g mol^{-1}) was weighed in a vial and dissolved in 0.5 ml of acetonitrile. Amount X of reagent **3** and amount Y of DIPEA were added to the obtained solution. Naphthalene was weighed in a separate vial (internal standard; 0.5 equiv.) and dissolved in 0.5 ml of acetonitrile. Obtained solutions in both vials were thoroughly mixed. Reaction mixture was transferred into a microwave reactor vial and then heated to 120 °C under microwave irradiation for 1 hour. After the reaction an aliquot of reaction mixture was diluted with $CDCl_3$ and ^1H NMR spectrum was recorded. Reaction conversions and yields were determined by comparing integrals of internal standard and product peaks (Table S4 2). The process was iterated to obtain quantitative conversion and yields of fluorinated product.

Table S4 2: Conversions and fluorination yields with varying ratio of reagent 3 and DIPEA

Entry	X eq. [IPrH][H_2F_3]	Y eq. DIPEA	Conversion [%]	5a [%]
1	0.5	0	33	28
2	1	0	48	23
3	0.5	0.5	78	74
4	0.5	1.0	77	75
5	0.5	1.7	92	87
6	0.5	3.2	97	86
7 (optimized)	0.6	1.8	96	95

The addition of DIPEA increases the yield of fluorination from 28% to 74% (entries 1 and 3). We fluorinated **4a** with 0.5 equivalents of the reagent (this amounts to 1.5 equivalents of fluoride in the reaction). Under these reaction conditions, we could only obtain yields up to 87 %, regardless of the amount of DIPEA present in the system (entries 3 – 6). To achieve excellent yields (> 90 %) the quantity of the **3** had to be raised to 0.6 equivalents (1.8 equivalents of fluoride) and DIPEA to 1.8 equivalents (entry 7).

Reaction scale-up

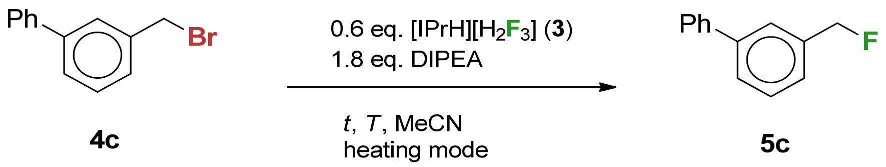

Scheme S4 3: Optimization of other reaction parameters

Specified amount of substrate **4c**, 0.6 equivalents of **3** and 1.8 equivalents of DIPEA were dissolved in the appropriate volume of acetonitrile (Table S4 3). Reaction mixture was heated at fixed temperature for the determined amount of time. Either glass or FEP reaction vessel was used. After the reaction, acetonitrile was removed under reduced pressure. 5 ml of diethyl ether was added to the obtained oily residue causing imidazolium salts to precipitate. Imidazolium salts were then filtered off and subsequently washed with three additional portions of diethyl ether. Ethereal solutions were combined and dried under reduced pressure. Crude product was purified with flash column chromatography (mobile phase = DCM : mixture of hexanes = 1 : 5) to yield **5c** as a colorless liquid.

Table S4 3: Conversions and fluorination yields with varying stoichiometry of reagent 3 and DIPEA

Entry	Amount of 4c [mmol]	c [M]	Heating mode	*T* [°C]	*t* [h]	5c [%]*
1	0.5	0.5	MW	120	1	90
2	1.1	0.75	MW	120	1	74
3	1.1	0.75	MW	120	0.17	91
4	1.1	0.14	Conventional	80	24	80
5	1.1	0.37	Conventional	80	24	91
6	3.7	0.37	Conventional	80	24	80

* Isolated yields

We wanted to test whether fluorination of benzylic substrates with **3** and DIPEA proceeds any differently when conducted on larger scales (Table S4 3). On our first reaction scale-up attempt, fluorination yield of **5c** decreased from 90 % to 74 % (entries 1 and 2). We could not proportionally scale up the solvent volume due to limitations connected with the size of our microwave reactor vessel. If we wanted to retain high yields of the fluorinated product **5c** using microwave heating at higher concentrations, we had to decrease reaction time to 10 minutes (compare entries 2 and 3). Longer reaction times under these reaction conditions resulted in a more complicated mixture of products and subsequently decreased yield of **5c**.

When we were interested only in the fluorination product, the reactions could easily be performed in glassware without any observable glass etching. However, when we began to investigate the possibility of post-fluorination isolation of imidazole salts and their subsequent recycling back to the reagent **3**, it became apparent from ^{19}F NMR spectra that traces of $[BF_4]^-$ and $[SiF_6]^{2-}$ anions formed during the reactions. To prevent formation of these species all additional experiments associated with reagent recovery and regeneration were performed in FEP reactor vessels (see Section S9).

S5 General procedures for fluorination (A, B and C)

Fluorination reactions on substrates were generally carried out by two procedures, depending on the volatility of the substrate. In procedure A, the reactions were carried out on a 0.1 mmol scale with added internal standard and yields determined by NMR spectroscopy. Alternatively, procedure B was carried out on a preparative scale (0.5 mmol). The products were isolated and yields determined based on the obtained mass of the final, purified product. Procedure C was used to explore possibility of substituting DIPEA activator with alkali metal fluorides. Procedure C was only used on a modal substrate (**4a**) to show the proof of concept that alternative activators can be used, and was not systematically explored on a variety of substrates.

For products **7b**, **7g** and **7h** synthesis was carried out with deviations to general procedures and is specified for each product separately (see Section S7).

Procedure A

NMR yield determination with an internal standard

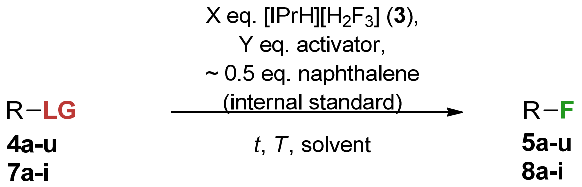

Scheme S5 1: General synthesis procedure A

0.1 mmol of a substrate was weighed in a vial and dissolved in 0.5 ml of acetonitrile. Reagent **3** and activator were added. Naphthalene was weighed in a separate vial (internal standard; 0.5 equiv.) and dissolved in 0.5 ml of acetonitrile. Obtained solutions in both vials were thoroughly mixed. Reaction mixture was transferred into a microwave reactor vial and then heated to 120 °C under microwave irradiation.

Specific reaction condition for fluorination of **7a – i** can be found in manuscript Table 4. After completion, an aliquot of reaction mixture was diluted with $CDCl_3$ and ^1H NMR spectra was recorded. Product yields were determined by comparing integrals of internal standard and product peaks (Table 4 in Manuscript). Products identity was additionally confirmed using GCMS.

Procedure B

Fluorination of benzyl bromides on preparative scale

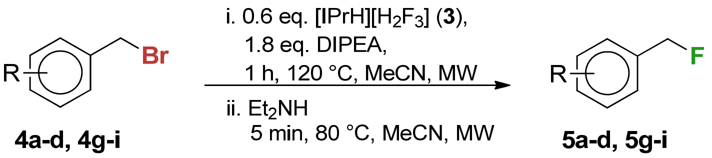

Scheme S5 2: General synthesis procedure B

0.5 mmol of benzylic substrate was dissolved in 1 ml of acetonitrile. 0.6 equivalents of **3** and 1.8 equiv. of DIPEA were added. Reaction mixture was transferred into a microwave reactor vial using an additional 1 ml of acetonitrile. Reactions were carried out under microwave irradiation at 120 °C for 1 h. After the reaction, 5 drops of diethylamine were added and reaction mixture was reheated to 80 °C for 5 minutes. Acetonitrile was removed under reduced pressure. A few milliliters of mobile phase was poured over the obtained residue causing imidazolium salts to precipitate. Resulting solution of crude

fluorinated product was purified using flash column chromatography to yield corresponding benzyl fluoride (72 – 93 % isolated yields, Table 3 of Manuscript).

Procedure C

Fluorination with activation with alkali fluorides

0.1 mmol of a substrate **4a** was weighed in a vial and dissolved in 0.5 ml of acetonitrile and additional 50 μL of H_2O was added. Reagent **3** (0.6 equiv.) and 1.8 equiv. of alkali fluoride (LiF, NaF, KF or CsF) was added. Naphthalene was weighed in a separate vial (internal standard; 0.5 equiv.) and dissolved in 0.5 ml of acetonitrile. Obtained solutions in both vials were thoroughly mixed. Reaction mixture was transferred into a microwave reactor vial and then heated to 120 °C under microwave irradiation. After completion, an aliquot of reaction mixture was diluted with $CDCl_3$ and ^1H NMR spectra was recorded. Product yields were determined by comparing integrals of internal standard and product peaks (Table 5 in Manuscript).

S6 Fluorination of benzylic substrates

1-(*tert*-butyl)-4-(fluoromethyl)benzene (5a)

The title compound was prepared according to the general Procedure B as a colorless oil (78 mg, 93 %). Flash column chromatography was preformed using an isocratic elution with *n*-pentane. Its spectroscopic data is consistent with the previously reported[20]. Spectroscopic data of isolated **5a** can be found on Page 44.

^{1}H NMR (23 °C, $CDCl_3$, 500.13 MHz): δ = 7.42 (dd, *J* = 8 Hz, 1 Hz, 2H), 7.32 (dd, *J* = 8 Hz, 2 Hz, 2H), 5.34 (d, *J* = 48 Hz, 2H), 1.33 (s, 9H).

^{13}C NMR (23 °C, $CDCl_3$, 125.76 MHz): δ = 152.1 (d, *J* = 3 Hz), 133.3 (d, *J* = 17 Hz), 127.8 (d, *J* = 5 Hz), 125.7 (d, *J* = 2 Hz), 84.7 (d, *J* = 165 Hz), 34.8 (s), 31.4 (s).

^{19}F NMR (23 °C, $CDCl_3$, 470.55 MHz): δ = –204.9 (t, *J* = 48 Hz).

MS: 166 (M^+, 21 %), 151 ($[M – CH_3]^+$, 100 %), 123 (33 %), 91 (18 %), 41 (11 %)

1-(fluoromethyl)-4-methylbenzene (5b)

The title compound was prepared according to the general Procedure B as a colorless oil (45 mg, 72 %). Flash column chromatography was preformed using an isocratic elution with *n*-pentane. Its spectroscopic data is consistent with the previously reported[21]. Spectroscopic data of isolated **5b** can be found on Page 45.

^{1}H NMR (23 °C, $CDCl_3$, 500.13 MHz): δ = 7.28 (dd, *J* = 8 Hz, 2 Hz, 2H), 7.20 (d, *J* = 8 Hz, 2H), 5.33 (d, *J* = 48 Hz, 2H), 2.37 (d, *J* = 2 Hz, 3H).

^{13}C NMR (23 °C, $CDCl_3$, 125.76 MHz): δ = 138.9 (d, *J* = 3 Hz), 133.3 (d, *J* = 17 Hz), 129.4 (d, *J* = 2 Hz), 128.0 (d, *J* = 5 Hz), 84.6 (d, *J* = 165 Hz), 21.4 (d, *J* = 1 Hz).

^{19}F NMR (23 °C, $CDCl_3$, 470.55 MHz): δ = –204.1 (t, *J* = 48 Hz).

MS: 124 (M^+, 53 %), 123 ($[M – H]^+$, 21 %), 109 ($[M – CH_3]^+$, 100 %), 91 ($[M – CH_2F]^+$, 33 %), 77 ($[M – C_2H_4F]^+$, 12 %), 51 ($[M – C_4H_6F]^+$, 11 %).

3-(fluoromethyl)-1,1'-biphenyl (5c)

The title compound was prepared according to the general Procedure B as a colorless oil (85 mg, 90 %). Flash column chromatography was preformed using an isocratic elution with hexanes. Its spectroscopic data is consistent with the previously reported[22]. Spectroscopic data of isolated **5c** can be found on Page 46.

^{1}H NMR (23 °C, $CDCl_3$, 500.13 MHz): δ = 7.60 – 7.58 (m, 4H), 7.46 – 7.43 (m, 3H), 7.37 – 7.35 (m, 2H), 5.44 (d, *J* = 48 Hz, 2H).

^{13}C NMR (23 °C, $CDCl_3$, 125.76 MHz): δ = 141.8 (d, *J* = 1 Hz), 140.8 (s), 136.9 (s), 136.8 (s), 129.2 (d, *J* = 1 Hz), 129.0 (s), 127.7 (s), 127.7 (s), 127.3 (s), 126.5 (d, *J* = 2 Hz), 126.4 (d, *J* = 2 Hz), 84.8 (d, *J* = 167 Hz).

^{19}F NMR (23 °C, $CDCl_3$, 470.55 MHz): δ = –207.7 (t, *J* = 48 Hz).

MS: 186 (M^+, 100 %), 185 ($[M – H]^+$, 43 %), 165 (28 %).

1-bromo-4-(fluoromethyl)benzene (5d)

The title compound was prepared according to the general Procedure B as a white amorphous powder (79 mg, 84 %). Flash column chromatography was preformed using an isocratic elution with hexanes. Its spectroscopic data is consistent with the previously reported[23]. Spectroscopic data of isolated **5d** can be found on Page 47.

^{1}H NMR (29 °C, $CDCl_3$, 300.13 MHz): δ = 7.52 (dd, *J* = 8 Hz, 1 Hz, 2H), 7.24 (dd, *J* = 8 Hz, *J* = 2 Hz, 2H), 5.32 (d, *J* = 48 Hz, 2H).

^{13}C NMR (23 °C, $CDCl_3$, 125.76 MHz): δ = 135.3 (d, *J* = 17 Hz), 131.9 (d, *J* = 1 Hz), 129.2 (d, *J* = 6 Hz), 122.9 (d, *J* = 4 Hz), 83.9 (d, *J* = 167 Hz).

^{19}F NMR (23 °C, $CDCl_3$, 470.55 MHz): δ = –208.0 (t, *J* = 48 Hz).

MS: 190 ($[M + 2]^+$, 35 %), 189 ($[M + 1]^+$, 13 %), 188 (M^+, 35 %), 187 ($[M – H]^+$, 10 %), 109 ($[M – Br]^+$, 100 %) 108 (11 %), 107 (10 %), 83 (16 %).

1-bromo-3-(fluoromethyl)benzene (5e)

The title compound was prepared according to general Procedure A. Crude reaction mixture was analysed using ^{1}H NMR spectroscopy and GCMS. The product showed a characteristic doublet for benzylic protons at 5.35 ppm (J_{HF} = 47 Hz) in $CDCl_3$ (literature value 5.34 ppm (J_{HH} = 47 Hz) in $CDCl_3$[24]).

MS: 190 ($[M + 2]^+$, 48 %), 188 (M^+, 50 %), 109 ($[M – Br]^+$, 100 %), 83 (18 %).

1-fluoro-4-(fluoromethyl)benzene (5f)

The title compound was prepared according to general Procedure A. Crude reaction mixture was analysed using ^{1}H NMR spectroscopy and GCMS. The product showed a characteristic doublet for benzylic protons at 5.34 ppm (J_{HF} = 48 Hz) in $CDCl_3$ (literature value 5.39 ppm (J_{HH} = 48 Hz) in acetone[25]).

MS: 128 (M^+, 45 %), 127 ($[M – H]^+$, 100 %).

Ethyl 4-(fluoromethyl)benzoate (5g)

The title compound was prepared according to the general Procedure B as a colorless oil (75 mg, 83 %). Flash column chromatography was preformed using an isocratic elution with DCM : *n*-pentane = 1 : 4. Its spectroscopic data is consistent with the previously reported[26]. Spectroscopic data of isolated **5g** can be found on Page 48.

^{1}H NMR (23 °C, $CDCl_3$, 500.13 MHz): δ = 8.07 (d, *J* = 8 Hz, 2H), 7.43 (d, *J* = 7 Hz, 2H), 5.45 (d, *J* = 47 Hz, 2H), 4.39 (q, *J* = 7 Hz, 2H), 1.40 (t, *J* = 7 Hz, 3H).

^{13}C NMR (23 °C, $CDCl_3$, 125.76 MHz): δ = 166.4 (s), 141.2 (d, *J* = 17 Hz), 130.8 (d, *J* = 2 Hz), 130.0 (s), 126.7 (d, *J* = 6 Hz), 83.9 (d, *J* = 168 Hz), 61.2 (s), 14.5 (s).

^{19}F NMR (23 °C, $CDCl_3$, 470.55 MHz): δ = –213.2 (t, *J* = 47 Hz).

MS: 182 (M^+, 11 %), 154 ($[M - C_2H_4]^+$, 26 %), 137 ($[M - OCH_2CH_3]^+$, 100 %), 109 (46 %), 83 (15%).

1-(fluoromethyl)-3,5-bis(trifluoromethyl)benzene (5h)

The title compound was prepared according to the general Procedure B as a colorless oil (103 mg, 82 %). Flash column chromatography was preformed using an isocratic elution with *n*-pentane. Compound does not ionize under ESI+ or ESI– conditions. Products structure was elucidated using 2D NMR spectroscopy (HSQC and HMBC). Spectroscopic data of isolated **5h** can be found on Page 49.

^{1}H NMR (23 °C, $CDCl_3$, 500.13 MHz): δ = 7.88 (s, 1H), 7.83 (s. 2H), 5.52 (d, *J* = 47 Hz, 2H).

^{13}C NMR (23 °C, $CDCl_3$, 125.76 MHz): δ = 138.9 (d, *J* = 19 Hz, C_b), 132.3 (q, *J* = 34 Hz, 2C, C_d), 126.9 (m, 2C, C_c), 123.3 (q, *J* = 273 Hz, 2C, C_f), 122.6 (m, C_e), 82.8 (d, *J* = 171 Hz, C_a). For carbon spectrum assignation and lettering please refer to the attached physical copy on Page 49.

^{19}F NMR (23 °C, $CDCl_3$, 470.55 MHz): δ = –63.5 (s, 6F), –214.8 (t, *J* = 47 Hz, 1F).

MS: 246 (M^+, 37 %), 227 ($[M - F]^+$, 32 %), 177 ($[M - CF_3]^+$, 100 %), 127 ($[M - C_2F_5]^+$, 10 %).

1-(fluoromethyl)-4-nitrobenzene (5i)

The title compound was prepared according to the general Procedure B as a colorless oil (78 mg, 76 %). Flash column chromatography was preformed using an isocratic elution with DCM : *n*-pentane = 1 : 3. Its spectroscopic data is consistent with the previously reported[27]. Spectroscopic data of isolated **5i** can be found on Page 51.

^{1}H NMR (29 °C, $CDCl_3$, 300.13 MHz): δ = 8.26 (d, *J* = 8 Hz, 2H), 7.54 (d, *J* = 8 Hz, 2H), 5.51 (d, *J* = 47 Hz, 2H).

^{13}C NMR (23 °C, $CDCl_3$, 125.76 MHz): δ = 148.0 (s), 143.5 (d, *J* = 18 Hz), 127.2 (d, *J* = 7 Hz), 124.0 (s), 83.0 (d, *J* = 171 Hz).

^{19}F NMR (23 °C, $CDCl_3$, 470.55 MHz): δ = –216.2 (t, *J* = 47 Hz).

MS: 155 (M^+, 71 %), 109 ($[M - NO_2]^+$, 100 %), 107 (14 %), 97 (22 %), 89 (11 %), 83 (76 %), 63 (20 %), 57 (19 %), 50 (13 %), 39 (12 %), 30 (13 %).

1-(fluoromethyl)-3-methoxybenzene (5j)

The title compound was prepared according to general Procedure A. Crude reaction mixture was analysed using ^{1}H NMR spectroscopy and GCMS. The product showed a characteristic doublet for benzylic protons at 5.36 ppm (J_{HF} = 47 Hz) in $CDCl_3$ (literature value 5.36 ppm (J_{HH} = 47 Hz) in $CDCl_3$[28]).

MS: 140 (M^+, 100 %), 139 ($[M – H]^+$, 44 %), 125 ($[M – CH_3]^+$, 16 %), 109 (54 %), 77 (19 %).

1-chloro-4-(fluoromethyl)- benzene (5m)

The title compound was prepared according to general Procedure A. Crude reaction mixture was analysed using ^{1}H NMR spectroscopy and GCMS. The product showed a characteristic doublet for benzylic protons at 5.35 ppm (J_{HF} = 48 Hz) in $CDCl_3$ (literature value 5.40 ppm (J_{HH} = 48 Hz) in $CDCl_3$[27]).

MS: 146 ($[M + 2]^+$, 12 %), 144 (M^+, 37 %), 143 ($[M – H]^+$, 20 %), 109 ($[M – Cl]^+$, 100 %).

1,3-dichloro-2-(fluoromethy)benzene (5n)

The title compound was prepared according to general Procedure A. Crude reaction mixture was analysed using ^{1}H NMR spectroscopy and GCMS. The product showed a characteristic doublet for benzylic protons at 5.69 ppm (J_{HF} = 47 Hz) in $CDCl_3$ (literature value 5.60 ppm (J_{HH} = 48 Hz) in CCl_4[29]).

MS: 182 ($[M + 4]^+$, 4 %), 180 ($[M + 2]^+$, 23 %), 178 (M^+, 35 %), 145 (32 %), 144 (10 %), 143 ($[M – Cl]^+$, 100 %), 107 (30 %).

1-(fluoromethyl)-4-methoxybenzene (5q)

The title compound was prepared according to general Procedure A. Crude reaction mixture was analysed using ^{1}H NMR spectroscopy and GCMS. The product showed a characteristic doublet for benzylic protons at 5.30 ppm (J_{HF} = 49 Hz) in $CDCl_3$ (literature value 5.26 ppm (J_{HH} = 49 Hz) in $CDCl_3$[21]).

MS: 140 (M^+, 84 %), 139 ($[M – H]^+$, 100 %), 125 ($[M – CH_3]^+$, 10 %), 109 ($[M – OCH_3]^+$, 37 %), 97 (10 %), 96 (12 %), 95 (10 %), 77 (17 %), 51 (12 %).

Fluoromethylbenzene (5t)

The title compound was prepared according to general Procedure A. Crude reaction mixture was analysed using ^{1}H NMR spectroscopy and GCMS. The product showed a characteristic doublet for benzylic protons at 5.38 ppm (J_{HF} = 48 Hz) in $CDCl_3$ (literature value 5.39 ppm (J_{HH} = 48 Hz) in $CDCl_3$[30]).

MS: 110 (M^+, 49 %), 109 ($[M – H]^+$, 100 %), 83 (12 %).

S7 Fluorination of other substrates

1-fluorooctane (8a)

The title compound was prepared according to general Procedure A. Crude reaction mixture was analysed using ^{1}H NMR spectroscopy and GCMS. The product showed a characteristic doublet of triplets for –CH_2F 4.43 ppm (J_{HF} = 48 Hz, J_{HH} = 6 Hz) in $CDCl_3$ (literature value 4.46 ppm (J_{HF} = 48 Hz, J_{HH} = 6 Hz in $CDCl_3$[31]).

MS: 97 (4 %), 86 (6 %), 84 (27 %), 83 (31 %), 71 (23 %), 70 (57 %), 69 (28 %), 61 (17 %), 57 (51 %), 56 (45 %), 55 (34 %), 49 (13 %), 47 (15 %), 43 (100 %), 42 (30 %).

Oct-1-ene (side product in fluorination of 8a)

Elimination side product formed in 11% yield. Crude reaction mixture was analysed using ^{1}H NMR spectroscopy. The product showed a characteristic multiplets for –C*H*=C*H*$_2$ protons at 5.80 ppm and 4.97 ppm in $CDCl_3$ (literature values 5.77 ppm and 4.97 in $CDCl_3$[32]).

3α-fluoro-5α-cholestane (8b)

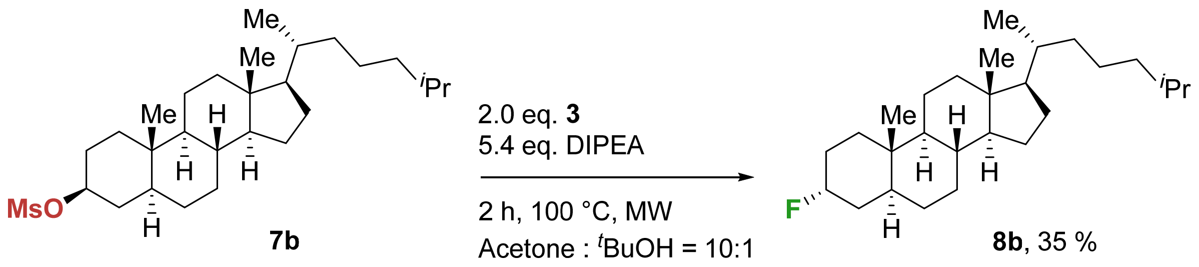

Scheme S7 1: Fluorination of 7b

To 22.8 mg (0.049 mmol, M = 466.76 g mol^{-1}) of **7b**, dissolved in 1 ml of acetone, were added 43.7 mg (0.097 mmol, M = 448.61 g mol^{-1}) of **3**, 40 μL (0.230 mmol, M = 129.24 g mol^{-1}) of DIPEA and 100 μL of *tert*-butanol. Reaction mixture was heated under microwave irradiation at 100 °C and increased pressure for 2 h. After the reaction, solvent was removed under reduced pressure. Diethyl ether was introduced and precipitated imidazolium salts were filtered off. Diethyl ether was removed under reduced pressure and crude product was purified using flash column chromatography (mobile phase: *n*-hexane) to yield **8b** as a white solid (6.7 mg, 35 %). Its spectroscopic data is consistent with the previously reported[33]. Spectroscopic data of isolated **8b** can be found on Page 52.

^{1}H NMR (29 °C, $CDCl_3$, 300.13 MHz): δ = 4.80 (dm, *J* = 49 Hz, 1H), 2.02–0.67 (m, accurate integration is not possible due to overlap), 0.78 (s, 3H), 0.65 (s, 3H).

^{13}C NMR (23 °C, $CDCl_3$, 125.76 MHz): δ = 89.8 (d, *J* = 166 Hz), 56.6, 56.4, 54.2, 42.7, 40.1, 39.7, 39.5, 36.3, 36.0, 35.8, 35.6, 34.2, 34.0, 32.6, 32.1, 28.5, 28.4, 28.2, 27.3, 27.2, 24.3, 24.0, 23.0, 22.7, 20.9, 18.8, 12.2, 11.3, 11.3 (assignation of all peak multiplicities was not attempted).

^{19}F NMR (23 °C, $CDCl_3$, 470.55 MHz): δ = –181.3 (m).

MS: 390 (M^+, 16 %), 375 ($[M – CH_3]^+$, 15 %), 236 (46 %), 235 (100 %), 221 (25 %), 217 (11 %), 168 (13 %), 167 (31 %), 123 (16 %), 122 (11 %), 121 (15 %), 109 (17 %), 108 (20 %), 107 (24 %), 105 (12 %), 95 (30 %), 93 (22 %), 91 (15 %), 83 (10 %), 81 (30 %), 79 (20 %), 69 (18 %), 67 (23 %), 57 (18 %), 55 (30 %), 43 (33 %), 41 (23 %).

5α-cholest-3-ene and 5α-cholest-2-ene[34,35] (side products in fluorination of **8b**)

Side products formed during fluorination of 5α-choletan-3β-yl mesylate (**7b**) were isolated as a mixture of alkenes with 65% yield as determined by characteristic signals in the NMR spectra (5.59 (m), 5.31–5.24 (m) and molecular ion peak (M^+) in MS at 370.

2-fluoro-1-(4-methoxyphenyl)ethan-1-one (**8c**)

The title compound was prepared according to general Procedure A. Crude reaction mixture was analysed using ^{1}H NMR spectroscopy and GCMS. The product showed a characteristic doublet for –CH_2F at 5.45 ppm (J_{HF} = 47 Hz) in $CDCl_3$ (literature value 5.48 ppm (J_{HH} = 47 Hz) in $CDCl_3$[36]).

MS: 168 (M^+, 13 %), 135 ($[M – CH_2F]^+$, 100 %), 107 (10 %), 92 (19 %), 77 (28 %).

2-fluoro-1,2-diphenylethan-1-one (**8d**)

The title compound was prepared according to general Procedure A. Crude reaction mixture was analysed using ^{1}H NMR spectroscopy and GCMS. The product showed a characteristic doublet for –CHF at 6.54 ppm (J_{HF} = 49 Hz) in $CDCl_3$ (literature value 6.52 ppm (J_{HH} = 49 Hz) in $CDCl_3$[36]).

MS: 214 (M^+, 0.1 %), 105 (100 %), 77 (37 %), 51 (10 %).

(4-chlorophenyl)methanol (**8e**)

The title compound was prepared according to general Procedure A. Crude reaction mixture was analysed using ^{1}H NMR spectroscopy and GCMS. The product showed a characteristic singlet for benzylic protons at 4.62 ppm in $CDCl_3$ (literature value 4.61 ppm in $CDCl_3$[37]).

MS: 144 ($[M + 2]^+$, 16 %), 142 (M^+, 48 %), 141 ($[M – H]^+$, 12 %), 125 ($[M – OH]^+$, 12 %), 123 (17 %), 107 (64 %), 89 (11 %), 79 (72 %), 77 (100 %), 75 (15 %), 51 (24 %), 50 (17 %), 28 (12 %).

Tert-butyldimethylsilyl fluoride (**8f**)

The title compound was prepared according to general Procedure A. Crude reaction mixture was analysed using ^{1}H and ^{19}F NMR spectroscopy. The product showed characteristic doublet for –$C(CH_2)_2F$ at 0.14 ppm (J_{HF} = 8 Hz) and doublet for $(CH_3)_3C$– at 0.90 ppm (J_{HF} = 1 Hz) in $CDCl_3$ (literature values 0.22 Hz ppm (J_{HF} = 8 Hz) and 0.98 ppm (J_{HF} = 1 Hz) in $CDCl_3$[38]).

^{19}F NMR: –171.0 ppm (m).

4-nitrobenzoyl fluoride (8g)

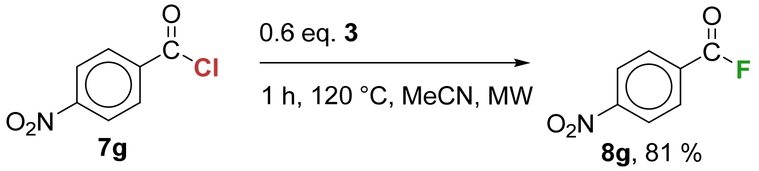

Scheme S7 2: Fluorination of 7g

To 31.2 mg (0.168 mmol, M = 185.56 g mol^{-1}) of **7g**, recrystallized from benzene, dissolved in 1 ml of dry acetonitrile, was added 45.3 mg (0.101 mmol, M = 448.61 g mol^{-1}) of **3**. Reaction was carried out under microwave irradiation at 120 °C and increased pressure for 1 h. After the reaction, acetonitrile was removed under reduced pressure. A few millilitres of mobile phase (DCM : petroleum ether = 9 : 10) was poured over residue causing imidazolium salts to precipitate. Obtained solution of crude product was purified using flash column chromatography to yield **8g** as a white solid (22.9 mg, 81 %). Products spectroscopic data is consistent with the previously reported[30]. Spectroscopic data of isolated **8g** can be found on Page 53.

^{1}H NMR (29 °C, $CDCl_3$, 300.13 MHz): δ = 8.39 (dd, *J* = 9 Hz, *J* =1 Hz, 2H), 8.26 (d, *J* = 9 Hz, 2H).

^{13}C NMR (29 °C, $CDCl_3$, 75.47 MHz): δ = 155.6 (d, *J* = 346 Hz), 152.0 (s), 132.7 (d, *J* = 4 Hz), 130.4 (d, *J* = 63 Hz), 124.3 (d, *J* = 1 Hz).

^{19}F NMR (23 °C, $CDCl_3$, 470.55 MHz): δ = 20.9 (s).

4-methylbenzenesulfonyl fluoride (8h)

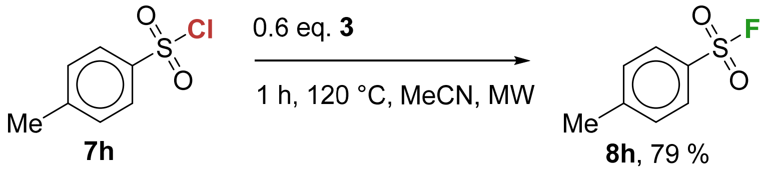

Scheme S7 3: Fluorination of 7h

To 32.0 mg (0.167 mmol, M = 190.65 g mol^{-1}) of **7h**, dissolved in 1 ml of acetonitrile, was added 45.2 mg (0.101 mmol, M = 448.61 g mol^{-1}) of **3**. Reaction was carried out under microwave irradiation at 120 °C for 1 h. After the reaction, acetonitrile was removed under reduced pressure. A few millilitres of mobile phase (DCM : petroleum ether = 1 : 3) was poured over residue causing imidazolium salts to precipitate. Obtained solution of crude product was purified using flash column chromatography to yield **8h** (23.2 mg, 79 %, colorless oil). Products spectroscopic data is consistent with the previously reported[39]. Spectroscopic data of isolated **8h** can be found on Page 54.

^{1}H NMR (29 °C, $CDCl_3$, 300.13 MHz): δ = 7.89 (d, *J* = 8 Hz, 2H), 7.42 (d, *J* = 9 Hz, 2H), 2.49 (s, 3H).

^{13}C NMR (29 °C, $CDCl_3$, 75.47 MHz): δ = 147.2 (s), 130.4 (s), 130.1 (s), 128.6 (s), 21.9 (s).

^{19}F NMR (23 °C, $CDCl_3$, 470.55 MHz): δ = 65.8 (s).

1-fluoro-4-nitrobenzene (8i)

The title compound was prepared according to general Procedure A. Crude reaction mixture was analysed using ^{1}H NMR spectroscopy and GCMS. The product showed a characteristic multiplet at 8.25 ppm in $CDCl_3$ (literature value 8.30 ppm in $CDCl_3$[40]).

MS: 141 (M^+, 97 %), 111 ($[M – NO]^+$, 37 %), 95 ($[M – NO_2]^+$, 100 %), 83 (27%), 75 (68 %), 69 (12 %), 57 (11 %), 50 (15 %), 28 (24 %).

S8 Synthesis of reagent $[IPrH][H_2F_3]$ (3) under ambient conditions

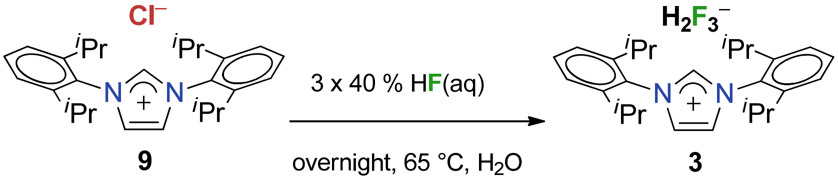

Scheme S8 1: Synthesis of reagent $[IPrH][H_2F_3]$ (3)

21.80 g of [IPrH][Cl] (**9**) was dissolved in 420 ml of deionized water in a PP plastic reactor and heated to 65°C. To a separate PTFE beaker, an excess volume of 40% Hydrofluoric acid is measured (120 ml, 54.8 equivalents) and poured over the original solution of **9**. An off-white precipitate forms. The reaction mixture is stirred at 65°C until all volatiles evaporate. The white dry residue is scraped off reactor walls, 420 ml of water is added and formed suspension is treated with Hydrofluoric acid three more times. The product $[IPrH][H_2F_3]$ (**3**) is isolated as a white crystalline powder (19.48 g; 85% yield). Identity and purity of the product were confirmed with single crystal XRD analysis, and multinuclear NMR spectroscopy. Elemental analysis after three treatments shows negligible amount of Cl^- ions (Table S8 2, entry 3). All the analyses are in agreement with already published data[41].

Influence of molar ratios between 9 and HF and details on synthesis optimization.

Influence of molar ratio between HF and precursor **9** in the reaction mixture was studied first. Precursor **9** (2.0 g, 4.71 mmol, M = 425.05 g mol^{-1}) was dissolved in 80 ml of deionized water. Exact volumes of hydrofluoric acid (40%), which corresponded to 3, 10, 50, and 100 equivalents of **9**, were added (Table S8 1) in a single addition. The reaction mixtures were stirred at 65 °C until all volatilities evaporated. The resulting degree of anion substitution was determined by elemental analysis of crude product (fluoride mass fraction with fluoride ion selective electrode and chloride mass fraction with gravimetric analysis; Table S8 1 and Figure S8 1).

Table S8 1: Mass fractions in the crude product obtained after single addition of hydrofluoric acid.

Entry	eq. $HF_{(aq)}$	V ($HF_{(aq)}$) [ml]	w_F [%]	w_{Cl} [%]
1	3	0.15	4.7	6.6
2	10	0.52	7.4	4.7
3	50	2.6	10.1	2.4
4	100	5.2	10.3	2.4

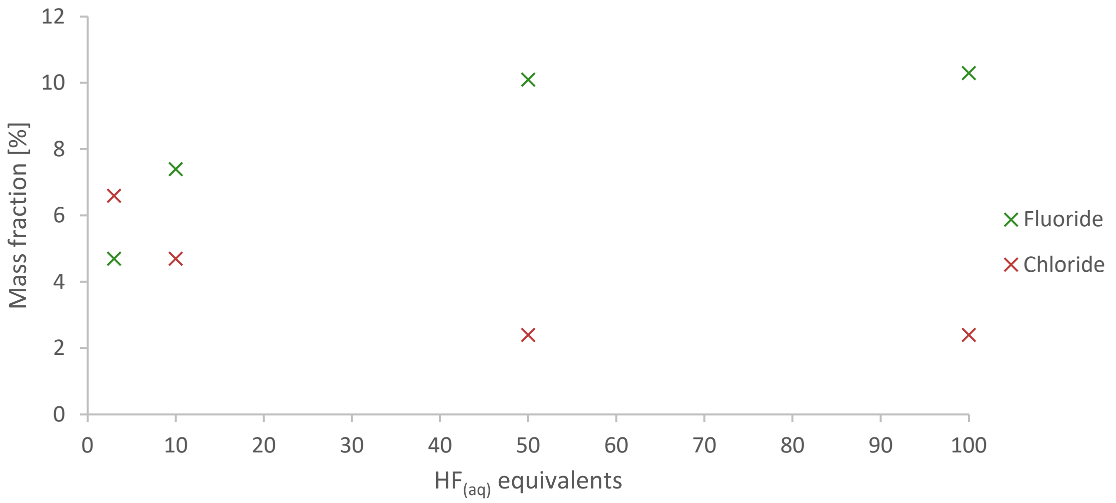

Figure S8 1: Influence of HF equivalents on mass fractions in the product after single addition of hydrofluoric acid

These results show that fluoride content cannot be fully substituted by using large excess of Hydrofluoric acid (> 50 eq.) in a single addition (Table S8 1 and Figure S8 1). One treatment of **9** with hydrofluoric acid afforded only up to w_F = 10.3% (theoretical max. w_F = 12.7%). The addition of fifty-fold excess of hydrofluoric acid was repeated two more times to increase the fluoride content and obtain sufficiently pure **3** (Table S8 2, entry 3).

*Table S8 2: Final mass fractions in the [IPrH][H_2F_3] (**3**) after three treatments with $HF_{(aq)}$*

Number of repeats	w_F [%]	w_{Cl} [%]
1	10.3	2.6
2	11.5	2.4
3	12.2	0.2
Calculated for **3**	12.7	0

Chloride mass fraction was negligible after the third treatment with hydrofluoric acid and the obtained reagent was used in fluorination reactions without further purification.

For all further syntheses of the reagent (**3**) we performed four additions of Hydrofluoric acid as a precaution and additionally verified the purity of each batch with the ^{1}H and ^{19}F NMR spectroscopy.

S9 Post-fluorination recovery of reagent 3

Post-fluorination procedures were divided into *isolation procedures* and *regeneration procedures*. The first part deals with isolation of fluorinated product and imidazolium salts from reaction mixture, while the second part addresses the regeneration procedure of isolated imidazolium salts back to reagent **3**. Isolation is performed with commonly used solvents, while regeneration is achieved without the use of organic solvents, using only hydrofluoric acid or anhydrous HF.

Isolation procedure for fluorinated product and imidazolium salts

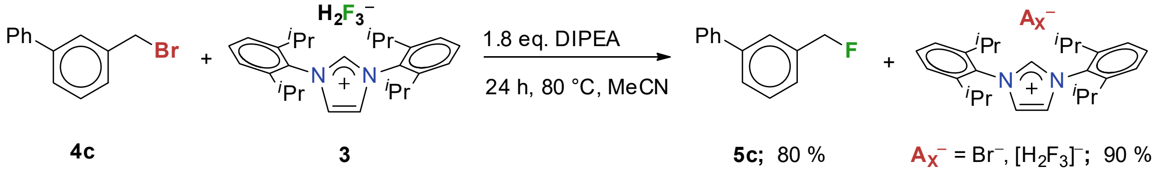

Scheme S9 1: Experiment for isolation of fluorinated product and imidazolium salts

FEP reactor was charged with 0.9181 g (3.71 mmol, M = 247.13 g mol^{-1}) of **4c** and 3 ml of acetonitrile. To the resulting solution were added: 1.0000 g (2.23 mmol, M = 448.61 g mol^{-1}) of **3**, 1.15 ml (6.60 mmol, M = 129.24 g mol^{-1}) DIPEA and additional 7 ml of acetonitrile. Reaction mixture was heated at 80 °C for 24 hours. After the reaction, a few drops of Et_2NH was added and volatiles (acetonitrile and DIPEA) were removed under the reduced pressure. 10 ml of diethyl ether was added to the obtained oily residue causing imidazolium salts to precipitate. Imidazolium salts were then filtered off and subsequently washed with three additional portions of diethyl ether, 10 ml each. Ethereal solutions were combined and dried under reduced pressure. Crude product was purified with flash column chromatography (mobile phase: DCM : mixture of hexanes = 1 : 5) to yield **5c** as a colorless liquid (0.5563 g, 80 %). Filtered imidazolium salts were further washed with 10 ml of water, 10 ml of THF, 10 ml of EtOAc, and thoroughly dried after each washing to obtain white powder (0.9387 g, 90 %, Figure S9 1).

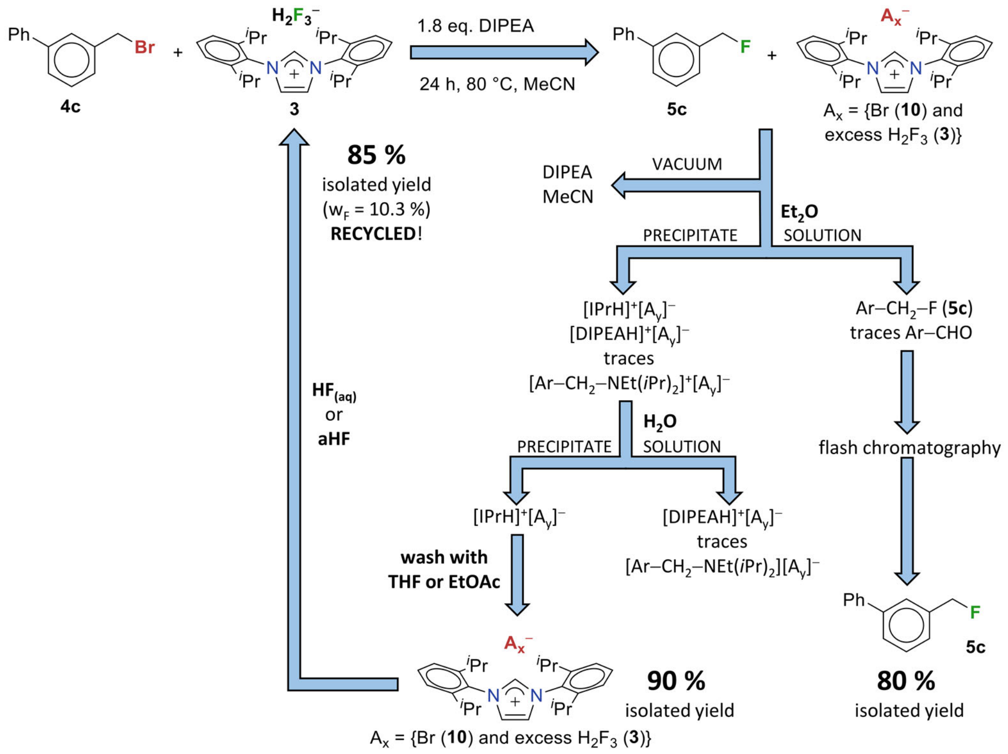

Figure S9 1: Full separation scheme for isolation of imidazole salts and fluorinated product. Imidazolium salts can then be recycled back to reagent 3.

$[A_X]^-$ represents Br^- and $[H_2F_3]^-$ anions. Therefore, the imidazolium salts with a general formula [IPrH]$[A_x]$ represent a mixture consisting predominantly of [IPrH]Br (**10**) and small excess of [IPrH]$[H_2F_3]$ (**3**). Although reagent **3** is used in substoichiometric quantities (0.6 eq.) for fluorination reactions, the $[H_2F_3]^-$ anion contains three fluorides (1.8 eq.). Fluoride is therefore used in excess and part of the unreacted reagent **3** is precipitated together with **10**, due to relatively similar solubility of both salts in organic solvents. Separation of **3** and **10** was not pursued as it was not necessary for regeneration purposes. We also note, that the counter ion with a general label $[A_y]^-$ could be a number of different species, which we did not pursue to identify, however the structural motives of the cations were easily characterized. Isolation of imidazolium salts proceeds easily with the use of common organic solvents. Additional research should be conducted to further optimize separation protocols. Increased yields and reduced solvent consumption could be achieved with more developed and tailored purification scheme.

Total elemental composition of the isolated imidazolium salts was determined with combination of CHN analysis and ion chromatography. These analyses confirm that imidazolium salts with general formula [IPrH]$[A_x]$ are a mixture of bromide ($w_{Br,\ EXP.}$ = 15.4 %) and poly(hydrogen fluoride) ($w_{F,EXP.}$ = 1.6 %) imidazolium salts (Table S9 1).

*Table S9 1: Elemental analysis of the isolated [IPrH]Br (**10**) with excess [IPrH][H_2F_3] (**3**) after the fluorination reaction*

	w_N [%]	w_C [%]	w_H [%]	w_{Br} [%]	w_F [%]
Experimental	5.89	69.61	8.07	15.4	1.6

Additionally, ^{1}H and ^{19}F NMR spectra (Figure S9 2 and Figure S9 3) of the imidazolium salt mixture [IPrH]$[A_x]$ are consistent with the results of elemental analyses. However, ^{1}H NMR spectrum of [IPrH]$[A_x]$ shows the absence of $[H_2F_3]^-$ proton signal at ≈ 13.4 ppm present in the mixture. Nevertheless, ^{19}F NMR spectrum shows a characteristic signal for $[H_2F_3]^-$ anion. The absence of ^{1}H signal can be explained by low concentration of [IPrH]$[H_2F_3]$ and inherently broad ^{1}H NMR signal of $[H_2F_3]^-$ anion[41].

Spectroscopic data of recovered imidazolium salts **[IPrH][A$_x$]**
A$_x$ = Br$^-$, $[H_2F_3]^-$

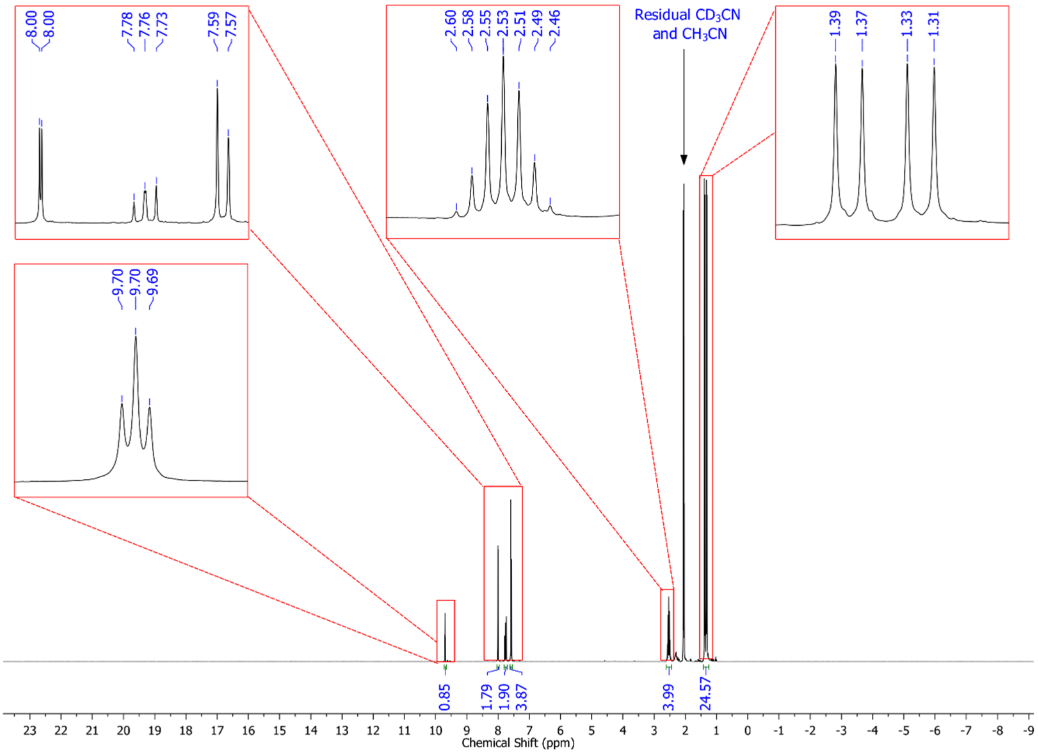

Figure S9 2: ^{1}H NMR of recovered imidazolium salts [IPrH][A$_x$] (CD_3CN, 25 °C, 302.97 MHz)

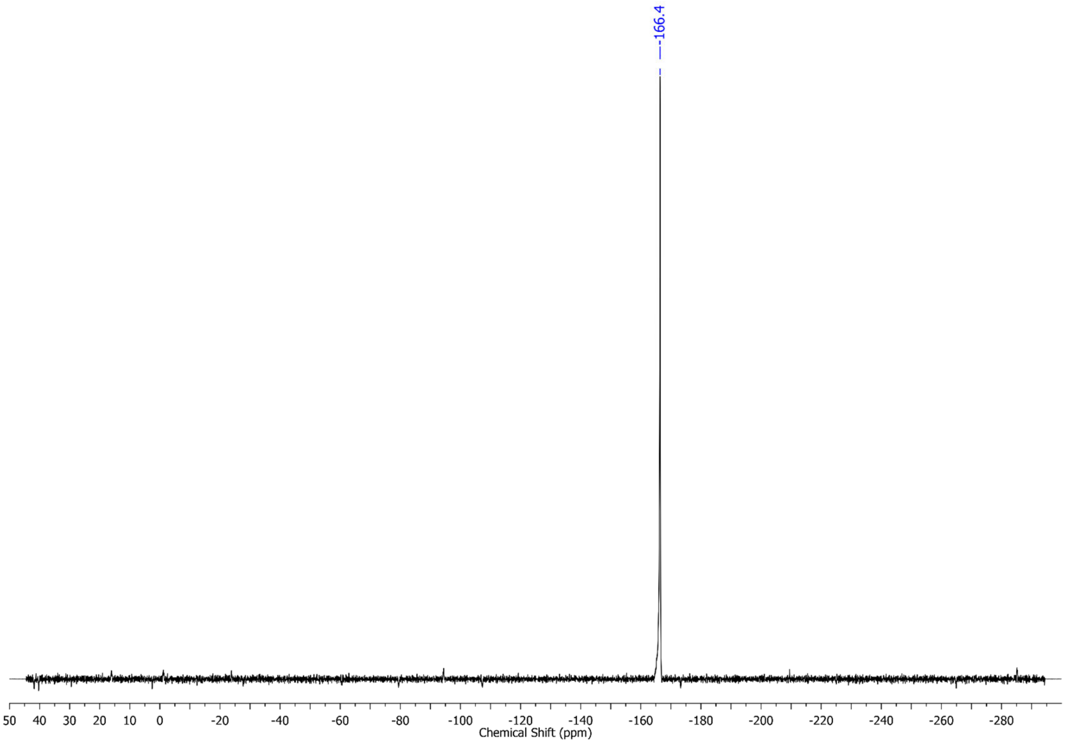

Figure S9 3: ^{19}F NMR of recovered imidazolium salts [IPrH][A$_x$] (CD_3CN, 25 °C, 285.04 MHz)

Regeneration procedure of imidazolium salt [IPrH][A_x] to reagent **3**

a) Regeneration of **3** with hydrofluoric acid

Once the imidazolium salts [IPrH][A_x] (A_x represents Br^- and $[H_2F_3]^-$ anions of compounds **10** and **3** respectively in the recovered imidazolium salts; see Section S9) were isolated, the regeneration process back to reagent **3** was implemented.

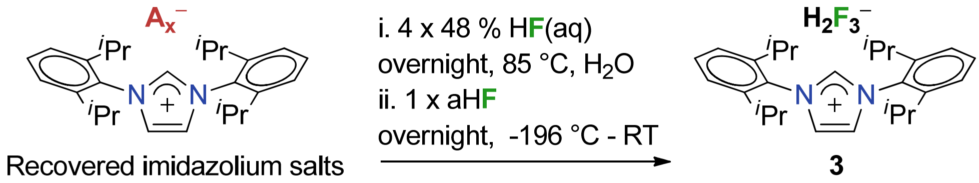

Scheme S9 2: Regeneration of 3 with hydrofluoric acid

Step i) In a plastic beaker 490.3 mg of purified imidazolium salts from previous step were dissolved in 100 ml of deionized water at 85 °C. 4 ml of 48 % hydrofluoric acid was added. Reaction mixture was left stirring overnight at 85 °C. All volatiles evaporated and procedure was repeated three more times. After regeneration a few milliliters of acetonitrile was added and remaining precipitate was filtered off. Acetonitrile was removed under reduced pressure overnight to give regenerated **3** (404.8 mg). Elemental composition of the obtained reagent was determined with combination of CHN analysis and ion chromatography (F^- / Br^-) (Table S9 2).

Table S9 2: Elemental composition of regenerated 3 with hydrofluoric acid

	w_N [%]	w_C [%]	w_H [%]	w_F [%]	w_{Br} [%]
Experimental	6.15	72.49	8.87	10.3	2.5
Theoretical (**3**)	6.24	72.29	8.76	12.7	0

A small weight percentage of bromide (w_{Br} = 2.5 %) persisted and could not be substituted with hydrofluoric acid. The maximum fluoride weight percentage that could be obtained was w_F = 10.3 %. Therefore, the regeneration process for **3** in hydrofluoric acid is conditioned by a compatible counter anion (*e.g.* in the case of chloride in [IPrH][Cl] (**9**), the substitution is quantitative; see Section S8, Table S8 2). Partial substitution should not present a problem, if the reagent is used in excess and returned to the same reaction system. Nevertheless, we completely regenerated reagent **3** with anhydrous HF (in step ii).

Step ii) Approximately 1 ml of anhydrous HF was condensed over 134 mg of recovered imidazolium salts (from step i) in FEP reactor at –196 °C. Reaction mixture was brought to room temperature and was left to stir overnight. Anhydrous HF was removed under reduced. Elemental composition of the obtained reagent was determined with combination of CHN analysis and ion chromatography (F^- / Br^-) (Table S9 3).

Table S9 3: Elemental composition of recycled 3 with additional anhydrous HF

	w_N [%]	w_C [%]	w_H [%]	w_F [%]	w_{Br} [%]
Experimental	6.17	72.48	8.65	12.8	0
Theoretical (**3**)	6.24	72.29	8.76	12.7	0

^1H and ^{19}F NMR spectra of the recycled **3** were recorded and they completely correspond with aforementioned elemental analysis and literature (Figure S9 4 and Figure S9 5)[41].

Spectroscopic data of recycled **3** with hydrofluoric acid and anhydrous HF is consistent with the following literature[41].

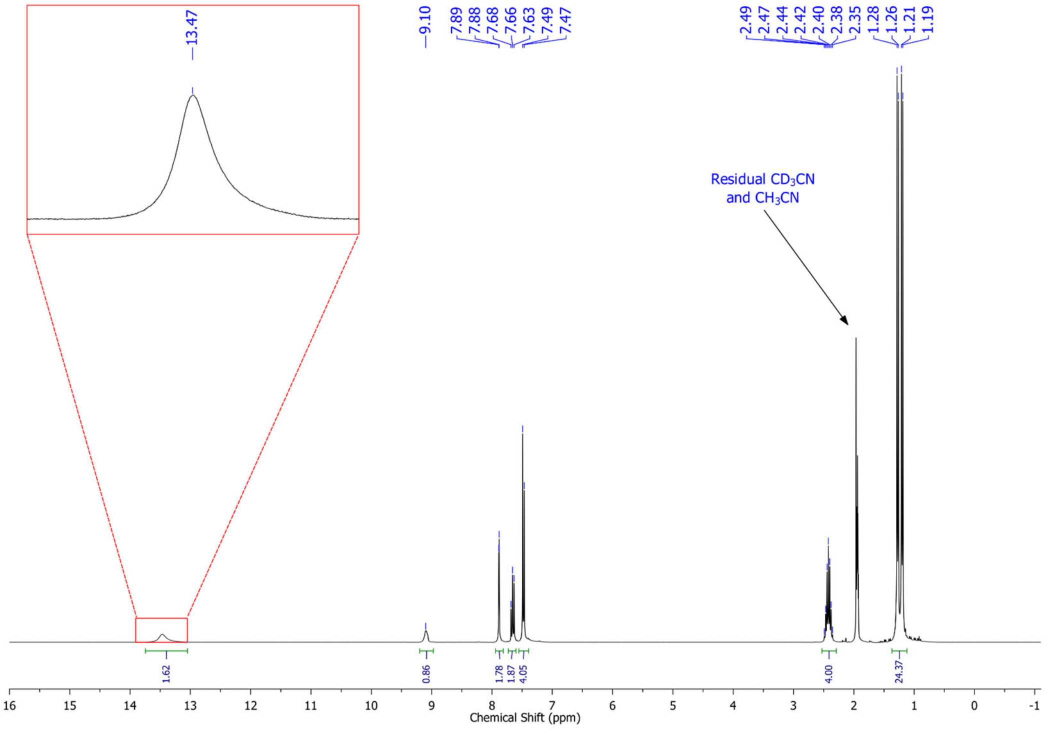

*Figure S9 4: ^{1}H NMR of recycled **3** (CD_3CN, 25 °C, 302.97 MHz)*

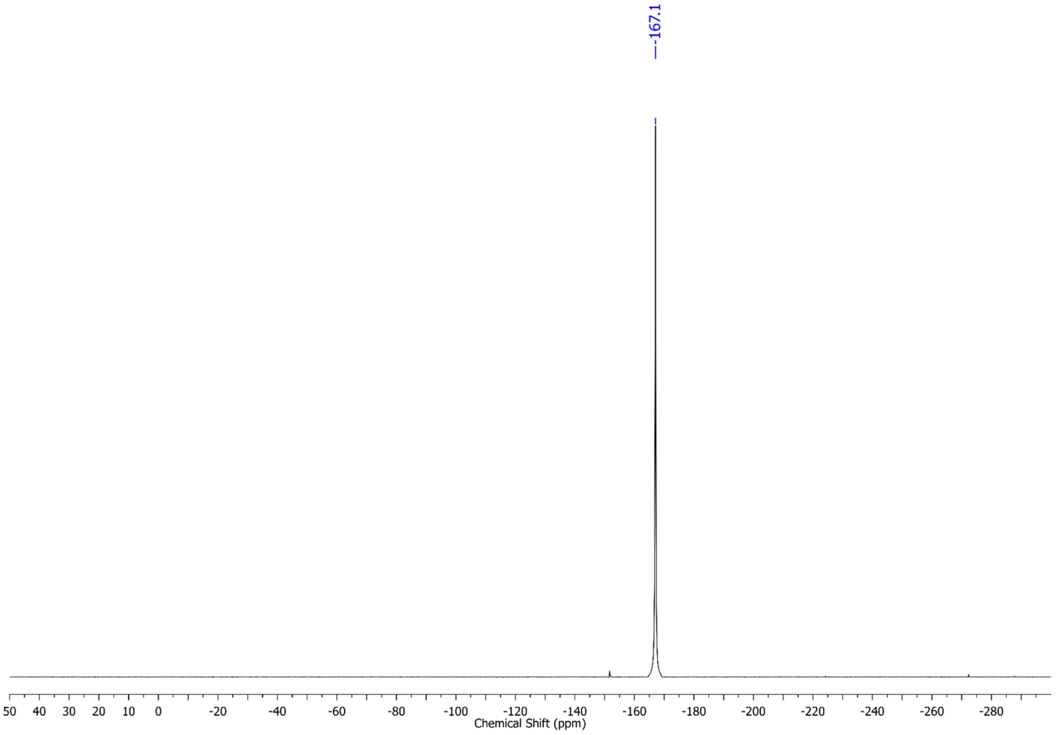

Figure S9 5: ^{19}F NMR of recycled 3 (CD_3CN, 25 °C, 285.04 MHz)

b) Regeneration of **3** with anhydrous HF

The results from previous experiment lead us to a regeneration procedure of **3** from recovered imidazolium salts using anhydrous HF alone. In this way, fully regenerated **3** can be prepared in a single step (Table S9 4).

Experimental procedure for regeneration of **3** with anhydrous HF:

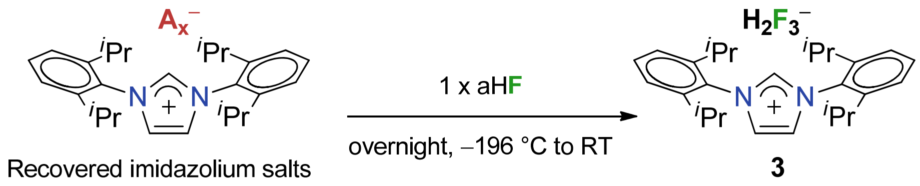

*Scheme S9 3: Regeneration of the reagent **3** with single addition of anhydrous HF*

$[A_x]^-$ represents Br^- and $[H_2F_3]^-$ anions of compounds **10** and **3** respectively in the recovered imidazolium salts (see Section S9).

Under inert conditions approximately 1 ml of aHF was condensed over 96 mg of purified imidazolium salts in a custom made FEP reactor at –196 °C. Reaction mixture was brought to room temperature and was left to stir overnight. aHF was removed under reduced pressure overnight. A few millilitres of acetonitrile was added and the obtained solution was filtered. Acetonitrile was removed under reduced pressure overnight and elemental composition of the recycled reagent was determined with combination of CHN analysis and ion chromatography (F^- / Br^-) (Table S9 4).

*Table S9 4: Elemental composition of recycled **3** upon single treatment of $[IPrH][A_x]$ with anhydrous HF*

	w_N [%]	w_C [%]	w_H [%]	w_F [%]	w_{Br} [%]
Experimental	6.12	72.32	8.85	13.0	0
Theoretical (**3**)	6.24	72.29	8.76	12.7	0

^1H and ^{19}F NMR spectra of the recycled **3** were recorded and they completely correspond with aforementioned elemental analysis and literature[41] (Figure S9 6 and Figure S9 7).

Spectroscopic data of recycled **3** with anhydrous HF is consistent with the following literature[41].

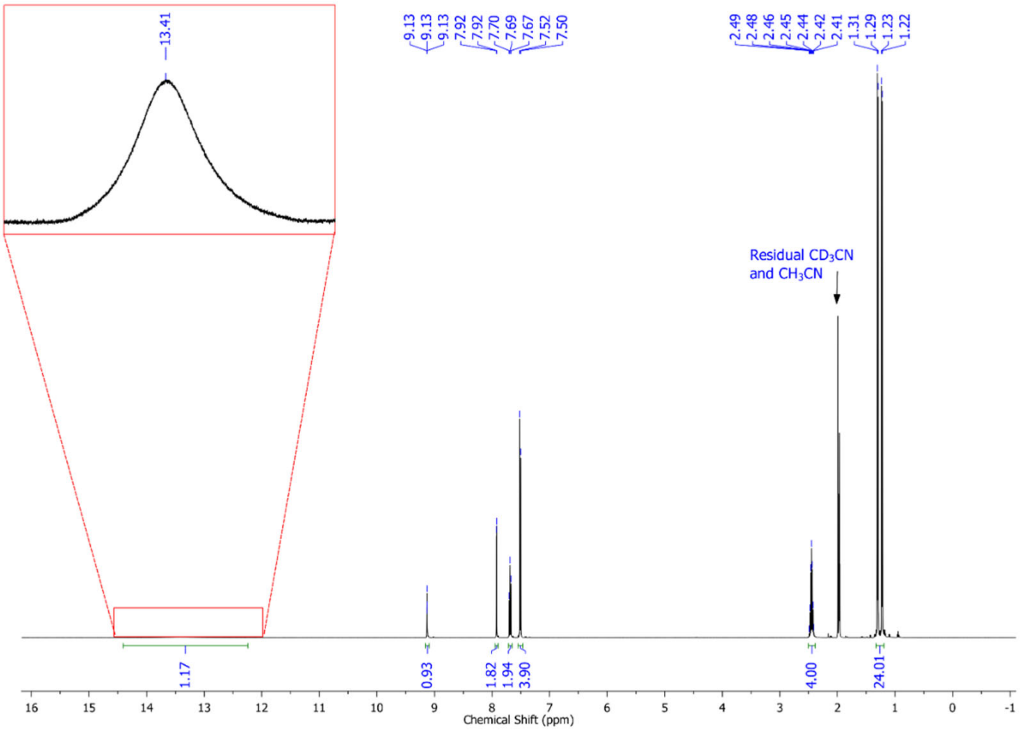

Figure S9 6: ^{1}H NMR of recycled 3 (CD_3CN, 25 °C, 500.13 MHz)

$[H_2F_3]^-$ protons are not visible in the full spectrum due to broadening of the corresponding signal (zoomed in signal can be seen in the upper right corner of the spectrum).

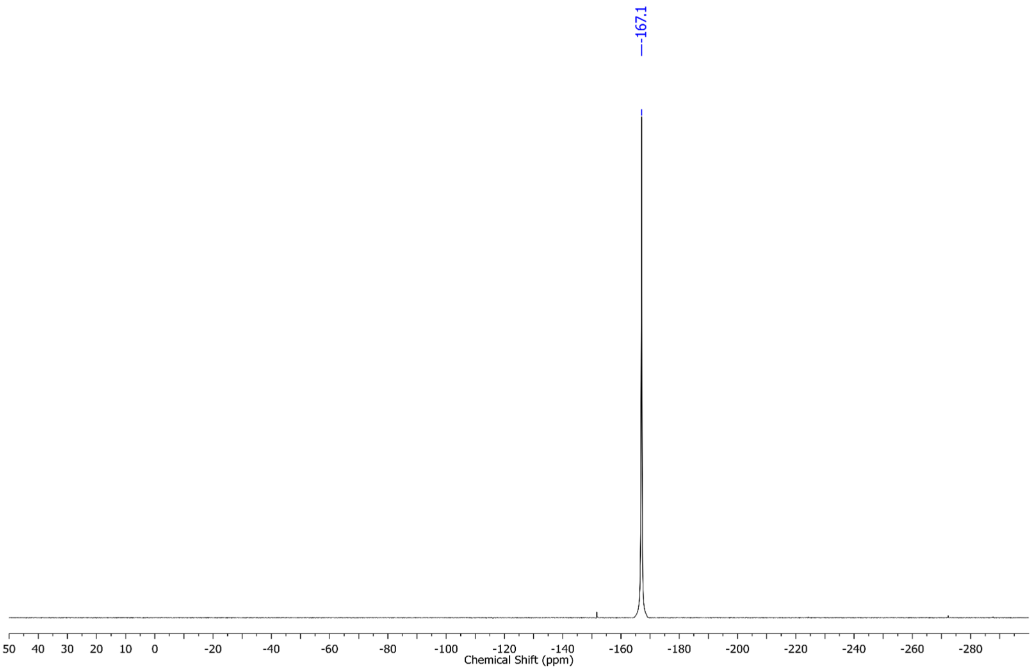

Figure S9 7: ^{19}F NMR of recycled 3 (CD_3CN, 25 °C, 285.04 MHz)

S10 Synthesis and characterization of **6**

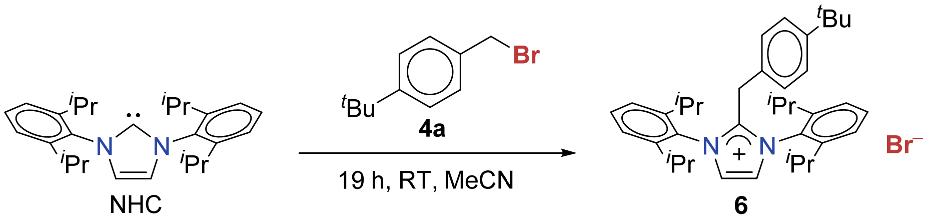

Scheme S10 1: Synthesis of product 6

Schlenk flask was charged with 210 mg (0.540 mmol, M = 388.59 g mol^{-1}) of NHC under inert atmosphere. Anhydrous acetonitrile was added, followed by 100 µL of **4a** (0.544 mmol, M = 227.14 g mol^{-1}). Reaction mixture was stirred at room temperature for 19 h. Solvent was removed under vacuum. Crude product (215 mg, 65 %) was washed with anhydrous toluene and dried to give yellow powder. ^{1}H, ^{13}C, HSQC and HMBC spectra of the 2-(4-(tert-butyl)benzyl)-1,3-bis(2,6-diisopropylphenyl)-1H-imidazol-3-ium bromide (**6**) were recorded in CD_3CN. Compounds identity was additionally confirmed with high resolution mass spectrometry. Determined elemental composition is shown in Table S10 1. Single crystals of the title compound solvate were prepared by slow evaporation of acetonitrile solution under anhydrous conditions. Spectroscopic data of isolated **6** can be found on Page 55.

^{1}H NMR (29 °C, CD_3CN, 300.13 MHz): δ = 8.00–7.92 (m, 2H), 7.68 (t, J = 8 Hz, 2H), 7.47 (d, J = 8 Hz, 4H), 7.12 (d, J = 9 Hz, 2H) 6.37 (d, J = 9 Hz, 2H), 3.86 (s, 2H), 2.29 (sep, 4H), 1.25–1.09 (m, 33H).

^{13}C NMR (29 °C, CD_3CN, 75.47 MHz): δ = 152.0 (s, C_a), 148.2 (s, C_h), 146.3 (s, C_k), 133.4 (s, 2C, C_o)), 130.4 (s, C_j), 129.8 (s, C_e), 128.3 (s, C_f), 127.0 (s, C_d), 126.5 (s, 2C, C_i), 126.2 (s, 4C, C_n), 35.1 (s, C_b), 31.3 (s, C_a), 31.0 (s, C_g), 30.2 (s, C_l), 25.9 (s, 4C, C_m), 22.3 (s, 4C, $C_{m'}$). For carbon spectrum assignation and lettering please refer to the attached physical copy on Page 55.

HRMS (ESI–): calculated mass for $C_{35}H_{51}N_2Br_2$ (M+Br$^-$): 693.2419. Experimental mass: 693.2413.

Elemental analysis:

*Table S10 1: Experimental and theoretical composition of **6***

	w_N [%]	w_C [%]	w_H [%]
Experimental values	4.51	74.27	8.46
Theoretical composition	4.54	74.00	8.50

Single Crystal XRD analysis of compound of **6**

The unit cell contains two asymmetric units related by an inversion center. The product crystalizes as a solvate in $P\overline{1}$ space group. The asymmetric unit consists of imidazolium cation, bromine anion and a molecule of acetonitrile solvent. Bond lengths are consistent to those reported in the analogous molecules[42]. Newly formed bond C2–C30 is consistent with average Csp^3–Csp^2 exocyclic bond, while the C30–C31 bond length completely coincide with an average Csp^3–CAr bond length[43]. Ellipsoids are drawn with 50% probability and the cation's N, N - substituents are shaded and MeCN molecule omitted for clarity. Find the basic collection and refinement parameters in Table S10 2, bond lengths in Table S10 3 and angles in

Table S10 4. Asymmetric unit is shown on Figure S10 1

*Table S10 2: Basic collection and refinement data for structure **6***

Compound	6
CCDC Number	2054235
Formula	$C_{38}H_{51}N_2{\cdot}C_2H_3N{\cdot}Br$
F_w	656.77
T (K)	150
Crystal system / group	Triclinic / $P\overline{1}$
a (Å)	10.1039 (2)
b (Å)	11.3314 (2)
c (Å)	17.4087 (3)
α (°)	100.5((2)
β (°)	90.723 (2)
γ (°)	104.483 (2)
V (Å^3)	1893.85 (6)
Z	2
Mass density (g/cm^3)	1.152
Crystal size (mm)	0.41 × 0.35 × 0.29
Radiation type	Cu $K\alpha$
λ (Å)	1.54184
μ (mm^{-1})	1.68
F (000)	700
Θ_{max} (°)	73.2
Index ranges	−12 ≤ h ≤ 12<br>−13≤ k ≤ 14<br>−21 ≤ l ≤ 21
Reflections collected	52517
Independent reflections	7476
Reflections with [I > 2σ(I)]	6819
R_{int}	0.044
Data/restraints/parameters	7476/0/409
R_1[b], wR_2[c] [$I > 2\sigma(I)$]	0.034, 0.084
R_1[b], wR_2[c] [all data]	0.038, 0.087
$\Delta\rho_{min,}\ \Delta\rho_{max,}$(eÅ^{-3})	-0.44, 0.59

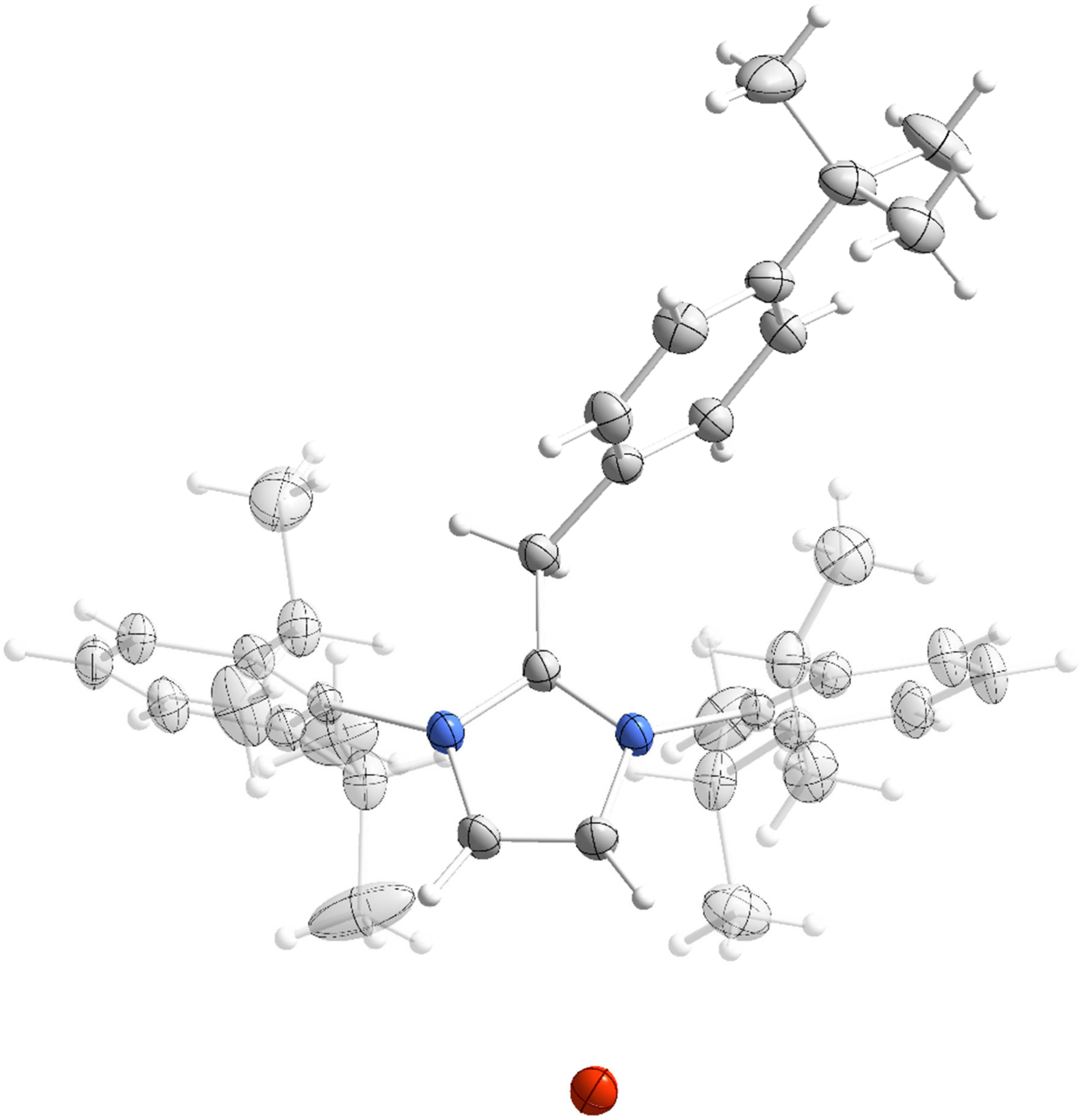

*Figure S10 1: Asymmetric unit of **6**. Solvent (MeCN) was omitted for clarity*

*Table S10 3: Bond lengths of **6***

N1—C2	1.344 (2)	C24—H24	0.9800
N1—C5	1.389 (2)	C24—C25	1.527 (3)
N1—C6	1.455 (2)	C24—C26	1.536 (3)
C2—N3	1.342 (2)	C25—H25A	0.9600
C2—C30	1.489 (2)	C25—H25B	0.9600
N3—C4	1.386 (2)	C25—H25C	0.9600
N3—C18	1.4569 (19)	C26—H26A	0.9600
C4—H4	0.9300	C26—H26B	0.9600
C4—C5	1.341 (2)	C26—H26C	0.9600
C5—H5	0.9300	C27—H27	0.9800
C6—C7	1.400 (2)	C27—C28	1.532 (3)
C6—C11	1.398 (2)	C27—C29	1.522 (3)
C7—C8	1.397 (2)	C28—H28A	0.9600
C7—C15	1.513 (3)	C28—H28B	0.9600
C8—H8	0.9300	C28—H28C	0.9600
C8—C9	1.374 (3)	C29—H29A	0.9600
C9—H9	0.9300	C29—H29B	0.9600
C9—C10	1.387 (3)	C29—H29C	0.9600
C10—H10	0.9300	C30—H30A	0.9700
C10—C11	1.391 (3)	C30—H30B	0.9700
C11—C12	1.524 (2)	C30—C31	1.516 (2)
C12—H12	0.9800	C31—C32	1.386 (2)
C12—C13	1.512 (3)	C31—C36	1.389 (2)
C12—C14	1.500 (3)	C32—H32	0.9300
C13—H13A	0.9600	C32—C33	1.389 (2)
C13—H13B	0.9600	C33—H33	0.9300
C13—H13C	0.9600	C33—C34	1.388 (2)
C14—H14A	0.9600	C34—C35	1.400 (3)
C14—H14B	0.9600	C34—C37	1.534 (2)
C14—H14C	0.9600	C35—H35	0.9300
C15—H15	0.9800	C35—C36	1.384 (3)
C15—C16	1.527 (3)	C36—H36	0.9300
C15—C17	1.530 (3)	C37—C38	1.539 (3)
C16—H16A	0.9600	C37—C39	1.528 (3)
C16—H16B	0.9600	C37—C40	1.527 (3)
C16—H16C	0.9600	C38—H38A	0.9600
C17—H17A	0.9600	C38—H38B	0.9600
C17—H17B	0.9600	C38—H38C	0.9600
C17—H17C	0.9600	C39—H39A	0.9600
C18—C19	1.397 (2)	C39—H39B	0.9600
C18—C23	1.395 (3)	C39—H39C	0.9600
C19—C20	1.396 (2)	C40—H40A	0.9600
C19—C27	1.520 (3)	C40—H40B	0.9600
C20—H20	0.9300	C40—H40C	0.9600
C20—C21	1.377 (3)	N4—C42	1.139 (4)
C21—H21	0.9300	C41—H41A	0.9600
C21—C22	1.376 (3)	C41—H41B	0.9600
C22—H22	0.9300	C41—H41C	0.9600
C22—C23	1.396 (2)	C41—C42	1.450 (4)
C23—C24	1.517 (3)		

*Table S10 4: Angles of **6***

C2—N1—C5	109.21 (13)	C23—C24—C26	110.80 (16)
C2—N1—C6	126.50 (13)	C25—C24—H24	108.0
C5—N1—C6	123.99 (13)	C25—C24—C26	109.52 (17)
N1—C2—C30	124.35 (14)	C26—C24—H24	108.0
N3—C2—N1	107.11 (13)	C24—C25—H25A	109.5
N3—C2—C30	128.49 (14)	C24—C25—H25B	109.5
C2—N3—C4	109.31 (13)	C24—C25—H25C	109.5
C2—N3—C18	126.76 (13)	H25A—C25—H25B	109.5
C4—N3—C18	123.43 (13)	H25A—C25—H25C	109.5
N3—C4—H4	126.4	H25B—C25—H25C	109.5
C5—C4—N3	107.29 (14)	C24—C26—H26A	109.5
C5—C4—H4	126.4	C24—C26—H26B	109.5
N1—C5—H5	126.5	C24—C26—H26C	109.5
C4—C5—N1	107.07 (14)	H26A—C26—H26B	109.5
C4—C5—H5	126.5	H26A—C26—H26C	109.5
C7—C6—N1	117.93 (15)	H26B—C26—H26C	109.5
C11—C6—N1	118.24 (14)	C19—C27—H27	107.4
C11—C6—C7	123.75 (15)	C19—C27—C28	110.11 (17)
C6—C7—C15	122.99 (16)	C19—C27—C29	113.67 (18)
C8—C7—C6	116.62 (17)	C28—C27—H27	107.4
C8—C7—C15	120.40 (16)	C29—C27—H27	107.4
C7—C8—H8	119.4	C29—C27—C28	110.50 (18)
C9—C8—C7	121.20 (16)	C27—C28—H28A	109.5
C9—C8—H8	119.4	C27—C28—H28B	109.5
C8—C9—H9	119.7	C27—C28—H28C	109.5
C8—C9—C10	120.53 (17)	H28A—C28—H28B	109.5
C10—C9—H9	119.7	H28A—C28—H28C	109.5
C9—C10—H10	119.4	H28B—C28—H28C	109.5
C9—C10—C11	121.14 (18)	C27—C29—H29A	109.5
C11—C10—H10	119.4	C27—C29—H29B	109.5
C6—C11—C12	122.51 (15)	C27—C29—H29C	109.5
C10—C11—C6	116.71 (16)	H29A—C29—H29B	109.5
C10—C11—C12	120.76 (17)	H29A—C29—H29C	109.5
C11—C12—H12	107.1	H29B—C29—H29C	109.5
C13—C12—C11	114.07 (17)	C2—C30—H30A	108.4
C13—C12—H12	107.1	C2—C30—H30B	108.4
C14—C12—C11	110.79 (17)	C2—C30—C31	115.64 (14)
C14—C12—H12	107.1	H30A—C30—H30B	107.4
C14—C12—C13	110.3 (2)	C31—C30—H30A	108.4
C12—C13—H13A	109.5	C31—C30—H30B	108.4
C12—C13—H13B	109.5	C32—C31—C30	119.73 (15)
C12—C13—H13C	109.5	C32—C31—C36	118.17 (16)
H13A—C13—H13B	109.5	C36—C31—C30	121.98 (15)
H13A—C13—H13C	109.5	C31—C32—H32	119.6
H13B—C13—H13C	109.5	C31—C32—C33	120.79 (16)
C12—C14—H14A	109.5	C33—C32—H32	119.6
C12—C14—H14B	109.5	C32—C33—H33	119.1
C12—C14—H14C	109.5	C34—C33—C32	121.78 (16)
H14A—C14—H14B	109.5	C34—C33—H33	119.1
H14A—C14—H14C	109.5	C33—C34—C35	116.79 (16)

H14B—C14—H14C	109.5	C33—C34—C37	122.76 (16)
C7—C15—H15	107.9	C35—C34—C37	120.45 (16)
C7—C15—C16	111.02 (17)	C34—C35—H35	119.2
C7—C15—C17	111.01 (18)	C36—C35—C34	121.68 (16)
C16—C15—H15	107.9	C36—C35—H35	119.2
C16—C15—C17	111.1 (2)	C31—C36—H36	119.6
C17—C15—H15	107.9	C35—C36—C31	120.78 (16)
C15—C16—H16A	109.5	C35—C36—H36	119.6
C15—C16—H16B	109.5	C34—C37—C38	111.83 (15)
C15—C16—H16C	109.5	C39—C37—C34	109.06 (17)
H16A—C16—H16B	109.5	C39—C37—C38	108.92 (19)
H16A—C16—H16C	109.5	C40—C37—C34	109.63 (15)
H16B—C16—H16C	109.5	C40—C37—C38	107.84 (18)
C15—C17—H17A	109.5	C40—C37—C39	109.54 (18)
C15—C17—H17B	109.5	C37—C38—H38A	109.5
C15—C17—H17C	109.5	C37—C38—H38B	109.5
H17A—C17—H17B	109.5	C37—C38—H38C	109.5
H17A—C17—H17C	109.5	H38A—C38—H38B	109.5
H17B—C17—H17C	109.5	H38A—C38—H38C	109.5
C19—C18—N3	117.24 (15)	H38B—C38—H38C	109.5
C23—C18—N3	118.66 (14)	C37—C39—H39A	109.5
C23—C18—C19	123.99 (15)	C37—C39—H39B	109.5
C18—C19—C27	122.83 (15)	C37—C39—H39C	109.5
C20—C19—C18	116.61 (17)	H39A—C39—H39B	109.5
C20—C19—C27	120.40 (16)	H39A—C39—H39C	109.5
C19—C20—H20	119.4	H39B—C39—H39C	109.5
C21—C20—C19	121.21 (18)	C37—C40—H40A	109.5
C21—C20—H20	119.4	C37—C40—H40B	109.5
C20—C21—H21	119.9	C37—C40—H40C	109.5
C22—C21—C20	120.22 (17)	H40A—C40—H40B	109.5
C22—C21—H21	119.9	H40A—C40—H40C	109.5
C21—C22—H22	119.1	H40B—C40—H40C	109.5
C21—C22—C23	121.76 (19)	H41A—C41—H41B	109.5
C23—C22—H22	119.1	H41A—C41—H41C	109.5
C18—C23—C22	116.18 (17)	H41B—C41—H41C	109.5
C18—C23—C24	122.72 (15)	C42—C41—H41A	109.5
C22—C23—C24	121.06 (17)	C42—C41—H41B	109.5
C23—C24—H24	108.0	C42—C41—H41C	109.5
C23—C24—C25	112.46 (17)	N4—C42—C41	179.1 (3)

S11 Synthesis and characterization of other products (14 - 17)

Salts **14**, **15**, and **16** of **4a** with amines, namely pyridine (py), trimethylamine (TEA) and *N,N*–diisopropylethylamine (DIPEA) respectively, were synthesized separately in order to compare the reactivity of different amines with the benzylic substrates.

It can be seen from the yields that out of the three used amines, the most sterically hindered DIPEA with **4a** affords the smallest amount of salt **16** (26 %). Salt **17** of **4c** with DIPEA is formed in even smaller amounts (11%).

(14) 1-(4-(*tert*-butyl)benzyl)pyridinium bromide

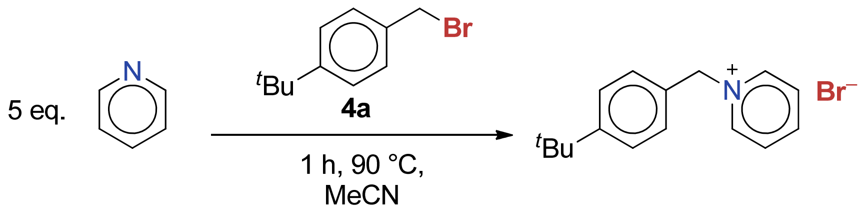

14, $C_{16}H_{20}NBr$, 83 %

*Scheme S11 1: Synthesis of **14***

A screw cap vial was charged with 81 mg of **4a** (0.4 mmol), 150 µL of pyridine and 0.5 ml of acetonitrile. Reaction mixture was heated to 90 °C. Reaction progress was monitored with TLC. After 1 hour the reaction mixture was left to cool to room temperature. Reaction solvent and excess of pyridine were removed under reduced pressure. Oily residue was treated with a few ml of diethyl ether. Resulting white precipitate was filtered off to give **15** in 83 % yield. Product structure was determined using 1D and 2D NMR spectroscopy. Spectroscopic data of isolated **15** can be found on Page 57.

^{1}H NMR (29 °C, $CDCl_3$, 300.13 MHz): δ = 9.62 (d, *J* = 6 Hz, 2H), 8.47 – 8.42 (m, 1H), 8.07 – 8. (m, 2H), 7.64 (d, *J* = 8 Hz, 2H), 7.39 (d, *J* = 8 Hz, 2H), 6.29 (s, 2H), 1.27 (s, 9H).

^{13}C NMR (29 °C, $CDCl_3$, 75.47 MHz): δ = 153.4 (s, C_c), 145.2 (s, 2C, C_h), 145.1 (s, 2C, C_i), 129.9 (s, C_f), 129.6 (s, 2C, C_d), 128.3 (s, C_j), 126.7 (s, 2C, C_e), 64.1 (s, C_g), 34.8 (s, C_b), 31.3 (s, 3C, C_a). For carbon spectrum assignation and lettering please refer to the attached physical copy on Page 57.

HRMS (ESI+): calculated for $C_{16}H_{20}N^+$: 226.1590, found: 226.1603.

(15) (4-(*tert*-butyl)benzyl)triethylammonium bromide

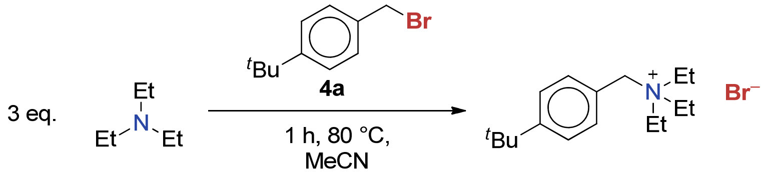

15, $C_{17}H_{30}NBr$, 61 %

Scheme S11 2: Synthesis of 15

A screw cap vial was charged with 82 mg of **4a** (0.4 mmol), 150 µL of triethylamine and 0.5 ml of acetonitrile. Reaction mixture was heated to 80 °C. After 1 hour the reaction mixture was left to cool to room temperature. Solvent and excess of triethylamine were removed under reduced pressure. Oily residue was treated with a few ml of EtOAc, from which it crystalized overnight. Resulting hygroscopic white precipitate was filtered off to give **15** in 61 % yield. Product structure was determined using 1D and 2D NMR spectroscopy. Spectroscopic data of isolated **15** can be found on Page 59.

^{1}H NMR (29 °C, $CDCl_3$, 300.13 MHz): δ = 7.51–7.41 (m, 4H), 4.71 (s, 2H), 3.45 (q, *J* = 7 Hz, 6H), 1.48 (q, *J* = 8 Hz, 9H), 1.32 (s, 9H).

^{13}C NMR (23 °C, $CDCl_3$, 125.76 MHz): δ = 154.3 (s, C_c), 132.3 (s, 2C, C_e), 126.6 (s, 2C, C_d), 123.9 (s, C_f), 61.1 (s, C_g), 53.0 (s, 3C, C_h), 35.0 (s, C_b), 31.2 (s, 3C, C_a), 8.7 (s, 3C, C_i). For carbon spectrum assignation and lettering please refer to the attached physical copy on Page 59.

HRMS (ESI+): calculated for $C_{17}H_{30}N^+$: 248.2737, found: 248.2730.

(16) (4-(*tert*-butyl)benzyl)ethyldiisopropylammonium bromide

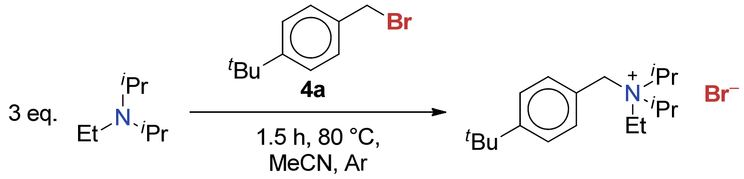

16, $C_{19}H_{34}NBr$, 26 %

Scheme S11 3: Synthesis of 16

A screw cap vial was charged with 82 mg of **4a** (0.4 mmol), 200 µL of DIPEA and 0.5 ml of acetonitrile. Reaction mixture was purged with argon and heated to 80 °C. After 1.5 hours the reaction mixture was left to cool to room temperature. Solvent and excess of DIPEA were removed under reduced pressure. Oily residue was treated with a few ml of EtOAc and heated with a heat gun. During this process oily residue turned into a white precipitate, which was subsequently filtered off to give **16** in 26 % yield. Product structure was determined using 1D and 2D NMR spectroscopy. Spectroscopic data of isolated **16** can be found on Page 61.

^{1}H NMR (29 °C, $CDCl_3$, 300.13 MHz): δ = 7.70 (d, *J* = 8 Hz, 2H), 7.43 (d, *J* = 8 Hz, 2H), 4.67 (s, 2H), 4.30 (sept, *J* = 6 Hz, 2H), 3.65 (q, *J* = 7 Hz, 2H), 1.56 (d, *J* = 6 Hz, 6H), 1.44 (m, 9H), 1.30 (s, 9H).

^{13}C NMR (29 °C, $CDCl_3$, 75.47 MHz): δ = 154.0 (s, C_c), 133.0 (s, 2C, C_e), 126.3 (s, 2C, C_d), 125.6 (s, C_f), 62.5 (s, C_h), 62.0 (s, C_g), 51.4 (s, C_j), 34.9 (s, C_b), 31.2 (s, 3C, C_a), 19.7 (s, 2C, C_i), 19.4 (s, 2C, $C_{i'}$), 10.4 (s, C_k). For carbon spectrum assignation and lettering please refer to the attached physical copy on Page 61.

HRMS (ESI+): calculated for $C_{19}H_{34}N^+$: 276.2686, found: 276.2685.

(17) (3-(pheny)lbenzyl)ethyldiisopropylammonium bromide

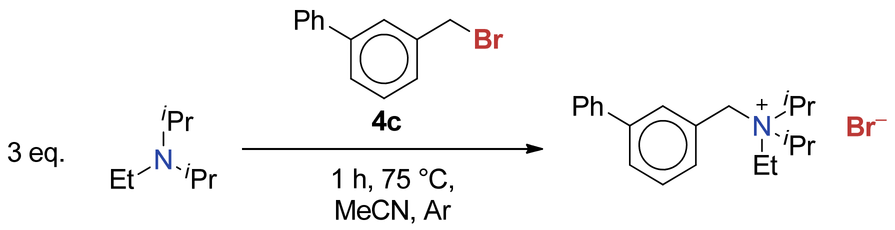

17, $C_{21}H_{30}NBr$, 11 %

Scheme S11 4: Synthesis of 17

A screw cap vial was charged with 123 mg of **4c** (0.5 mmol), 250 μL of DIPEA and 0.5 ml of acetonitrile. Reaction mixture was purged with argon and heated to 75 °C. After 1 hour the reaction mixture was left to cool to room temperature. Solvent and excess of DIPEA were removed under reduced pressure. Oily residue was treated with a few ml of EtOAc. Resulting white precipitate was filtered off to give **17** in 11 % yield. Product structure was determined using 1D and 2D NMR spectroscopy. Spectroscopic data of isolated **17** can be found on Page 63.

^{1}H NMR (29 °C, $CDCl_3$, 300.13 MHz): δ = 8.01 (s, 1H), 7.82 (d, *J* = 8 Hz, 1H), 7.70–7.62 (m, 3H), 7.51–7.41 (m, 3H), 7.40–7.32 (m, 1H), 4.85 (s, 2H), 4.34 (sept, *J* = 7 Hz, 2H), 3.69 (q, *J* = 7 Hz, 2H), 1.55 (d, *J* = 7 Hz, 6H), 1.49–1.37 (m, 9H).

^{13}C NMR (29 °C, $CDCl_3$, 75.47 MHz): δ = 142.0 (s), 139.6 (s), 132.4 (s), 131.9 (s), 129.9 (s), 129.5 (s), 129.2 (s), 129.1 (s), 128.1 (s), 127.3 (s), 62.7 (s, N–*C*HMe$_2$), 62.4 (s, Ar–*C*H$_2$–N), 51.4 (s, N–*C*H$_2$Me), 19.8 (s, N–CH(*C*H$_3$)$_2$), 19.5 (s, N–CH(*C*H$_3$)$_2$), 10.4 (s, N–CH$_2$–*C*H$_3$).

HRMS (ESI+): calculated for $C_{21}H_{30}N^{+}$: 296.2373, found: 296.2374.

S12 Spectroscopic data of isolated compounds

Spectroscopic data of 1-(tert-butyl)-4-(fluoromethyl)benzene (5a)

is consistent with following literature.[20] Additional characterization data (HRMS and IR) can also be found therein. Details on synthesis procedure of **5a** can be found on Pages 16 and 18.

---

^{1}H NMR of **5a** ($CDCl_3$, 23 °C, 500.13 MHz)

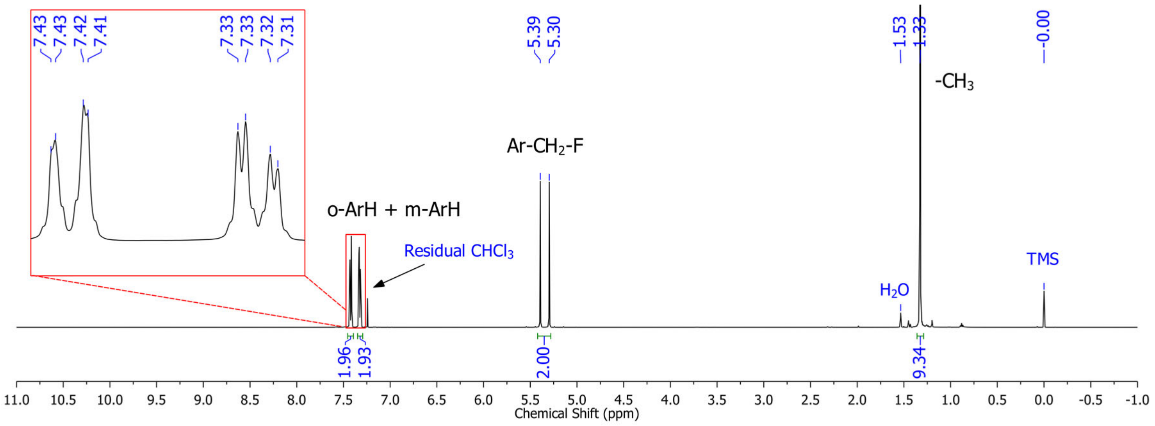

^{13}C NMR of **5a** ($CDCl_3$, 23 °C, 125.76 MHz)

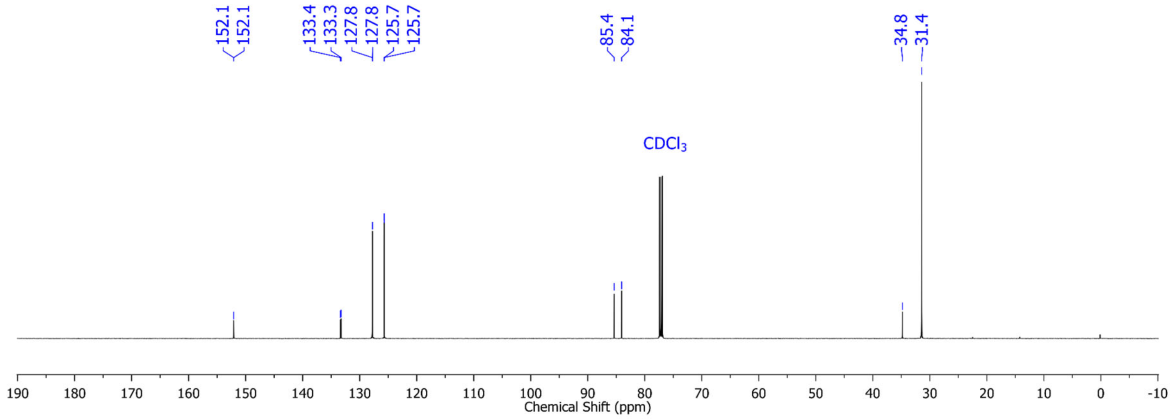

^{19}F NMR of **5a** ($CDCl_3$, 23 °C, 470.55 MHz)

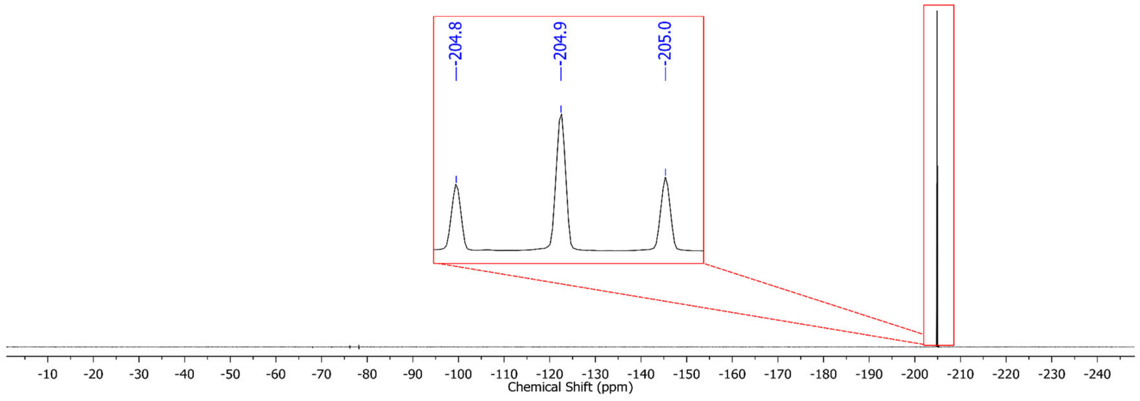

Spectroscopic data of 1-(fluoromethyl)-4-methylbenzene (**5b**)

is consistent with following literature.[21] Additional characterization data (IR) can also be found therein. Physical properties (melting point, boiling point, density and refractive index) have been reported.[44] Details on synthesis procedure of **5b** can be found on Pages 16 and 18.

^{1}H NMR of **5b** ($CDCl_3$, 23 °C, 500.13 MHz)

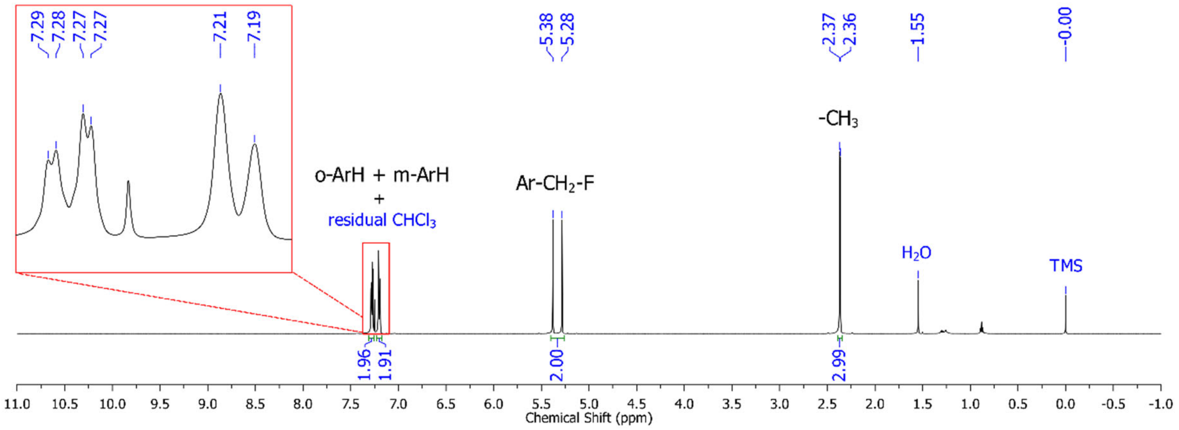

^{13}C NMR of **5b** ($CDCl_3$, 23 °C, 125.76 MHz)

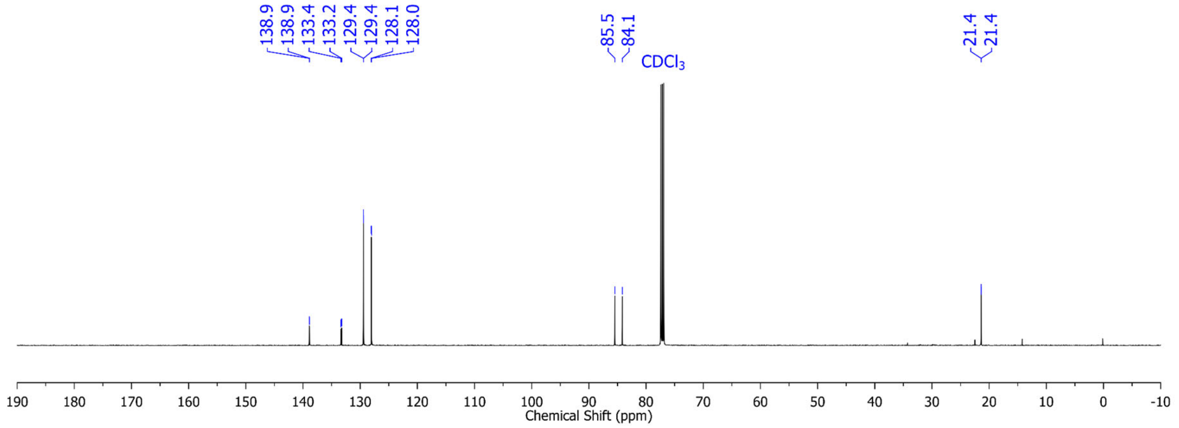

^{19}F NMR of **5b** ($CDCl_3$, 23 °C, 470.55 MHz)

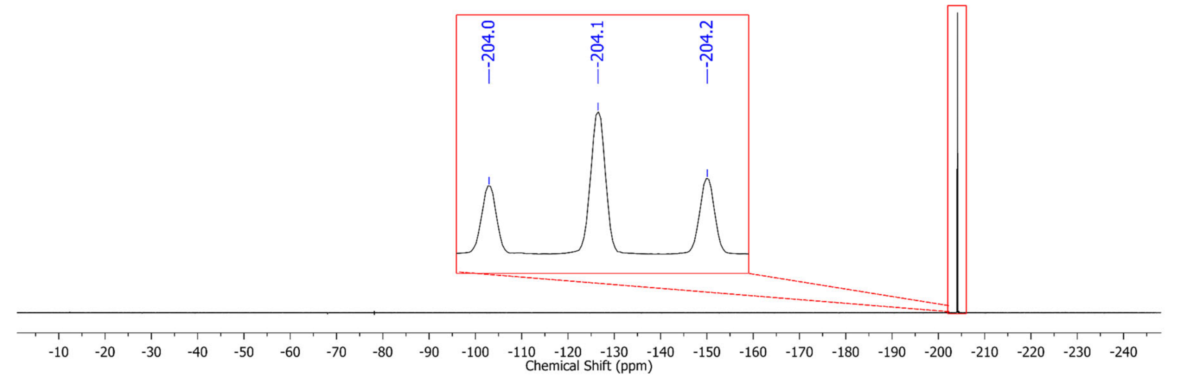

Spectroscopic data of 3-(fluoromethyl)-1,1'-biphenyl (**5c**)
is consistent with following literature.[22] Additional characterization data (MS, HRMS and IR) can also be found therein. Details on synthesis procedure of **5c** can be found on Pages 16 and 19.

^{1}H NMR of **5c** ($CDCl_3$, 23 °C, 500.13 MHz)

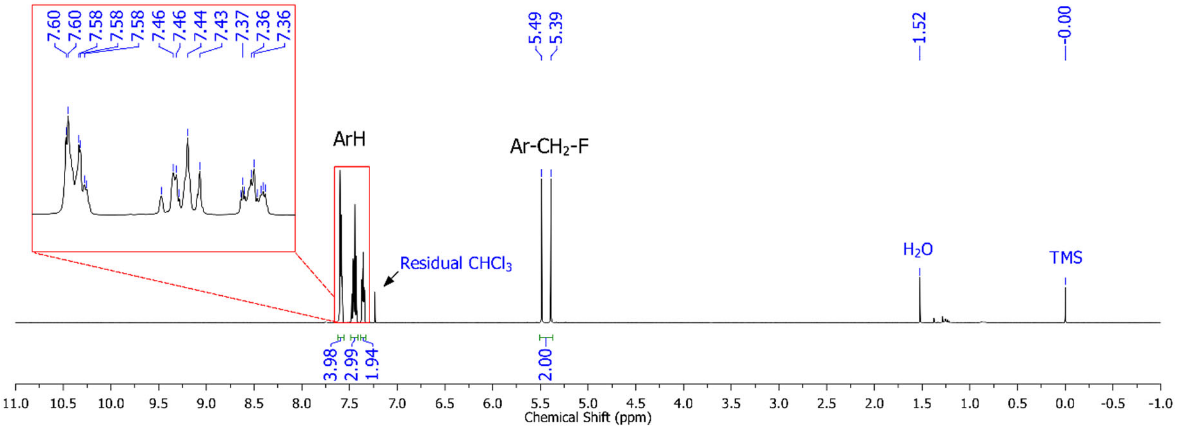

^{13}C NMR of **5c** ($CDCl_3$, 23 °C, 125.76 MHz)

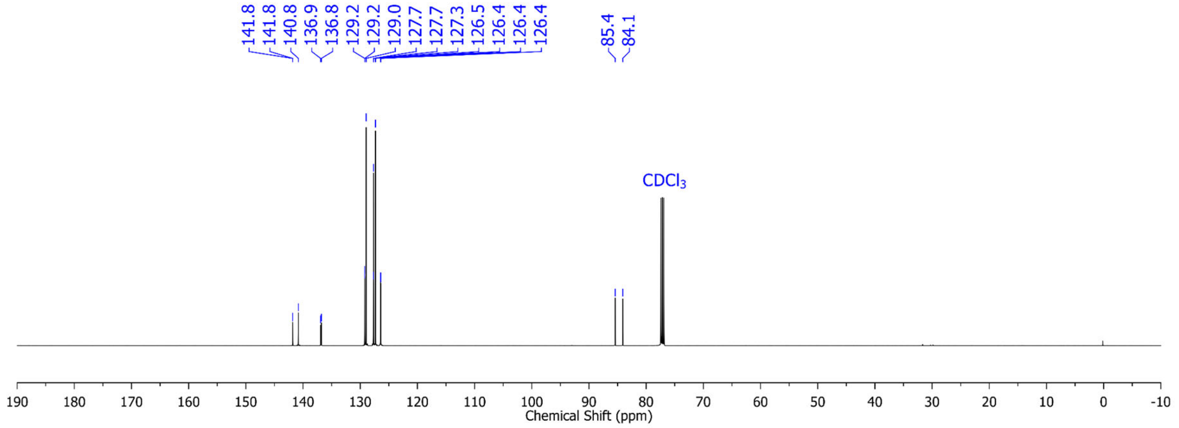

^{19}F NMR of **5c** ($CDCl_3$, 23 °C, 470.55 MHz)

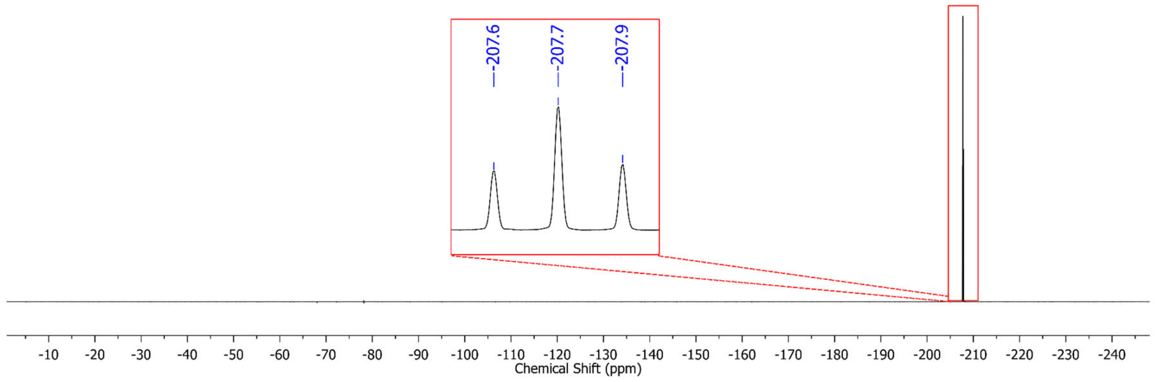

Spectroscopic data of 1-bromo-4-(fluoromethyl)benzene (**5d**)

is consistent with following literature.[23] Additional characterization data (IR) can also be found therein. Details on synthesis procedure of **5d** can be found on Pages 16 and 19.

---

^{1}H NMR of **5d** ($CDCl_3$, 29 °C, 300.13 MHz)

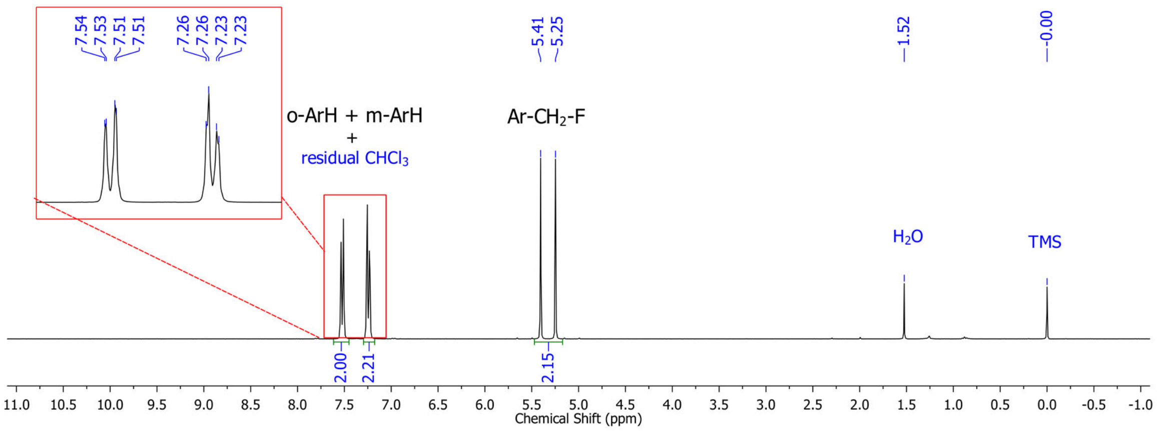

^{13}C NMR of **5d** ($CDCl_3$, 23 °C, 125.76 MHz)

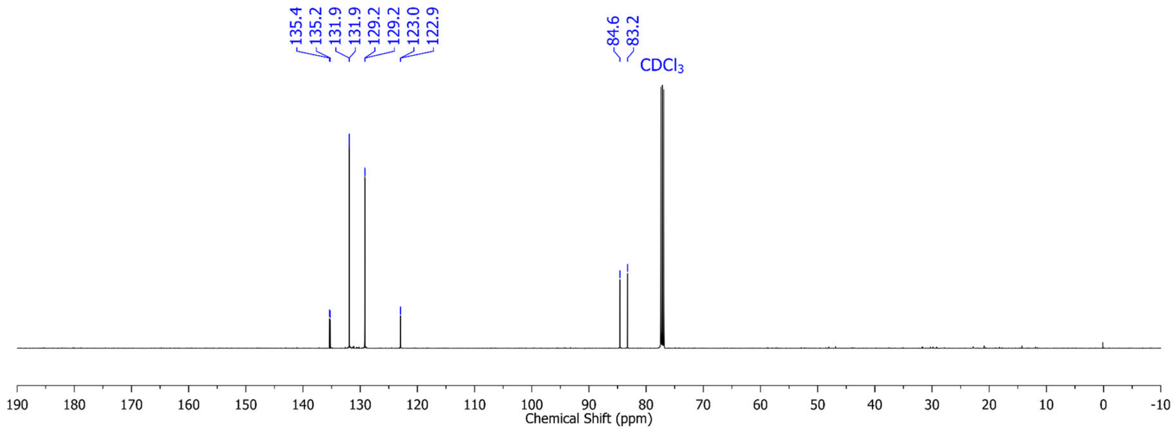

^{19}F NMR of **5d** ($CDCl_3$, 23 °C, 470.55 MHz)

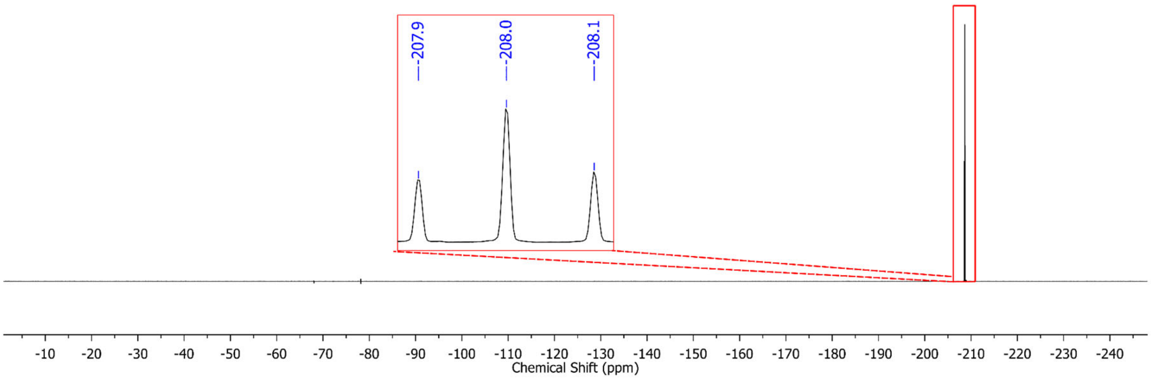

Spectroscopic data of ethyl (4-fluoromethyl)benzoate (**5g**) is consistent with following literature.[26] Additional characterization data (MS, HRMS and IR) can also be found therein. Details on synthesis procedure of **5g** can be found on Pages 16 and 20.

---

^{1}H NMR of **5g** ($CDCl_3$, 23 °C, 500.13 MHz)

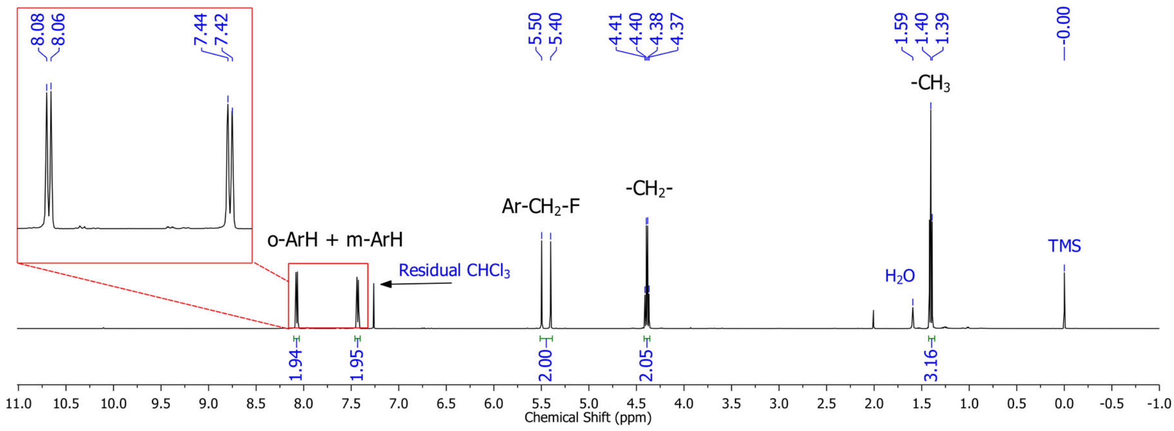

^{13}C NMR of **5g** ($CDCl_3$, 23 °C, 125.76 MHz)

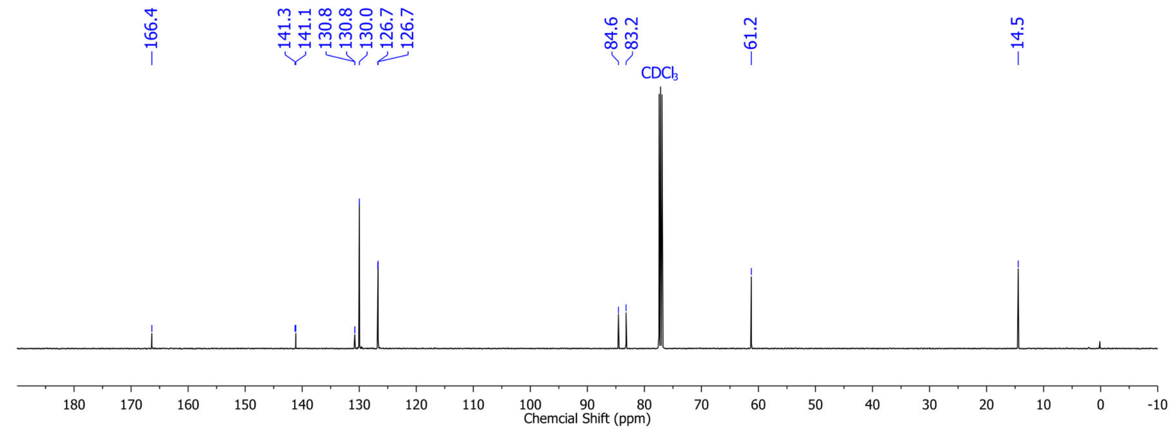

^{19}F NMR of **5g** ($CDCl_3$, 23 °C, 470.55 MHz)

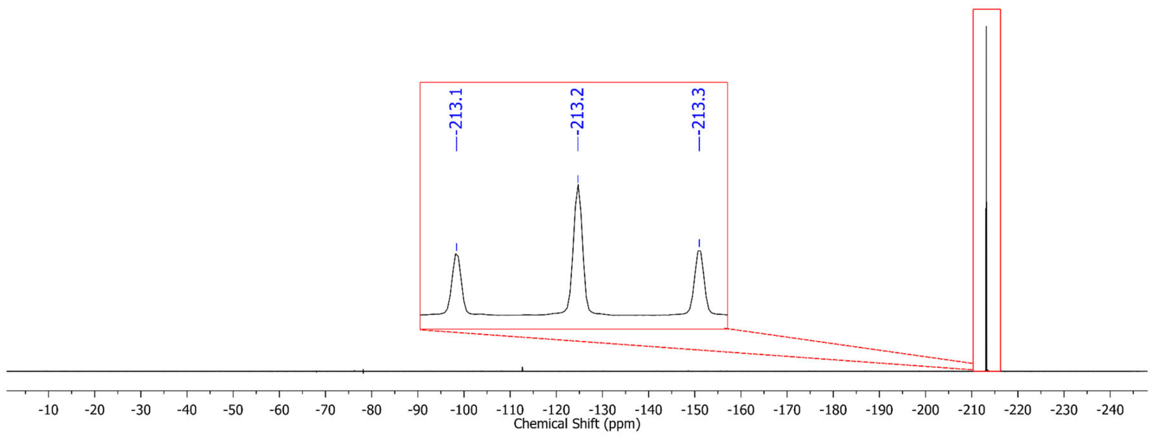

Spectroscopic data of 1-(fluoromethyl)-3,5-bis(trifluoromethyl)-benzene (**5h**) is consistent with proposed structure. Details on synthesis procedure of **5h** can be found on Pages 16 and 20.

---

^{1}H NMR of **5h** ($CDCl_3$, 23 °C, 500.13 MHz)

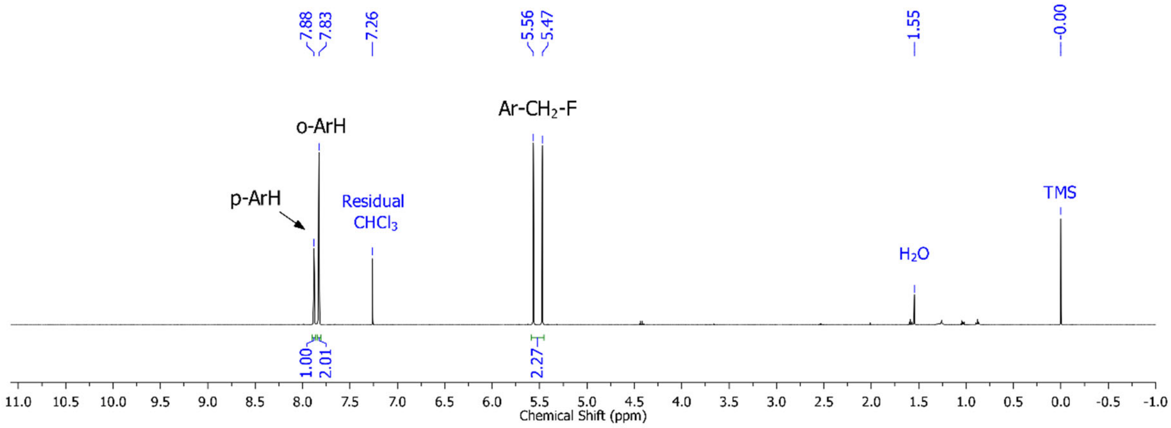

^{13}C NMR of **5h** ($CDCl_3$, 23 °C, 125.76 MHz)

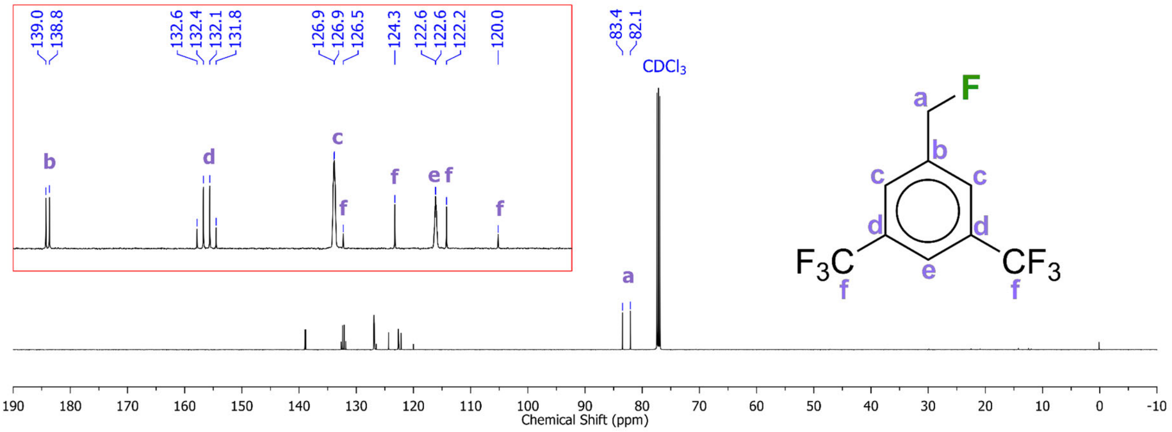

^{19}F NMR of **5h** ($CDCl_3$, 23 °C, 470.55 MHz)

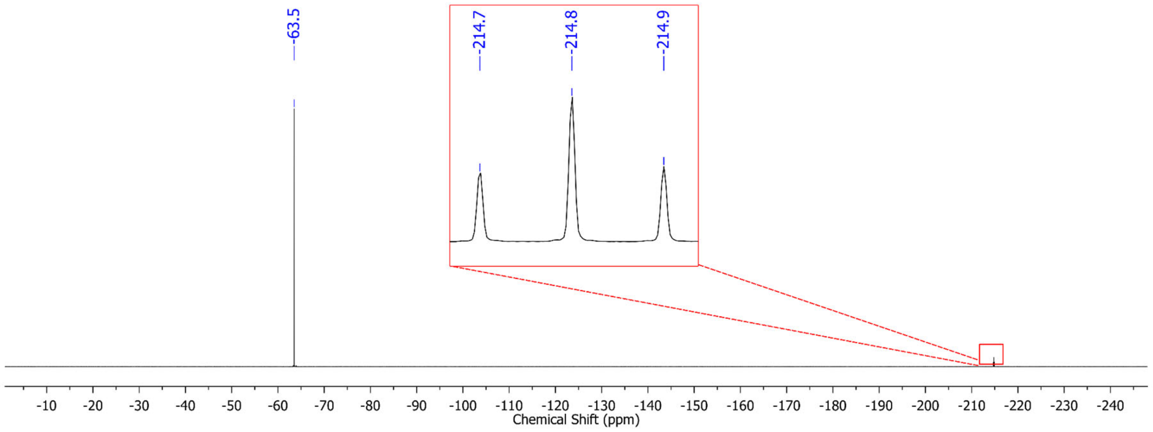

HSQC spectrum of **5h** (23 °C, $CDCl_3$)

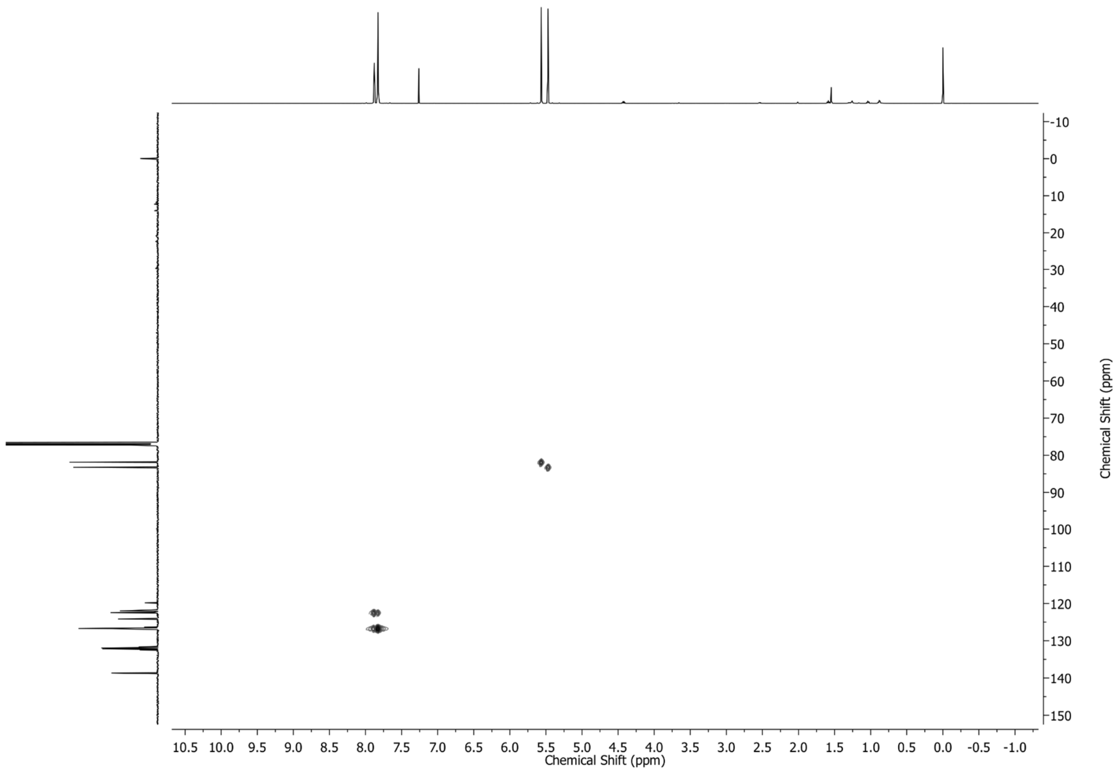

HMBC spectrum of **5h** (23 °C, $CDCl_3$)

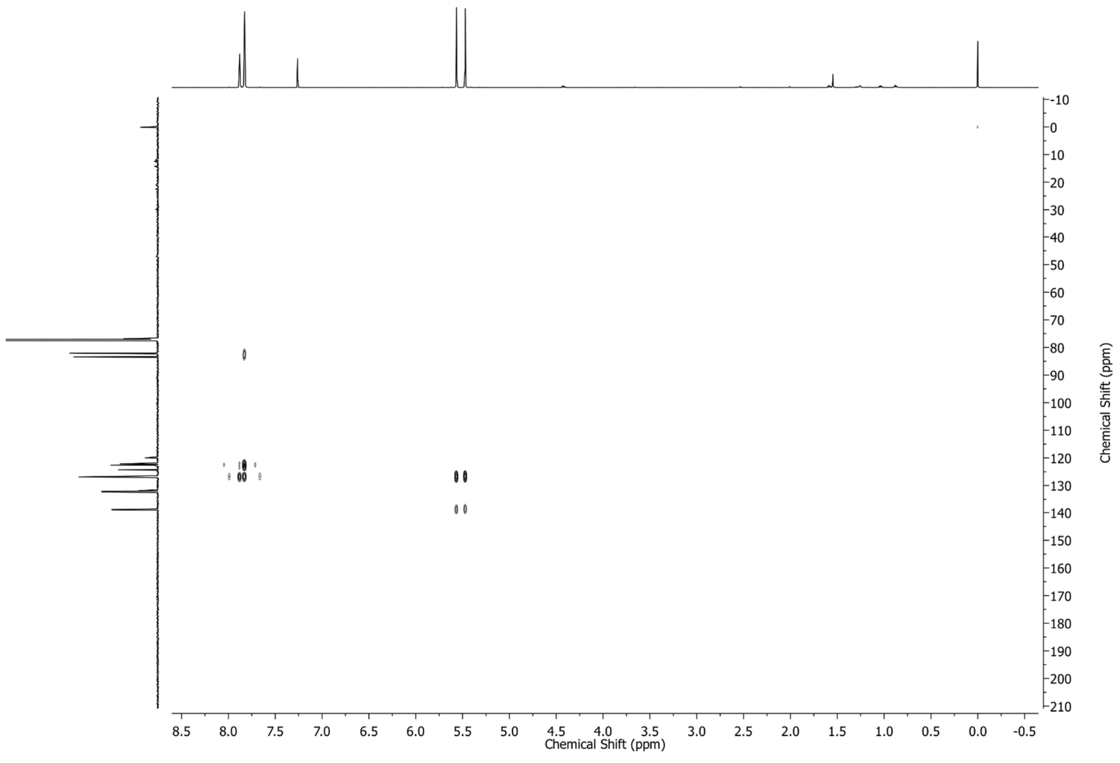

Spectroscopic data of 1-(fluoromethyl)-4-nitrobenzene (5i)

is consistent with following literature.[27] Additional characterization data (melting point, IR) can be found here.[45] Details on synthesis procedure of **5i** can be found on Pages 16 and 20.

^{1}H NMR of **5i** ($CDCl_3$, 29 °C, 300.13 MHz)

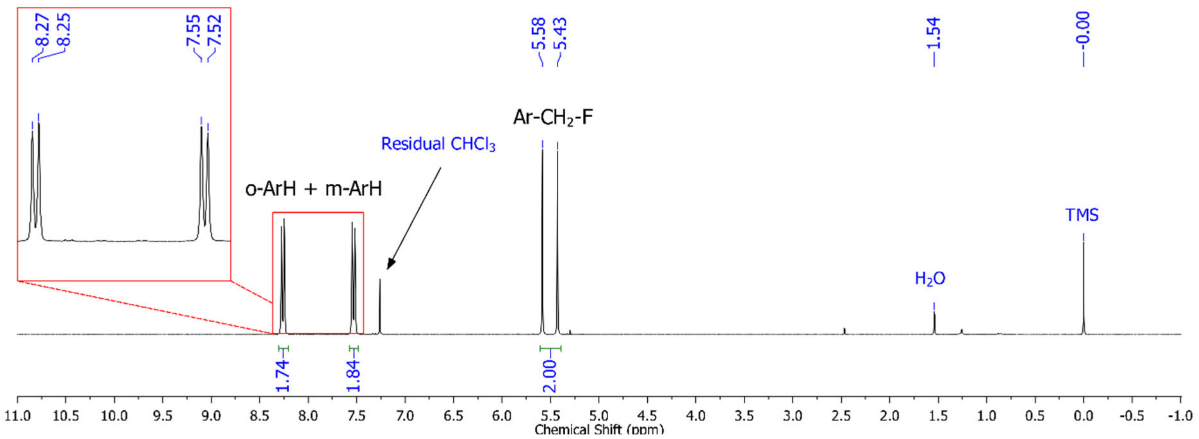

^{13}C NMR of **5i** ($CDCl_3$, 23 °C, 125.76 MHz)

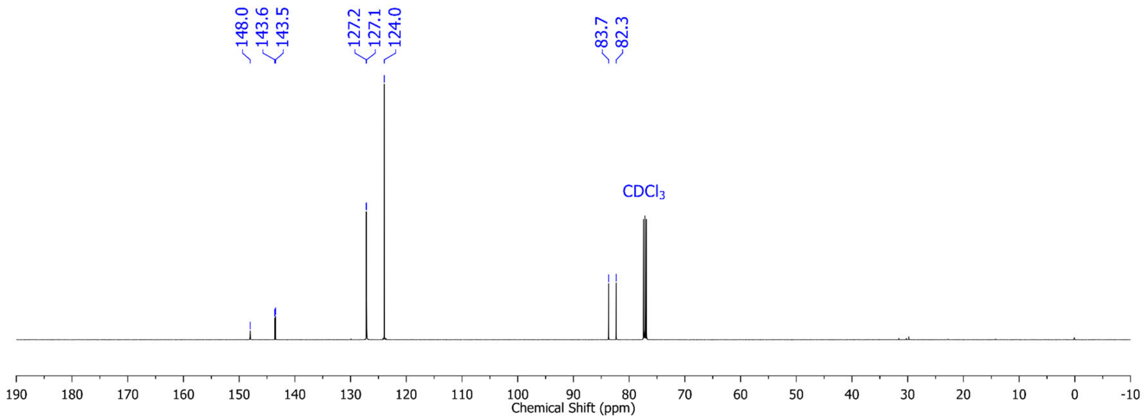

^{19}F NMR of **5i** ($CDCl_3$, 23 °C, 470.55 MHz)

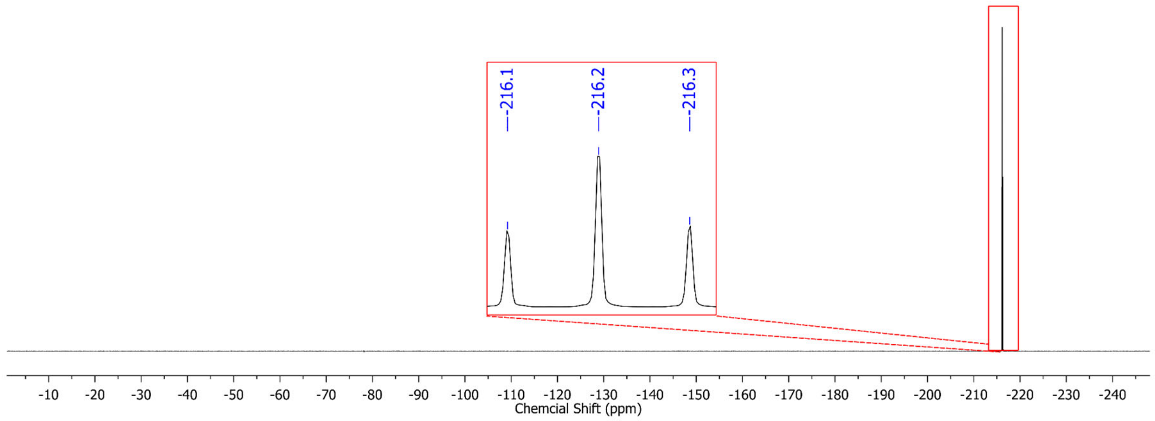

Spectroscopic data of 3α-fluoro-5α-cholestane (8b)

is consistent with following literature.[33] Additional characterization data (MS and melting point) can be found here.[46] Details on synthesis procedure of **8b** can be found on Page 22.

---

^{1}H NMR of **8b** ($CDCl_3$, 29 °C, 300.13 MHz)

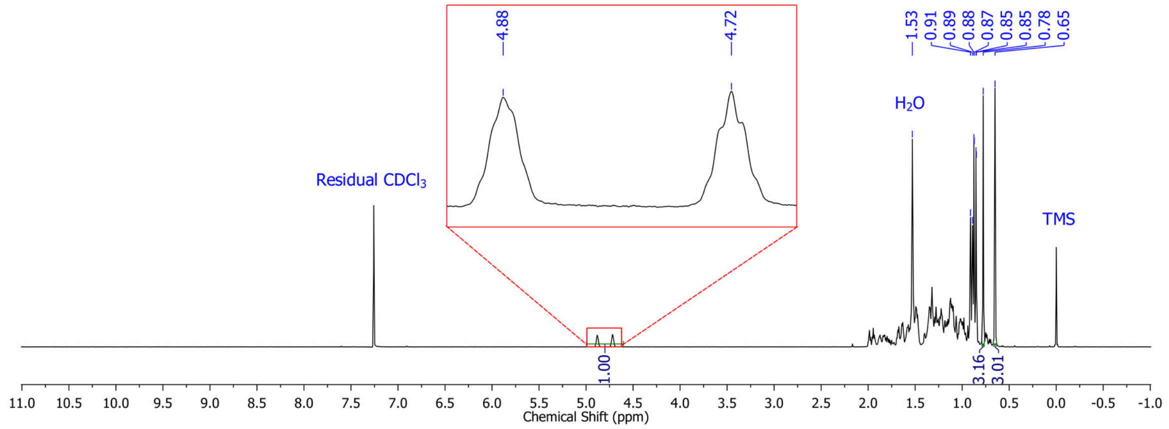

^{13}C NMR of **8b** ($CDCl_3$, 23 °C, 125.76 MHz)

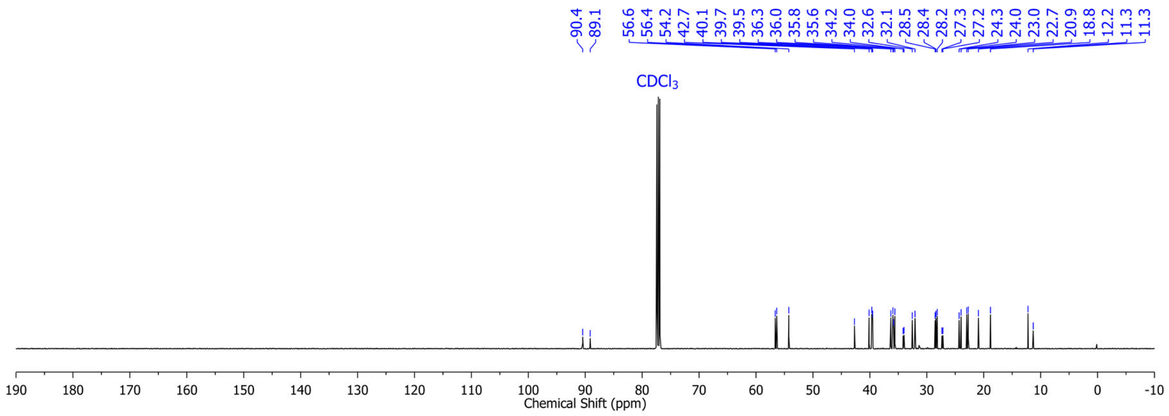

^{19}F NMR of **8b** ($CDCl_3$, 23 °C, 470.55 MHz)

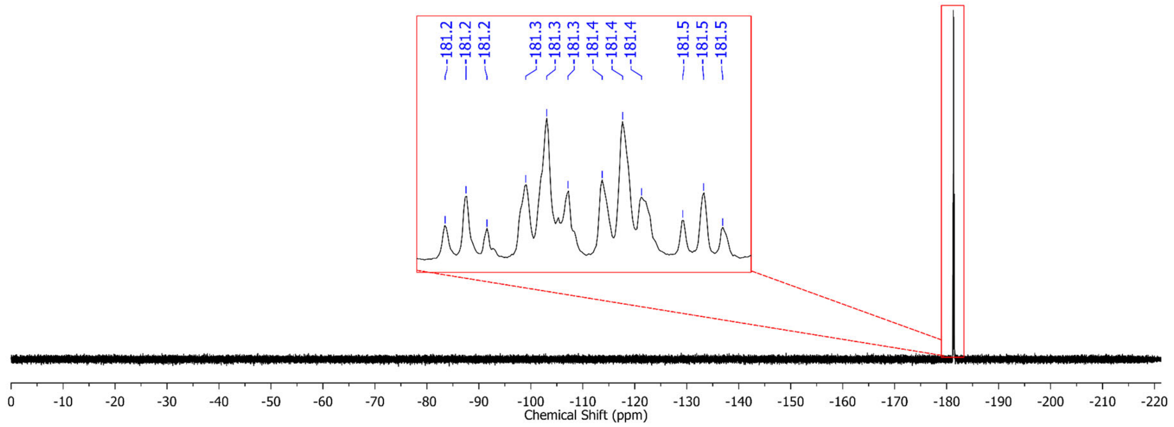

Spectroscopic data of 4-nitrobenzoyl fluoride (8g)

is consistent with following literature.[30] Additional characterization data (MS and IR) can also be found therein. Details on synthesis procedure of **8g** can be found on Page 24.

---

^1H NMR of **8g** ($CDCl_3$, 29 °C, 300.13 MHz)

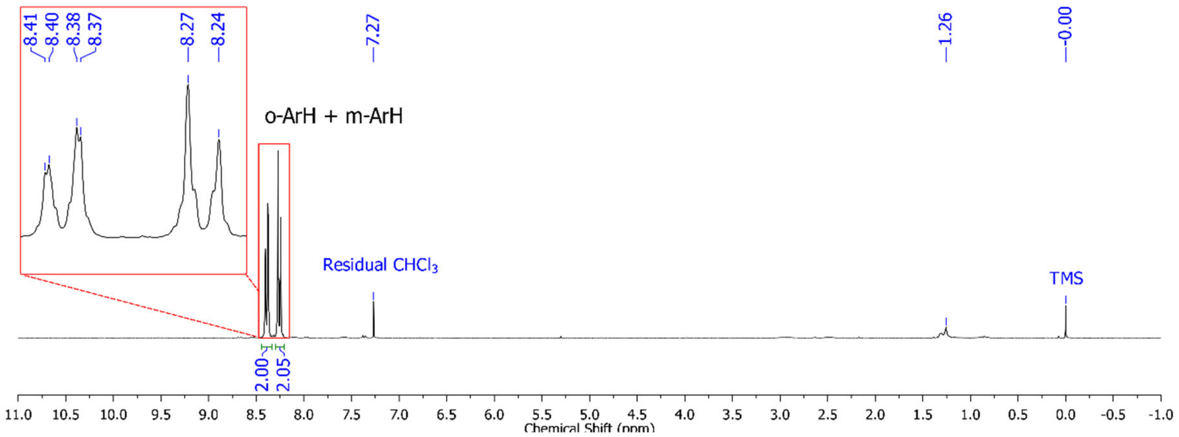

^{13}C NMR of **8g** ($CDCl_3$, 29 °C, 75.47 MHz)

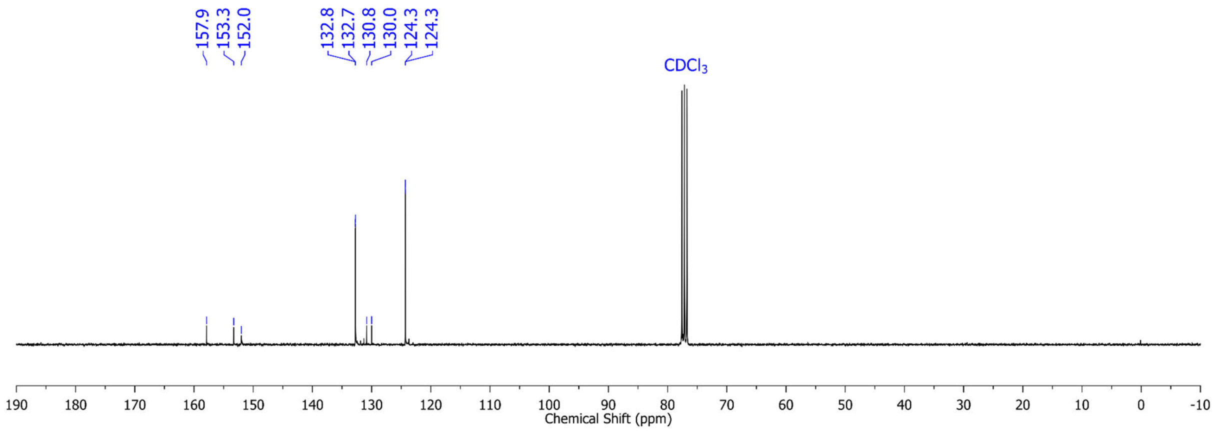

^{19}F NMR of **8g** ($CDCl_3$, 23 °C, 470.55 MHz)

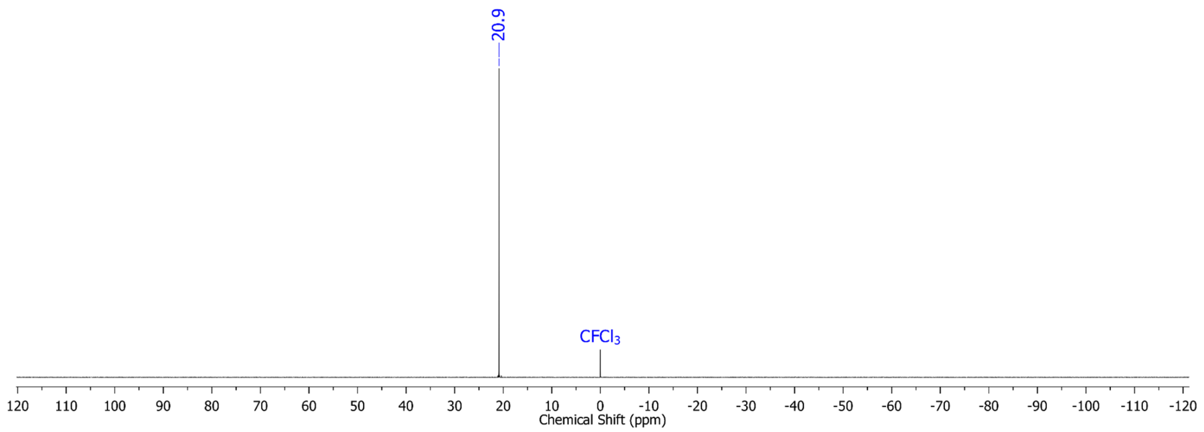

Spectroscopic data of 4-methylbenzenesulfonyl fluoride (8h)

is consistent with following literature.[39] Details on synthesis procedure of **8h** can be found on Page 24.

---

^{1}H NMR of **8h** ($CDCl_3$, 29 °C, 300.13 MHz)

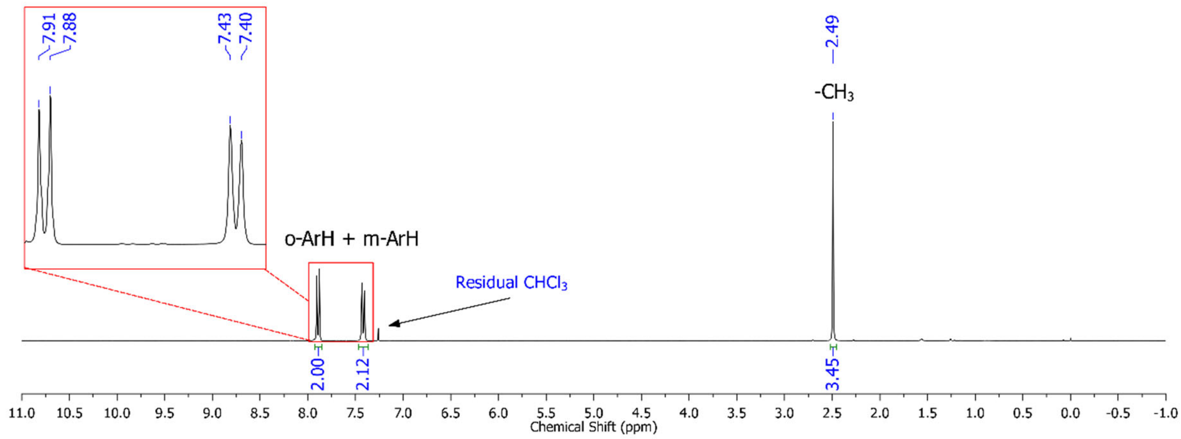

^{13}C NMR of **8h** ($CDCl_3$, 29 °C, 75.47 MHz)

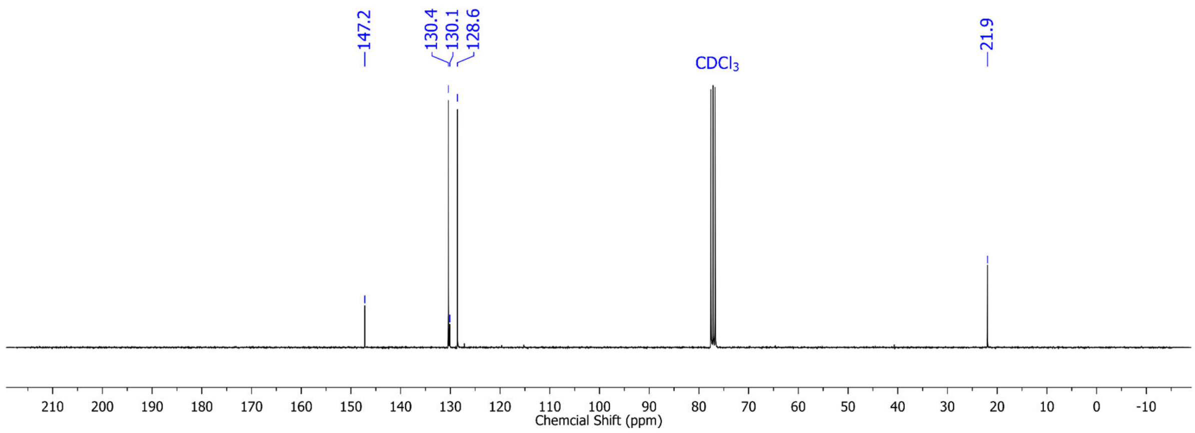

^{19}F NMR of **8h** ($CDCl_3$, 23 °C, 470.55 MHz)

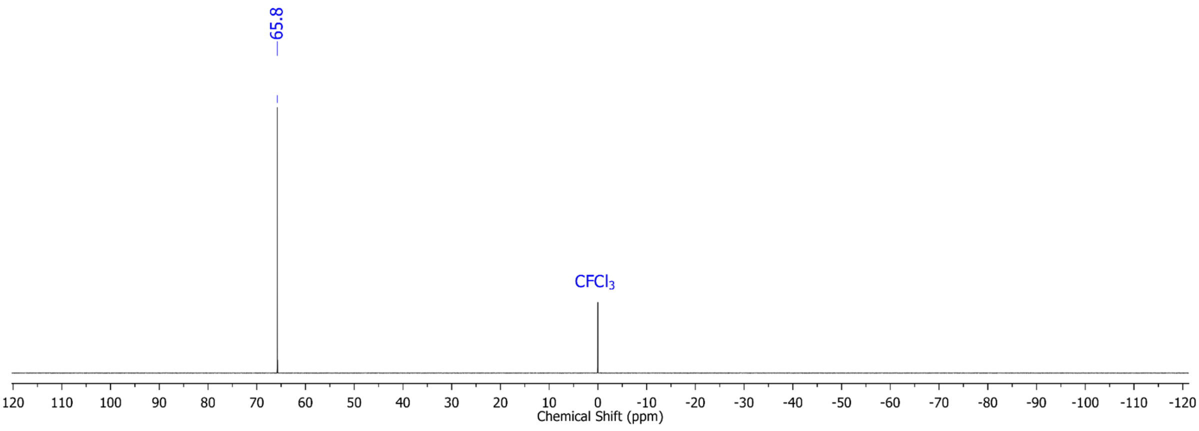

Spectroscopic data of 2-(4-(tert-butyl)benzyl)-1,3-bis(2,6-diisopropylphenyl)-1H-imidazol-3-ium bromide (6)

is consistent with the proposed structure. Details on synthesis procedure of **6** can be found on Page 35.

---

^{1}H NMR of **6** (CD_3CN, 29 °C, 300.13 MHz)

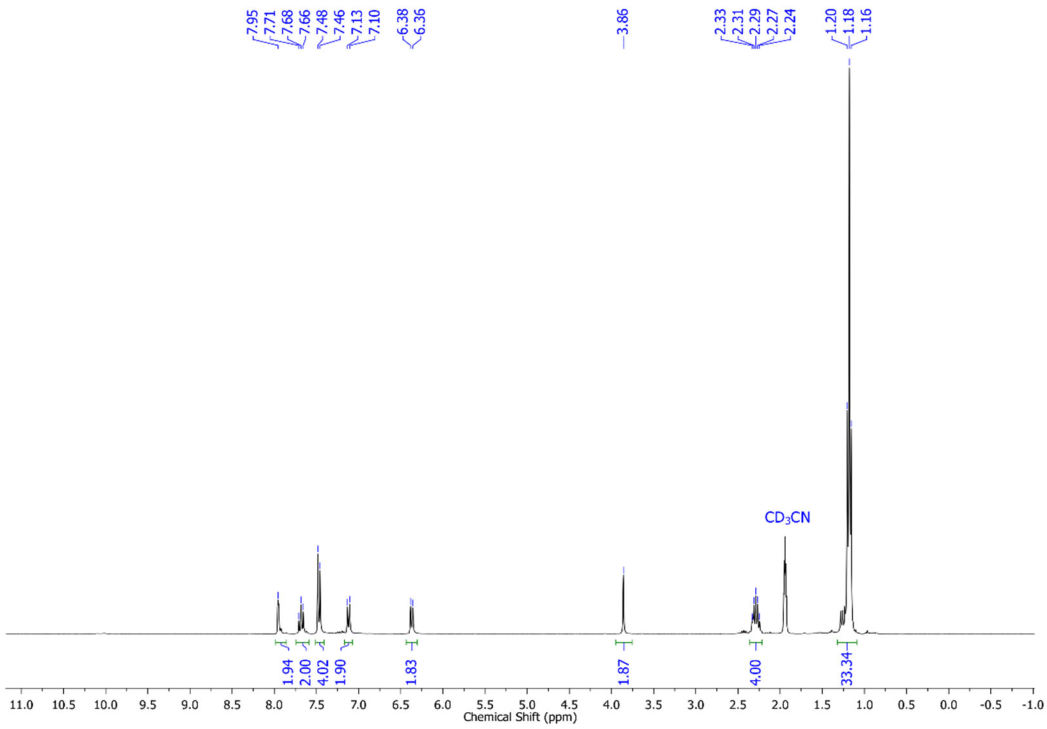

^{13}C NMR of **6** (CD_3CN, 29 °C, 75.47 MHz)

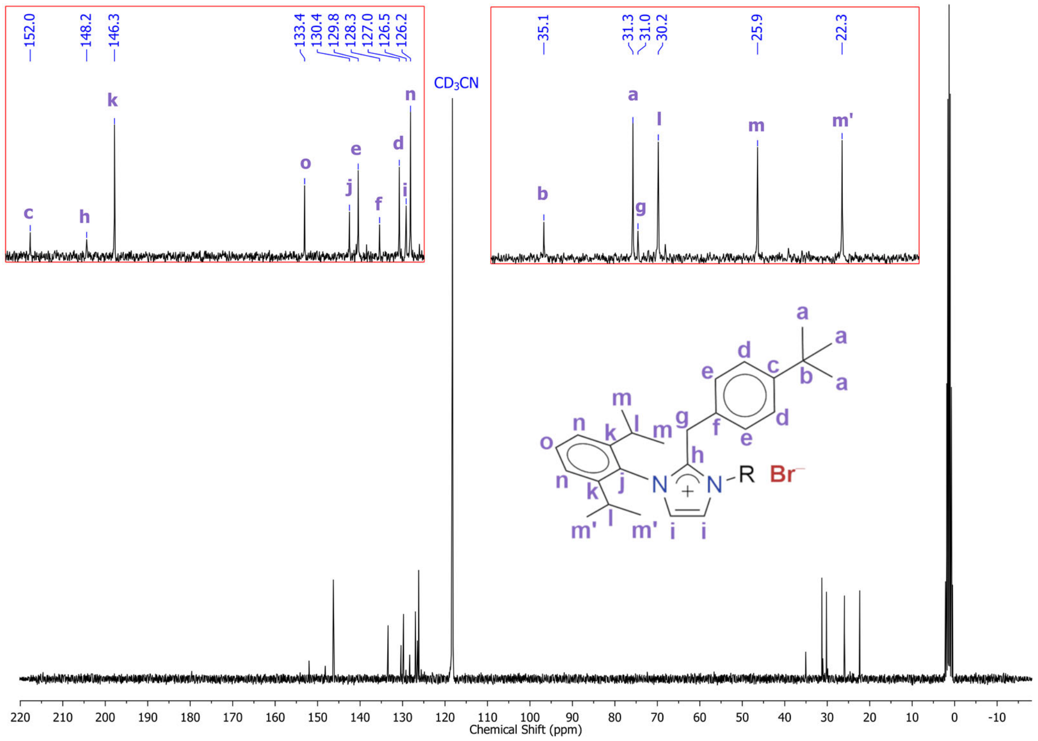

HSQC spectrum of **6** (23 °C, CD_3CN)

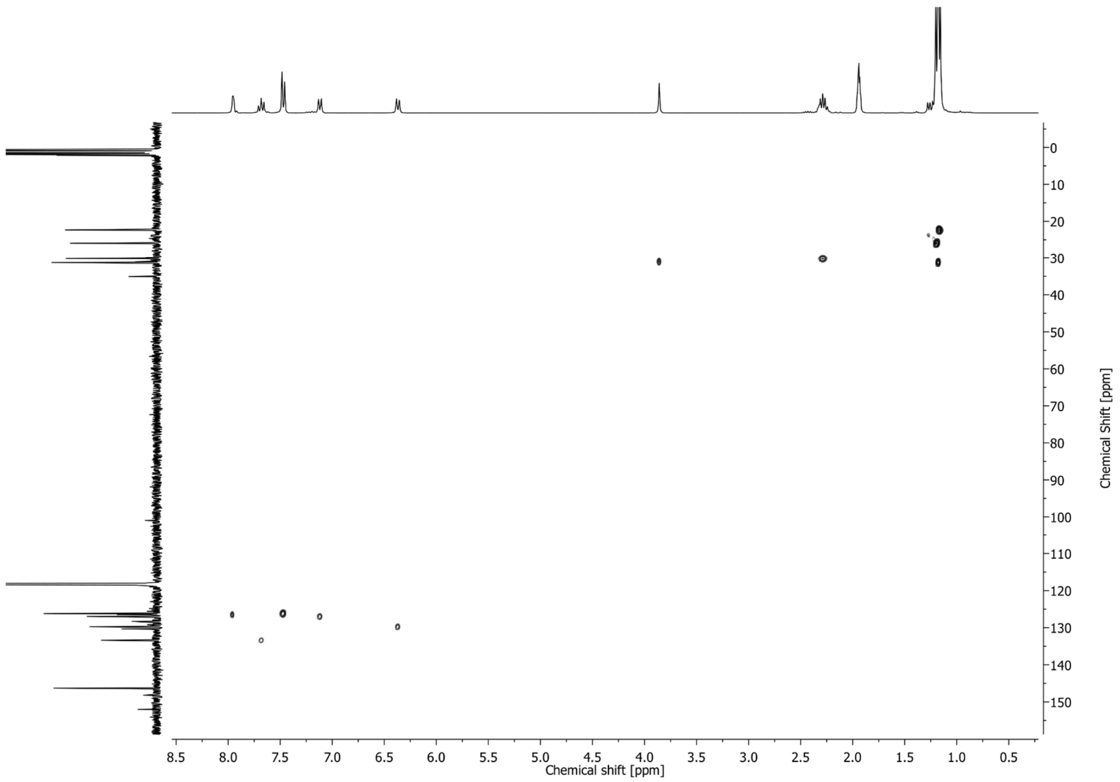

HMBC spectrum of **6** (23 °C, CD_3CN)

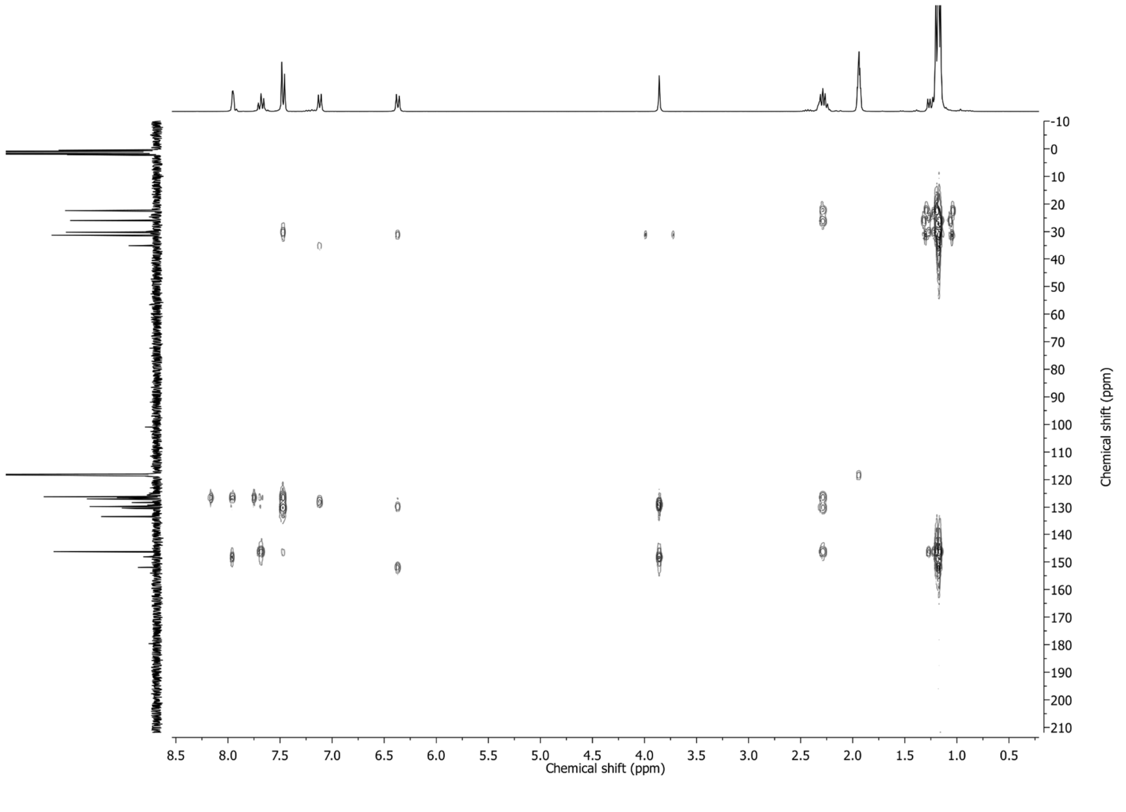

Spectroscopic data of 1-(4-(tert-butyl)benzyl)pyridium-bromide (**14**) is consistent with the proposed structure. Details on synthesis procedure of **14** can be found on Page 41.

^{1}H NMR spectrum of **14** ($CDCl_3$, 29 °C, 300.13 MHz)

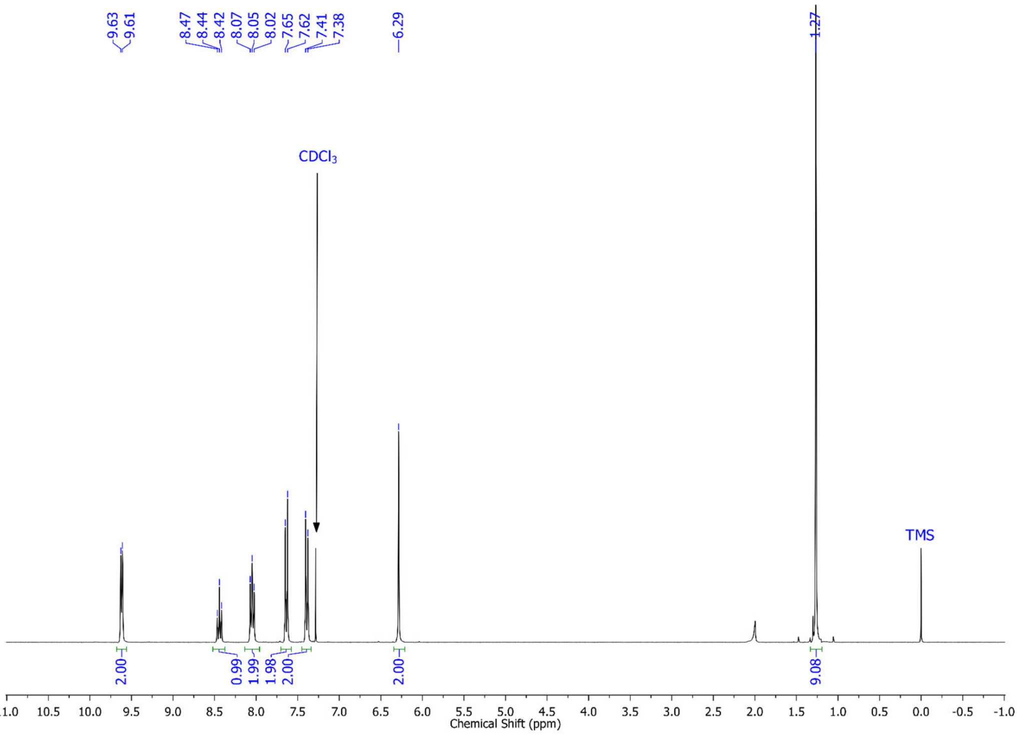

^{13}C NMR spectrum of **14** ($CDCl_3$, 29 °C, 75.47 MHz)

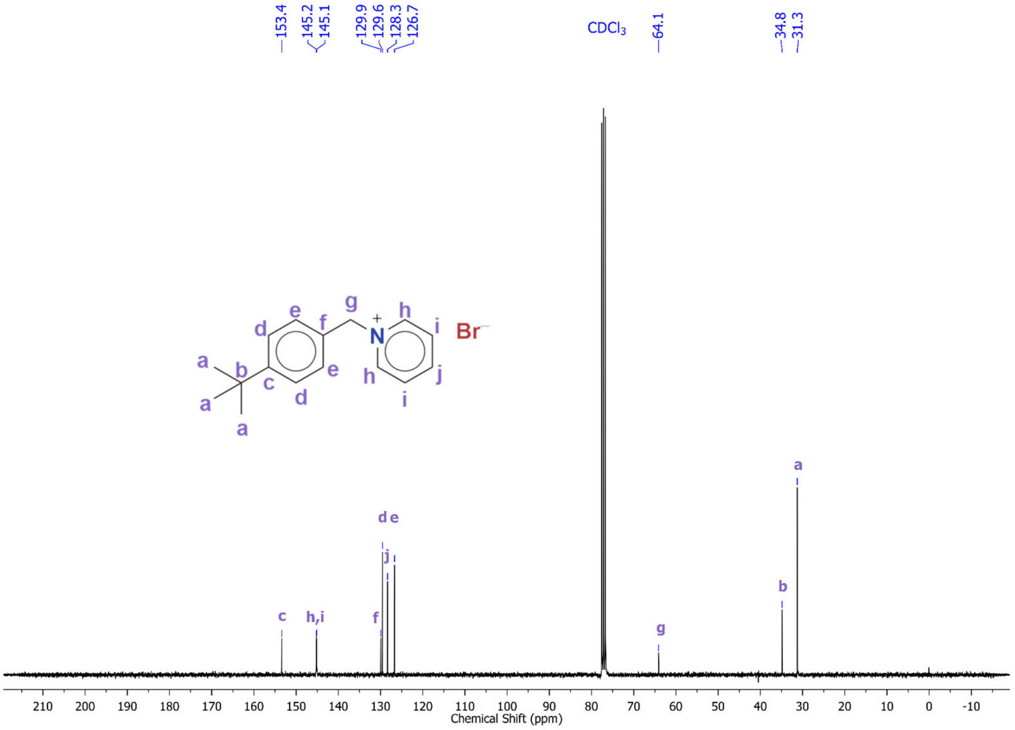

HSQC spectrum of **14** ($CDCl_3$, 23 °C)

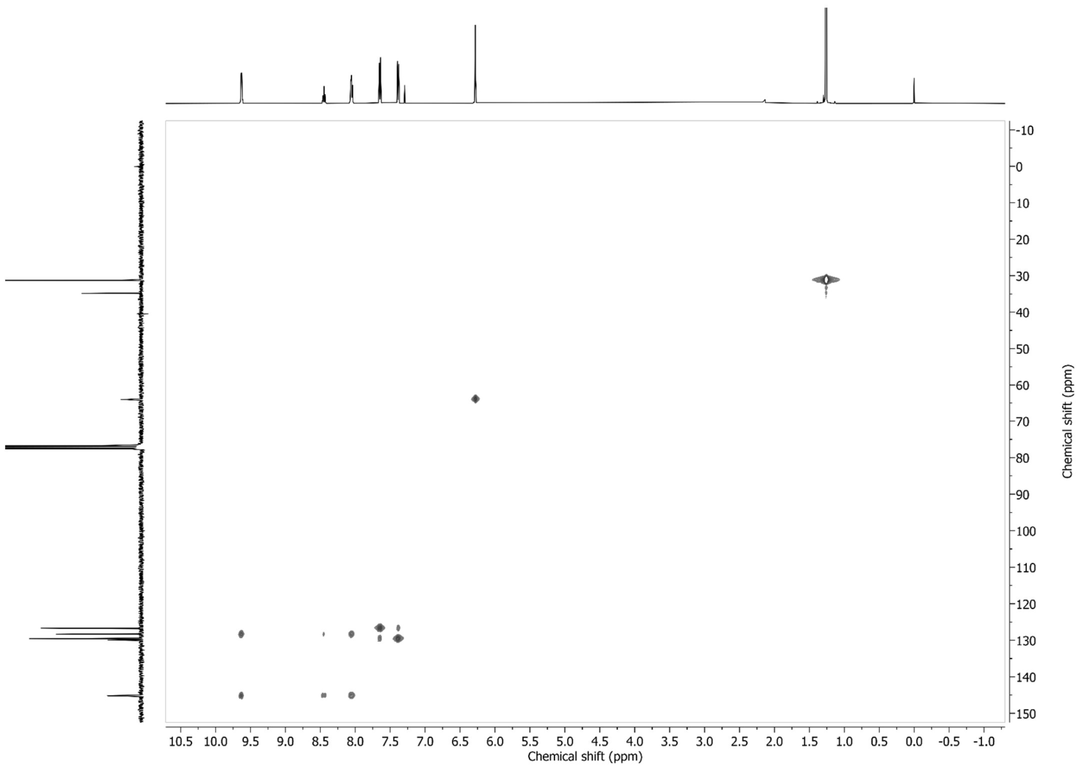

HMBC spectrum of **14** ($CDCl_3$, 23 °C)

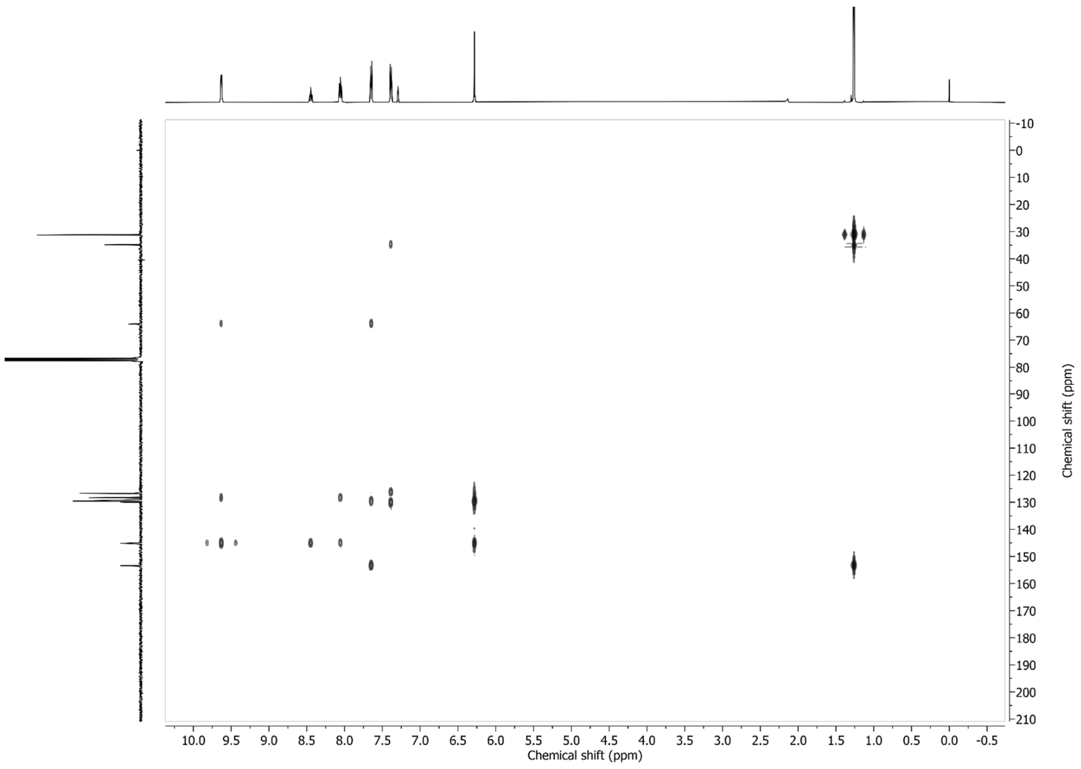

Spectroscopic data of (4-(tert-butyl)benzyl)triethyl-ammonium bromide (15)

is consistent with the proposed structure. Details on synthesis procedure of 15 can be found on Page 42.

^{1}H NMR spectrum of 15 ($CDCl_3$, 29 °C, 300.13 MHz)

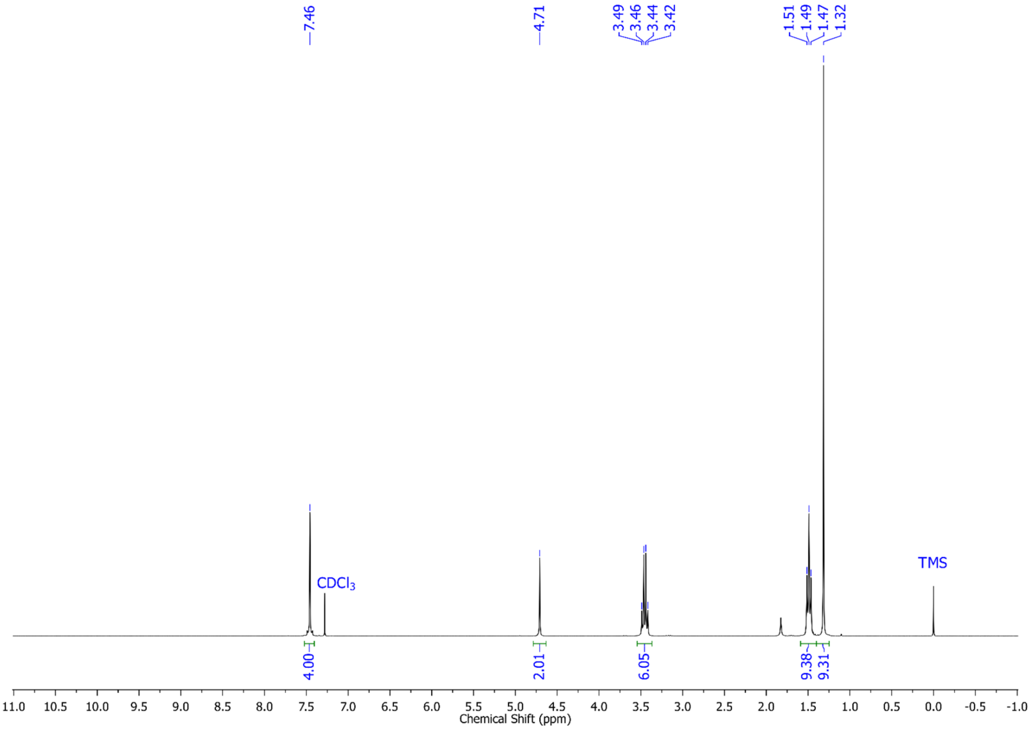

^{13}C NMR spectrum of 15 ($CDCl_3$, 23 °C, 125.76 MHz)

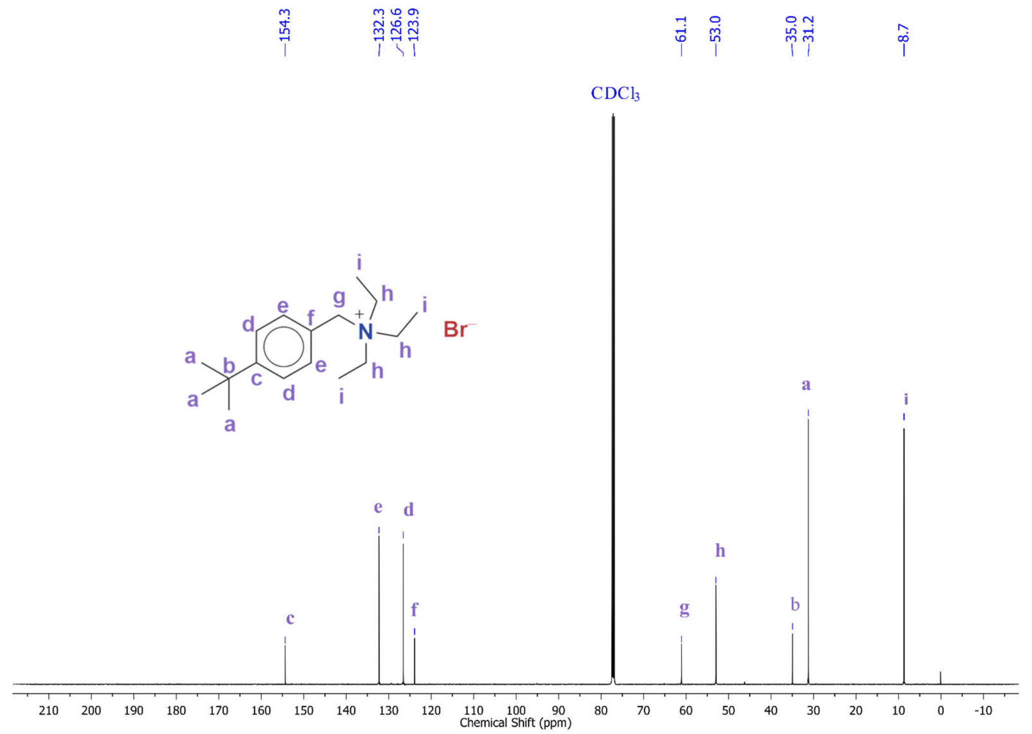

HSQC spectrum of **15** ($CDCl_3$, 23 °C)

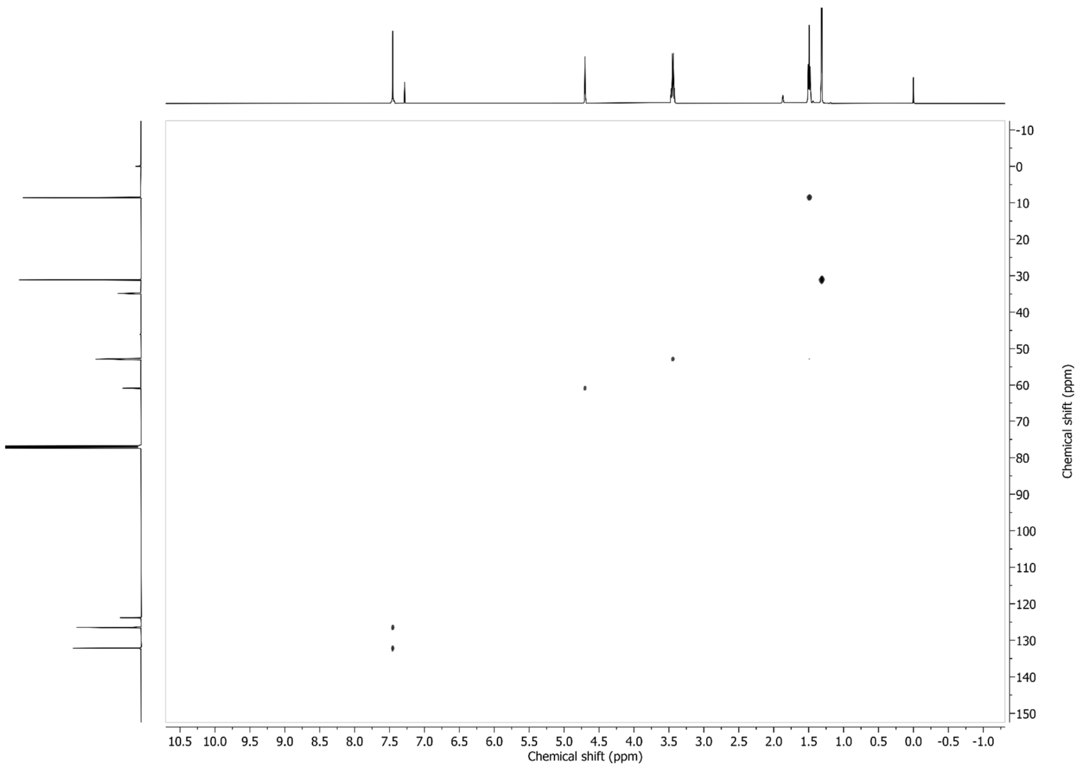

HMBC spectrum of **15** ($CDCl_3$, 23 °C)

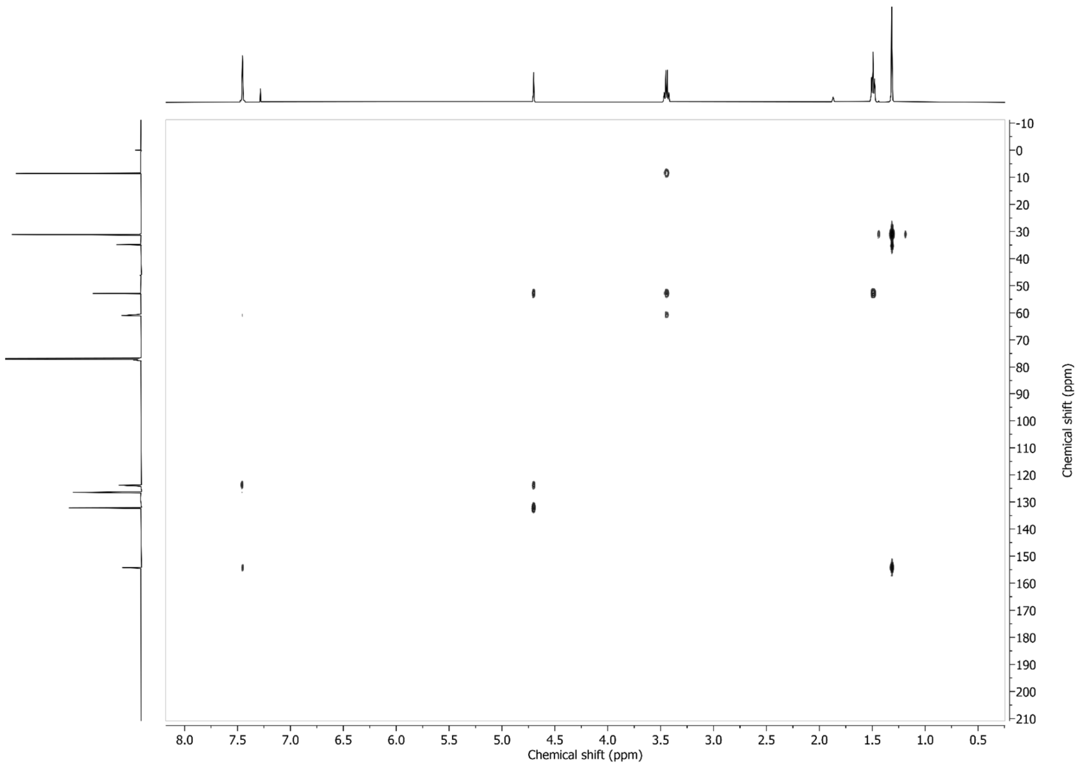

Spectroscopic data of (4-(tert-butyl)benzyl)ethyldiisopropyl-ammonium bromide (16)

is consistent with the proposed structure. Details on synthesis procedure of **16** can be found on Page 42.

^{1}H NMR spectrum of **16** ($CDCl_3$, 29 °C, 300.13 MHz)

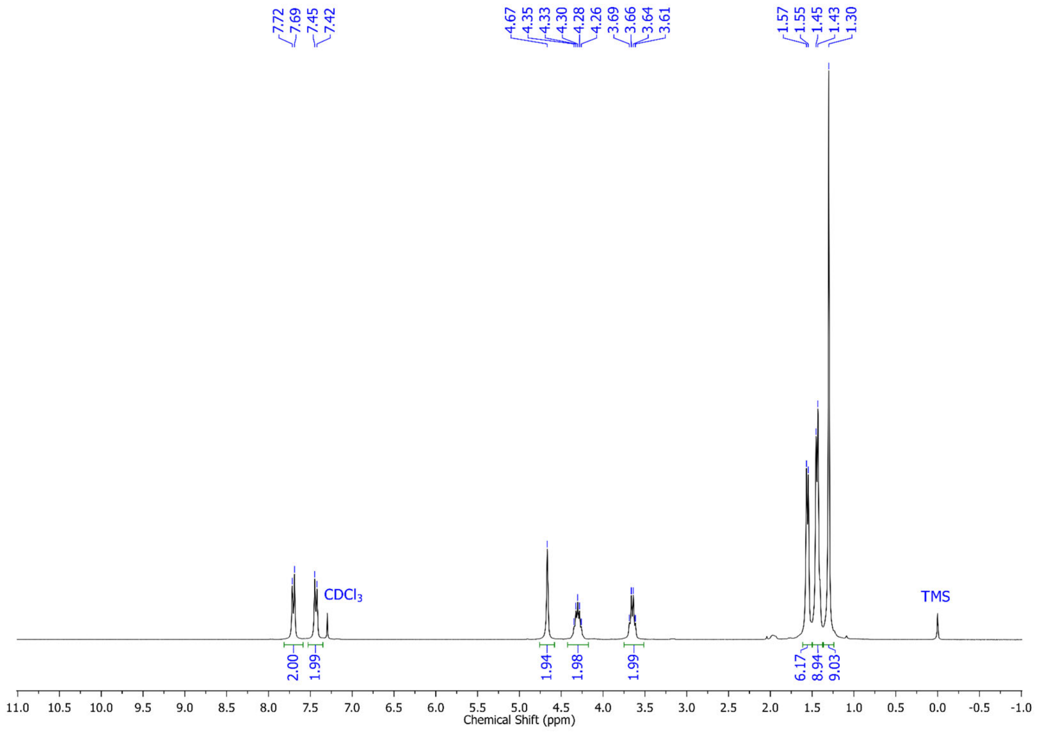

^{13}C NMR spectrum of **16** ($CDCl_3$, 29 °C, 75.47 MHz)

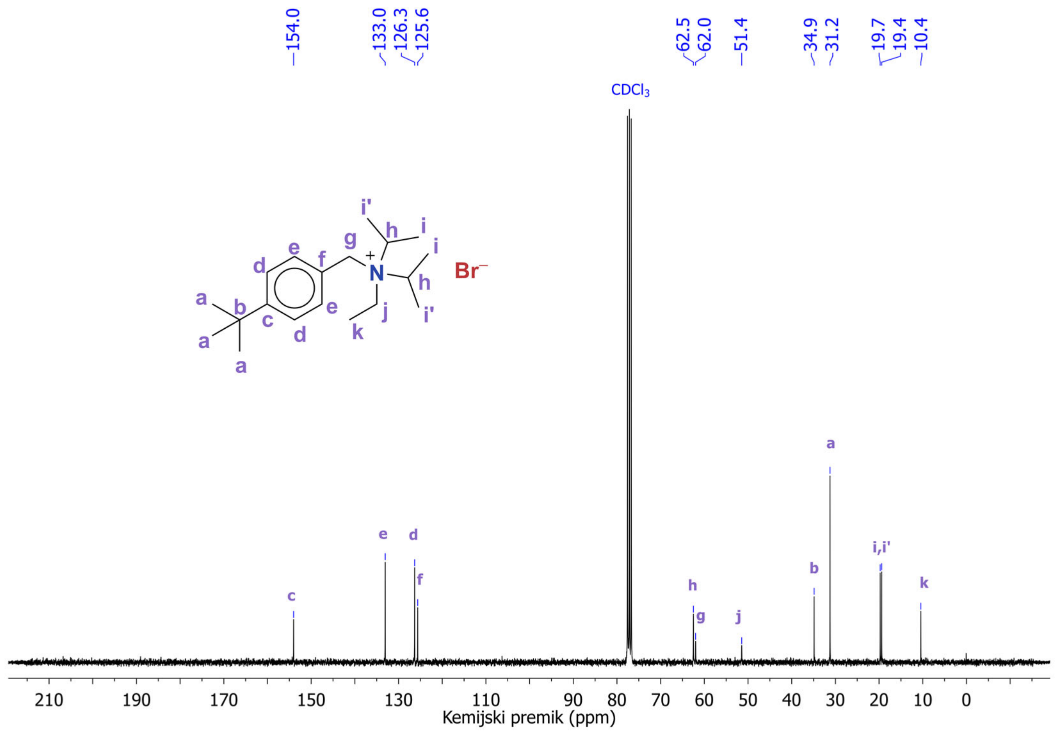

HSQC spectrum of **16** ($CDCl_3$, 23 °C)

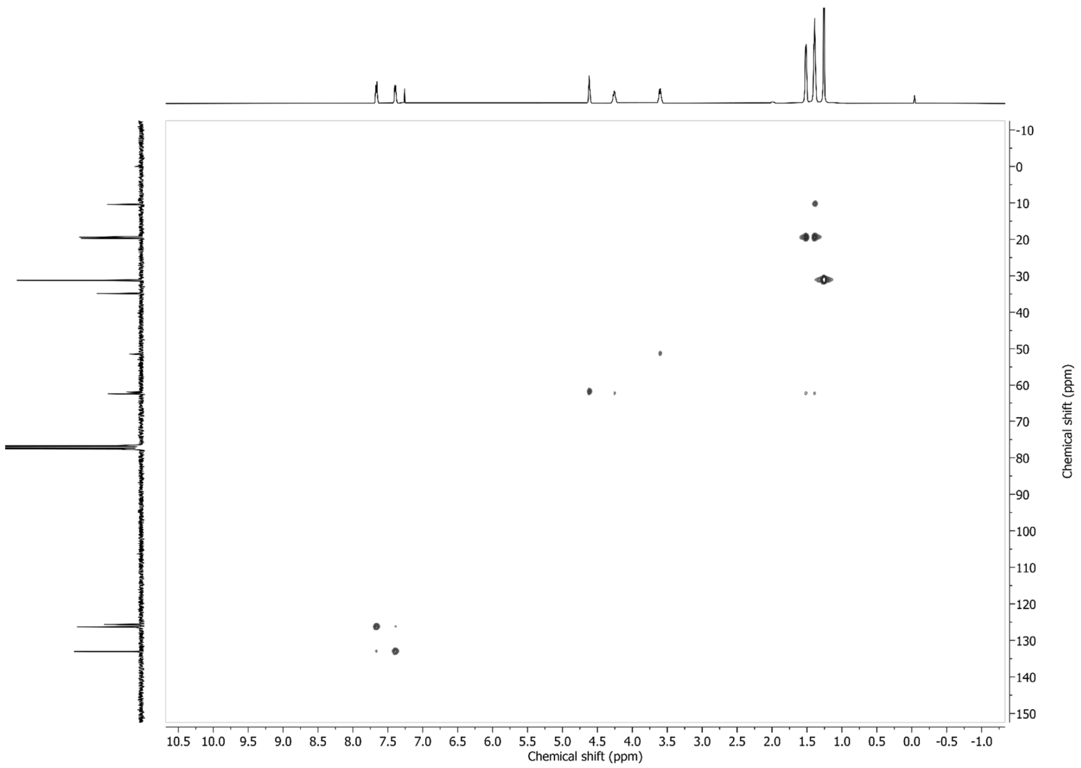

HMBC spectrum of **16** ($CDCl_3$, 23 °C)

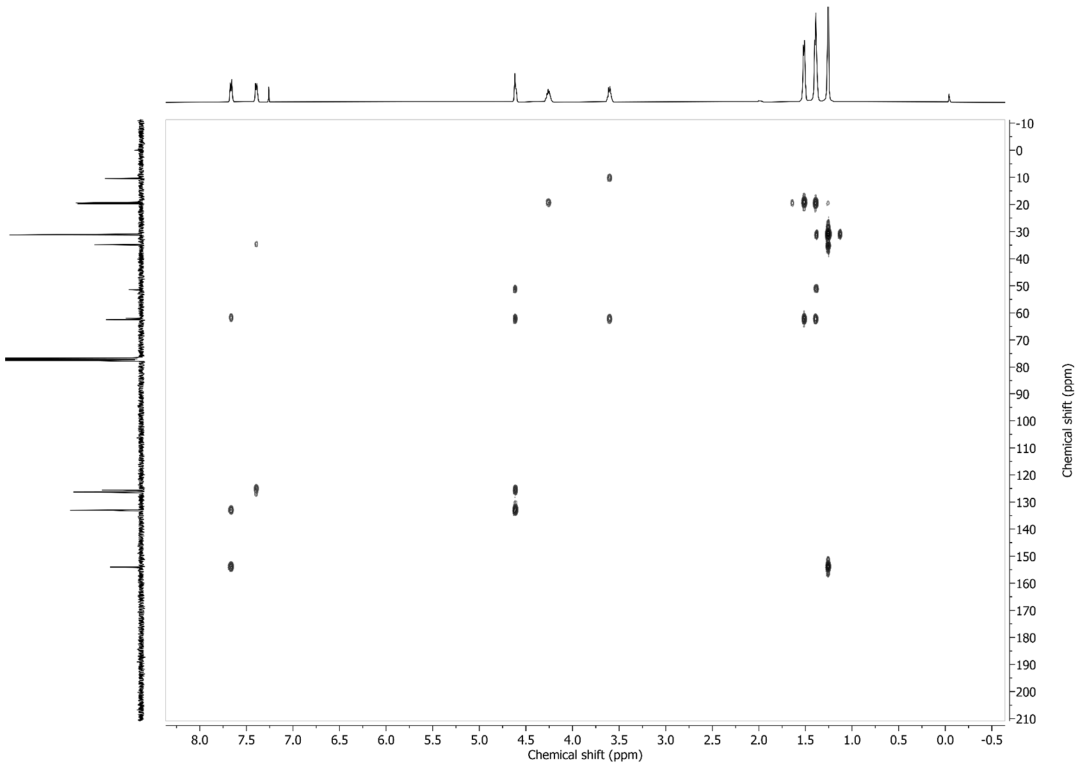

Spectroscopic data of (3-(phenyl)benzyl)ethyldiisopropyl-ammonium bromide (17)

is consistent with the proposed structure. Details on synthesis procedure of **17** can be found on Page 43.

^{1}H NMR spectrum of **17** ($CDCl_3$, 23 °C, 300.13 MHz)

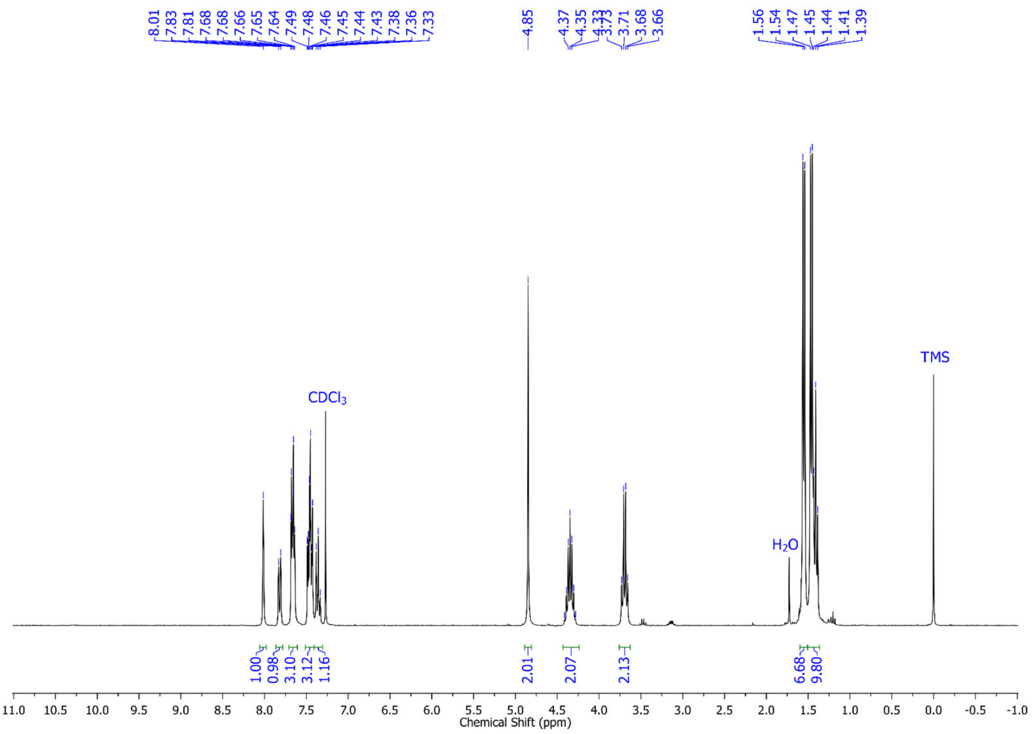

^{13}C NMR spectrum of **17** ($CDCl_3$, 23 °C, 125.76 MHz)

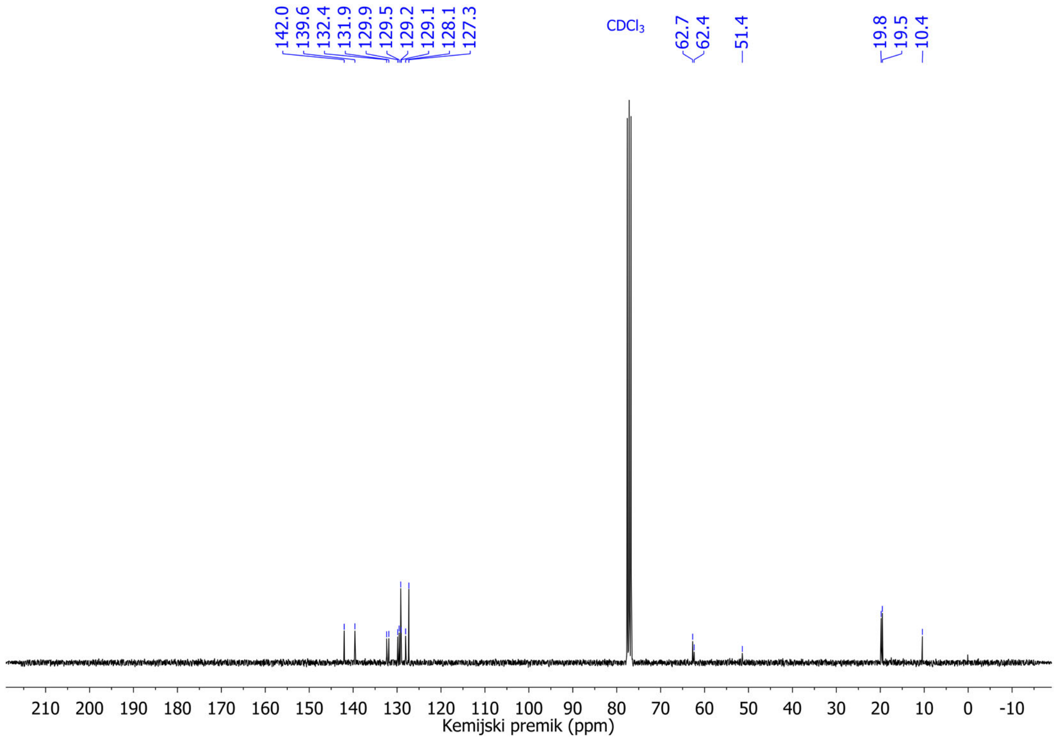

HSQC spectrum of **17** ($CDCl_3$, 23 °C)

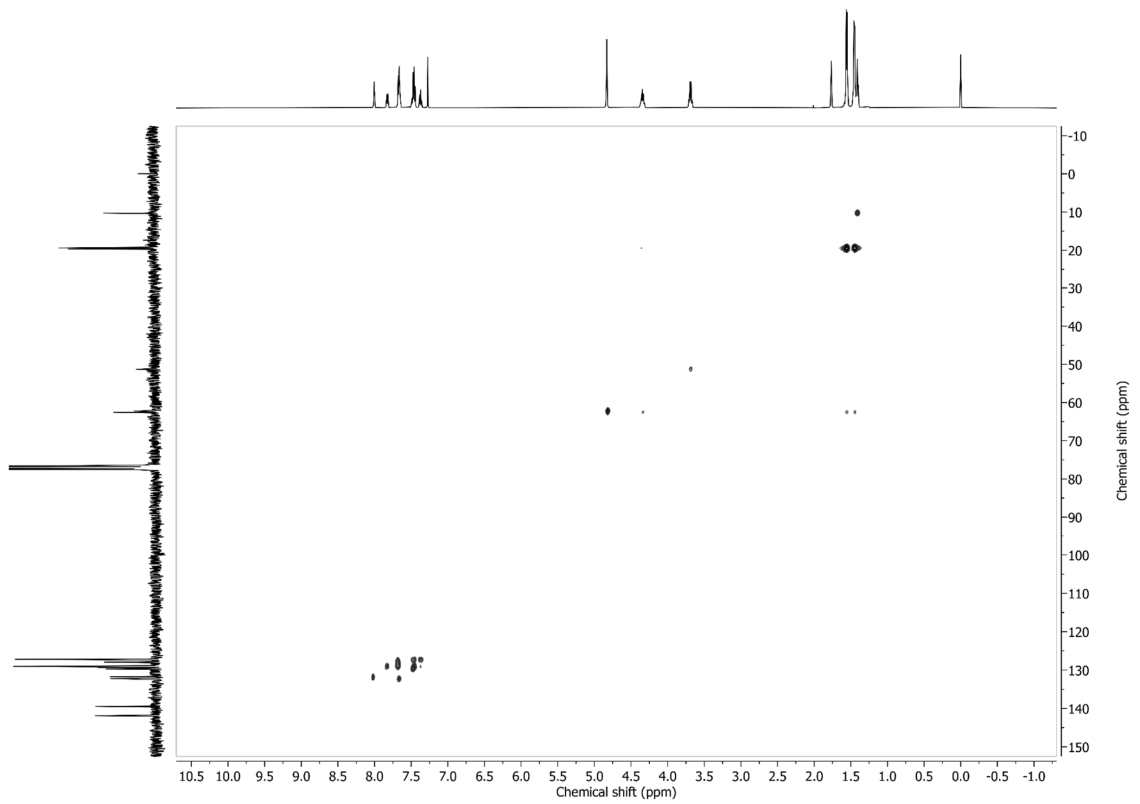

HMBC spectrum of **17** ($CDCl_3$, 23 °C)

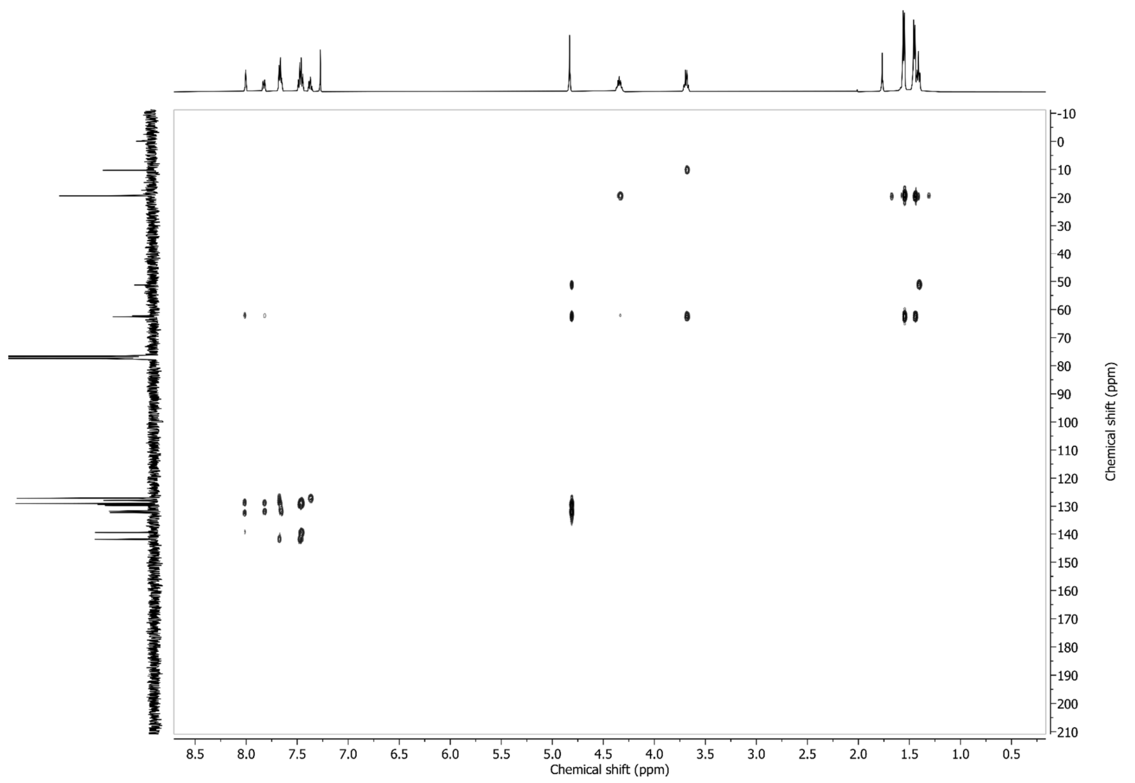

S13 References

1. Hughes, B. 2008 FDA drug approvals. *Nat. Rev. Drug Discov.* **8**, 93–96 (2009).
2. Hughes, B. 2009 FDA drug approvals. *Nat. Rev. Drug Discov.* **9**, 89–92 (2010).
3. Mullard, A. 2010 FDA drug approvals. *Nat. Rev. Drug Discov.* **10**, 82–85 (2011).
4. Mullard, A. 2011 FDA drug approvals. *Nat. Rev. Drug Discov.* **11**, 91–94 (2012).
5. Mullard, A. 2012 FDA drug approvals. *Nat. Rev. Drug Discov.* **12**, 87–90 (2013).
6. Mullard, A. 2013 FDA drug approvals. *Nat. Rev. Drug Discov.* **13**, 85–89 (2014).
7. Mullard, A. 2014 FDA drug approvals. *Nat. Rev. Drug Discov.* **14**, 77–81 (2015).
8. Mullard, A. 2015 FDA drug approvals. *Nat. Rev. Drug Discov.* **15**, 73–76 (2016).
9. Mullard, A. 2016 FDA drug approvals. *Nat. Rev. Drug Discov.* **16**, 73–76 (2017).
10. Mullard, A. 2017 FDA drug approvals. *Nat. Rev. Drug Discov.* **17**, 81–85 (2018).
11. Mullard, A. 2018 FDA drug approvals. *Nat. Rev. Drug Discov.* **18**, 85–89 (2019).
12. Mullard, A. 2019 FDA drug approvals. *Nat. Rev. Drug Discov.* **19**, 79–84 (2020).
13. Hintermann, L. Expedient syntheses of the N-heterocyclic carbene precursor imidazolium salts IPr·HCl, IMes·HCl and IXy·HCl. *Beilstein J. Org. Chem.* **3**, 22 (2007).
14. Jafarpour, L., Stevens, E. D. & Nolan, S. P. A sterically demanding nucleophilic carbene: 1,3-bis(2,6-diisopropylphenyl)imidazol-2-ylidene. Thermochemistry and catalytic application in olefin metathesis. *J. Organomet. Chem.* **606**, 49–54 (2000).
15. Bonnet, S., Limburg, B., Meeldijk, J. D., Klein Gebbink, R. J. M. & Killian, J. A. Ruthenium-Decorated Lipid Vesicles: Light-Induced Release of $[Ru(terpy)(bpy)(OH_2)]^{2+}$ and Thermal Back Coordination. *J. Am. Chem. Soc.* **133**, 252–261 (2011).
16. Sun, Q., Cai, S. & Peterson, B. R. Practical Synthesis of 3*β*-Amino-5-cholestene and Related 3*β*-Halides Involving *i* -Steroid and Retro- *i* -Steroid Rearrangements. *Org. Lett.* **11**, 567–570 (2009).
17. Chidananda, N., Poojary, B., Sumangala, V., Suchetha Kumari, N. & Unnikrishnan. Hantzsch and Schiff's reaction: synthesis, in vitro cytotoxic and antimicrobial activity of [1,3,4]oxadiazoline and [1,3]thiazole derivatives. *Med. Chem. Res.* **23**, 3979–3997 (2014).
18. Davies, H. M. L., Hedley, S. J. & Bohall, B. R. Asymmetric Intermolecular C–H Functionalization of Benzyl Silyl Ethers Mediated by Chiral Auxiliary-Based Aryldiazoacetates and Chiral Dirhodium Catalysts. *J. Org. Chem.* **70**, 10737–10742 (2005).
19. Williams, D. B. G. & Lawton, M. Drying of Organic Solvents: Quantitative Evaluation of the Efficiency of Several Desiccants. *J. Org. Chem.* **75**, 8351–8354 (2010).
20. Champagne, P. A. *et al.* Enabling Nucleophilic Substitution Reactions of Activated Alkyl Fluorides through Hydrogen Bonding. *Org. Lett.* **15**, 2210–2213 (2013).
21. Makino, K. & Yoshioka, H. Selective fluorination of substituted methanols with methanesulfonyl fluoride and cesium fluoride as modified with crown ethers. *J. Fluor. Chem.* **35**, 677–683 (1987).
22. Hu, J., Gao, B., Li, L., Ni, C. & Hu, J. Palladium-Catalyzed Monofluoromethylation of Arylboronic Esters with Fluoromethyl Iodide. *Org. Lett.* **17**, 3086–3089 (2015).

23. Kobayashi, S., Yoneda, A., Fukuhara, T. & Hara, S. Deoxyfluorination of alcohols using *N,N*-diethyl-α,α-difluoro-(*m*-methylbenzyl)amine. *Tetrahedron* 60, 6923–6930 (2004).

24. Chen, J., Lin, J.-H. & Xiao, J.-C. Halogenation through Deoxygenation of Alcohols and Aldehydes. *Org. Lett.* 20, 3061–3064 (2018).

25. Schaefer, T., Schurko, R. W., Sebastian, R. & Hruska, F. E. Experimental and theoretical assessments of the substituent and medium dependence of the internal rotational potentials in benzyl fluoride. 3,5-Difluorobenzyl fluoride and 4-fluorobenzyl fluoride. *Can. J. Chem.* 73, 816–825 (1995).

26. An, L., Xiao, Y.-L., Min, Q.-Q. & Zhang, X. Facile Access to Fluoromethylated Arenes by Nickel-Catalyzed Cross-Coupling between Arylboronic Acids and Fluoromethyl Bromide. *Angew. Chem. Int. Ed.* 54, 9079–9083 (2015).

27. Blessley, G., Holden, P., Walker, M., Brown, J. M. & Gouverneur, V. Palladium-Catalyzed Substitution and Cross-Coupling of Benzylic Fluorides. *Org. Lett.* 14, 2754–2757 (2012).

28. Kulkarni, K. G., Miokovic, B., Sauder, M. & Murphy, G. K. Denitrogenative hydrofluorination of aromatic aldehyde hydrazones using (difluoroiodo)toluene. *Org. Biomol. Chem.* 14, 9907–9911 (2016).

29. Adcock, W. & Abeywickrema, A. Conformational preference of the fluoromethyl group in some benzyl fluorides: A ^{13}C N.M.R. study. *Aust. J. Chem.* 33, 181 (1980).

30. Ochiai, M. *et al.* Oxidation of Primary Aliphatic and Aromatic Aldehydes with Difluoro(aryl)-λ^3-bromane. *Org. Lett.* 13, 5568–5571 (2011).

31. Liu, Y. *et al.* Efficient SN2 Fluorination of Primary and Secondary Alkyl Bromides by Copper(I) Fluoride Complexes. *Organometallics* 32, 6587–6592 (2013).

32. SDBS. 1-octene. Available at: https://sdbs.db.aist.go.jp/sdbs/cgi-bin/landingpage?sdbsno=788. (Accessed: 9th October 2019)

33. Cousseau, J. & Albert, P. Nucleophilic fluorine displacement reactions. A comparison of reactivities of polymer-supported fluoride and acid fluorides P^+F^-, *n*HF (*n* = 0-2). *J. Org. Chem.* 54, 5380–5383 (1989).

34. Cruz Silva, M. M., Riva, S. & Sá e Melo, M. L. Regioselective enzymatic acylation of vicinal diols of steroids. *Tetrahedron* 61, 3065–3073 (2005).

35. Chochrek, P. & Wicha, J. 1,3-Chirality Transfer by Fragmentation of Allylsulfinic Acids: A Diastereoselective Approach to Vinyl Bromides Related totrans-Hydrindane ortrans-Decalin. *European J. Org. Chem.* 2007, 2534–2542 (2007).

36. Yang, Q., Mao, L.-L., Yang, B. & Yang, S.-D. Metal-Free, Efficient Oxyfluorination of Olefins for the Synthesis of α-Fluoroketones. *Org. Lett.* 16, 3460–3463 (2014).

37. Takale, B. S. *et al.* Exclusive Chemoselective Reduction of Imines in the Coexistence of Aldehydes Using AuNPore Catalyst. *Org. Lett.* 16, 2558–2561 (2014).

38. Hairston, T. J. & O'Brien, D. H. The reaction of (α-haloalkyl)silanes with Lewis acids. *J. Organomet. Chem.* 29, 79–92 (1971).

39. Mukherjee, H. *et al.* A study of the reactivity of $S^{(VI)}$–F containing warheads with nucleophilic amino-acid side chains under physiological conditions. *Org. Biomol. Chem.* 15, 9685–9695 (2017).

40. Manna, S., Maity, S., Rana, S., Agasti, S. & Maiti, D. *ipso*-Nitration of Arylboronic Acids with Bismuth Nitrate and Perdisulfate. *Org. Lett.* **14**, 1736–1739 (2012).

41. Alič, B. & Tavčar, G. Reaction of *N*-heterocyclic carbene (NHC) with different HF sources and ratios – A free fluoride reagent based on imidazolium fluoride. *J. Fluor. Chem.* **192**, 141–146 (2016).

42. Knappke, C. E. I. *et al.* On new *N*-heterocyclic carbene derived alkylidene imidazolines. *Org. Biomol. Chem.* **8**, 1695 (2010).

43. Allen, F. H. *et al.* Tables of bond lengths determined by X-ray and neutron diffraction. Part 1. Bond lengths in organic compounds. *J. Chem. Soc. Perkin Trans. 2* S1–S19 (1987). doi:10.1039/p298700000s1

44. Bernstein, J., Roth, J. S. & Miller, W. T. The Preparation and Properties of Some Substituted Benzyl Fluorides [1]. *J. Am. Chem. Soc.* **70**, 2310–2314 (1948).

45. Takadate, A., Tahara, T. & Goya, S. A Convenient Preparation of Arylmonofluoromethanes. *Synthesis (Stuttg).* **1983**, 806–807 (1983).

46. Herrmann, P., Kvíčala, J., Pouzar, V. & Chodounská, H. Synthesis of Fluorinated Steroids Using a Novel Fluorinating Reagent Tetrabutylammonium Difluorodimethylphenylsilicate (TAMPS). *Collect. Czechoslov. Chem. Commun.* **73**, 1825–1834 (2008).
